# Supplementary material for: Single-nucleus RNA sequencing reveals glial cell type-specific responses to ischemic stroke in male rodents
Source: Nat Commun. 2024 Jul 24;15:6232. doi: 10.1038/s41467-024-50465-z (PMC11266704; doi:10.1038/s41467-024-50465-z)
Supplement: Supplementary file 1 — Supplementary Information [file 41467_2024_50465_MOESM1_ESM.pdf]

Single nucleus RNA sequencing reveals glial cell type-specific responses to ischemic stroke

# - Supplementary information

Daniel Bormann[1,2], Michael Knoflach[3,4], Emilia Poreba[5], Christian J. Riedl[6], Giulia Testa[6], Cyrille Orset[7,8], Anthony Levilly[7,8], Andreá Cottereau[7,8], Phillip Jauk[6,9], Simon Hametner[6], Nadine Stranzl[6], Bahar Golabi[5], Dragan Copic[1,2,10], Katharina Klas[1,2], Martin Direder[1,2,11], Hannes Kühtreiber[1,2], Melanie Salek[1,2], Stephanie zur Nedden[12], Gabriele Baier-Bitterlich[12], Stefan Kiechl[3,4], Carmen Haider[6], Verena Endmayr[6], Romana Höftberger[6], Hendrik J. Ankersmit[1,2]*, Michael Mildner[5]*

[1] Applied Immunology Laboratory, Department of Thoracic Surgery, Medical University of Vienna, 1090 Vienna, Austria
[2]Aposcience AG, 1200 Vienna, Austria
[3]Department of Neurology, Medical University of Innsbruck, Anichstraße 35, 6020 Innsbruck, Austria
[4] VASCage, Research Centre on Vascular Ageing and Stroke, 6020 Innsbruck, Austria
[5]Department of Dermatology, Medical University of Vienna, 1090 Vienna, Austria
[6]Division of Neuropathology and Neurochemistry, Department of Neurology, Medical University of Vienna, 1090 Vienna, Austria
[7]Normandie University, UNICAEN, ESR3P, INSERM UMR-S U1237, Physiopathology and Imaging of Neurological Disorders (PhIND), GIP Cyceron, Institut Blood and Brain @ Caen-Normandie (BB@C), Caen, France
[8]Department of Clinical Research, Caen-Normandie University Hospital, Caen, France
[9]Center for Medical Physics and Biomedical Engineering, Medical University of Vienna, 1090 Vienna, Austria
[10]Division of Nephrology and Dialysis, Department of Internal Medicine III, Medical University of Vienna, 1090 Vienna, Austria.
[11]Department of Orthopedics and Trauma Surgery, Medical University of Vienna, 1090 Vienna, Austria
[12]Institute of Neurobiochemistry, CCB-Biocenter, Medical University of Innsbruck, 6020 Innsbruck, Austria
*: contributed equally

Correspondence to:

Michael Mildner, PhD
Department of Dermatology, Medical University of Vienna
Lazarettgasse 14, 1090 Vienna, Austria
phone: +43-(0)1-40400-73507
e-mail: michael.mildner@meduniwien.ac.at
or
Hendrik Jan Ankersmit, MD, MBA
Applied Immunology Laboratory, Department of Thoracic Surgery, Medical University of Vienna, Waehringer Guertel 18-20, 1090 Vienna, Austria.
e-mail: hendrik.ankersmit@meduniwien.ac.at

Table of contents

Supplementary figures

Supplementary Figure 1. Standardized tissue sampling and MRI validation of cerebral infarction

Supplementary Figure 2. Hyperintense lesions in T2-weighted MRI data correlate closely with histologically defined myelin and neuronal loss

Supplementary Figure 3. snRNAseq quality control and nuclei distribution across samples.

Supplementary Figure 4. A detailed transcriptional analysis of myeloid cells enriched within infarcted brain tissue.

Supplementary Figure 5. Comparison of myeloid cell sub clusters derived from moderately and severely infarcted brain tissue.

Supplementary Figure 6. Global distribution of MCAO induced differentially expressed genes (DEGs) across all major cell clusters.

Supplementary Figure 7. DEG signatures of neurons within infarcted brain tissue.

Supplementary Figure 8. Additional pre-processing steps pertaining to Figure 2

Supplementary Figure 9. Analysis of DEGs in conserved oligodendrocyte lineage sub clusters.

Supplementary Figure 10. Nuclei distribution and transcriptional profiles of oligodendrocyte sub clusters, across samples.

Supplementary Figure 11. Enrichment analysis of OPC_1 marker genes.

Supplementary Figure 12. Enrichment analysis of MOLIGO_1 marker genes.

Supplementary Figure 13. Limited transcriptional overlap between stroke associated oligodendrocyte lineage cells and diseases associated oligodendrocytes (DAO).

Supplementary Figure 14. Expression of curated OPC_1 and MOLIGO_1 marker DEGs within each sample

Supplementary Figure 15. Proliferating, VIM positive OPCs are found in the lesion core 48h after filament based permanent MCAO in rats.

Supplementary Figure 16. MRI validation of cerebral infarction and histological characterization of ischemic lesions 48h after thromboembolic MCAO in mice.

Supplementary Figure 17. Proliferating, VIM positive OPCs accumulate in the perilesional zone 48h after thromboembolic MCAO in mice.

Supplementary Figure 18. Proliferating, IL33 positive OPCs are found in the perilesional grey matter, affected white matter and lesion core 48h after filament based permanent MCAO in rats

Supplementary Figure 19. Proliferating OPCs do not upregulate IL33 within infarcted hemispheres 48h after thromboembolic MCAO in mice.

Supplementary Figure 20. Additional pre-processing steps pertaining to Figure 4.

Supplementary Figure 21. Nuclei distribution and transcriptional profiles of astrocyte subclusters across samples.

Supplementary Figure 22. Enrichment analysis of AC_3 marker genes.

Supplementary Figure 23. Enrichment analysis of AC_4 marker genes.

Supplementary Figure 24. Enrichment analysis of AC_5 marker genes.

Supplementary Figure 25. Transcriptional signatures of reactive astrocytes in stroke compared to other neuropathologies.

Supplementary Figure 26. Cell-cell communication analysis infers immuno-glial cross talk within infarcted brain tissue.

Supplementary Figure 27. Cell-cell communication analysis infers intra-glial cross talk within infarcted brain tissue.

Supplementary Figure 28. Identification of CD44 positive reactive astrocytes and OPCs within the lesion core, 48h after filament based permanent MCAO in rats.

Supplementary Figure 29. Reactive astrocytes and proliferating OPCs are CD44 positive and abundant in the perilesional zone 48 h after thromboembolic MCAO in mice.

Supplementary Figure 30. Identification of osteopontin positive myeloid cells within the lesion core, 48h after filament based permanent MCAO in rats.

Supplementary Figure 31. Osteopontin positive myeloid cells accumulate in the perilesional zone in close proximity to CD44 positive cells 48 h after thromboembolic MCAO in mice.

Supplementary figure 32. Abundant proliferating, CD44 positive myeloid cells are identified in the perilesional zone and lesion core.

Supplementary figure 33. Spatial association of osteopontin positive myeloid cells to CD44 positive cells in human infarcted cerebral tissue.

<u>Supplementary tables</u>

Supplementary Table 1. Allen Brain Atlas database in situ hybridization (ISH) studies referenced within this study.

Supplementary Table 2. Antibodies and antibody labelling kits used in this study.

Supplementary Table 3. Antibody combinations, Heat induced epitope retrieval (HIER) pH and antibody dilutions.

<u>Supplementary notes – Detailed description of cluster annotation</u>

Supplementary References

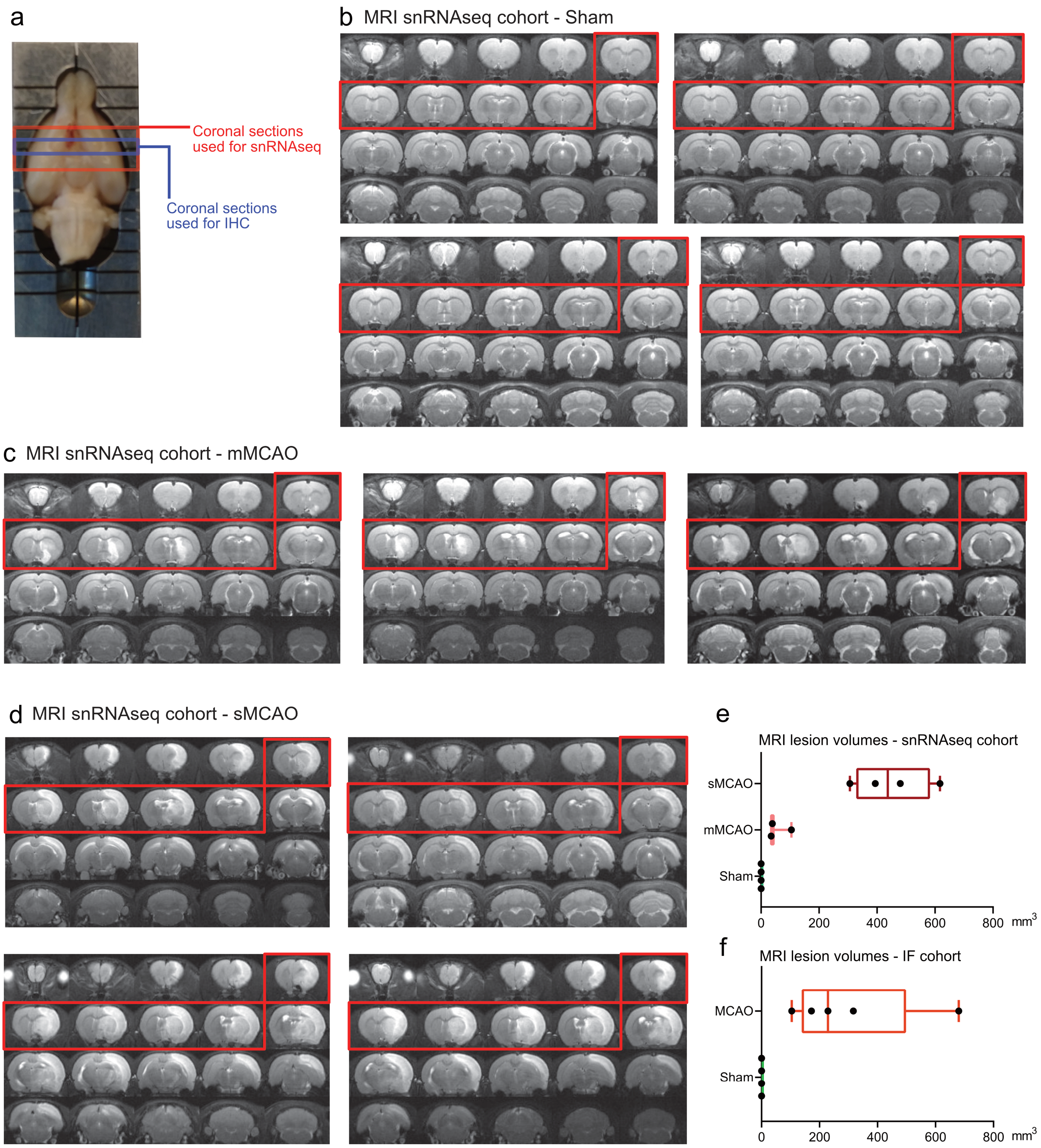

Supplementary Figure 1. Standardized tissue sampling and MRI validation of cerebral infarction. a Representative photograph of a rat brain placed in the adult rat brain slicer matrix, used for dissecting coronal brain sections. The red box denotes the brain region sampled for snRNAseq studies, the blue box depicts the brain region used for immunofluorescence staining. **b-d** T2-weighted MRI images of all animals used for snRNAseq in the Sham group (n=4) (**b**), as well as MCAO group (n=7) (**c** and **d**) are shown, hyper intense areas demark infarcted tissue. The MCAO group was further subdivided in cases with moderate (mMCAO) (n=3) (**c**) and severe (sMCAO) (n=4) infarctions (**d**). **e-f** Distributions of overall infarct lesion sizes in the rat cohorts used for snRNAseq (**e**) and immunofluorescence staining (**f**) are presented as boxplots, depicting medians, 25th to 75th percentiles as hinges, minimal and maximal values as whiskers, and individual lesion volumes for each animal as dots.

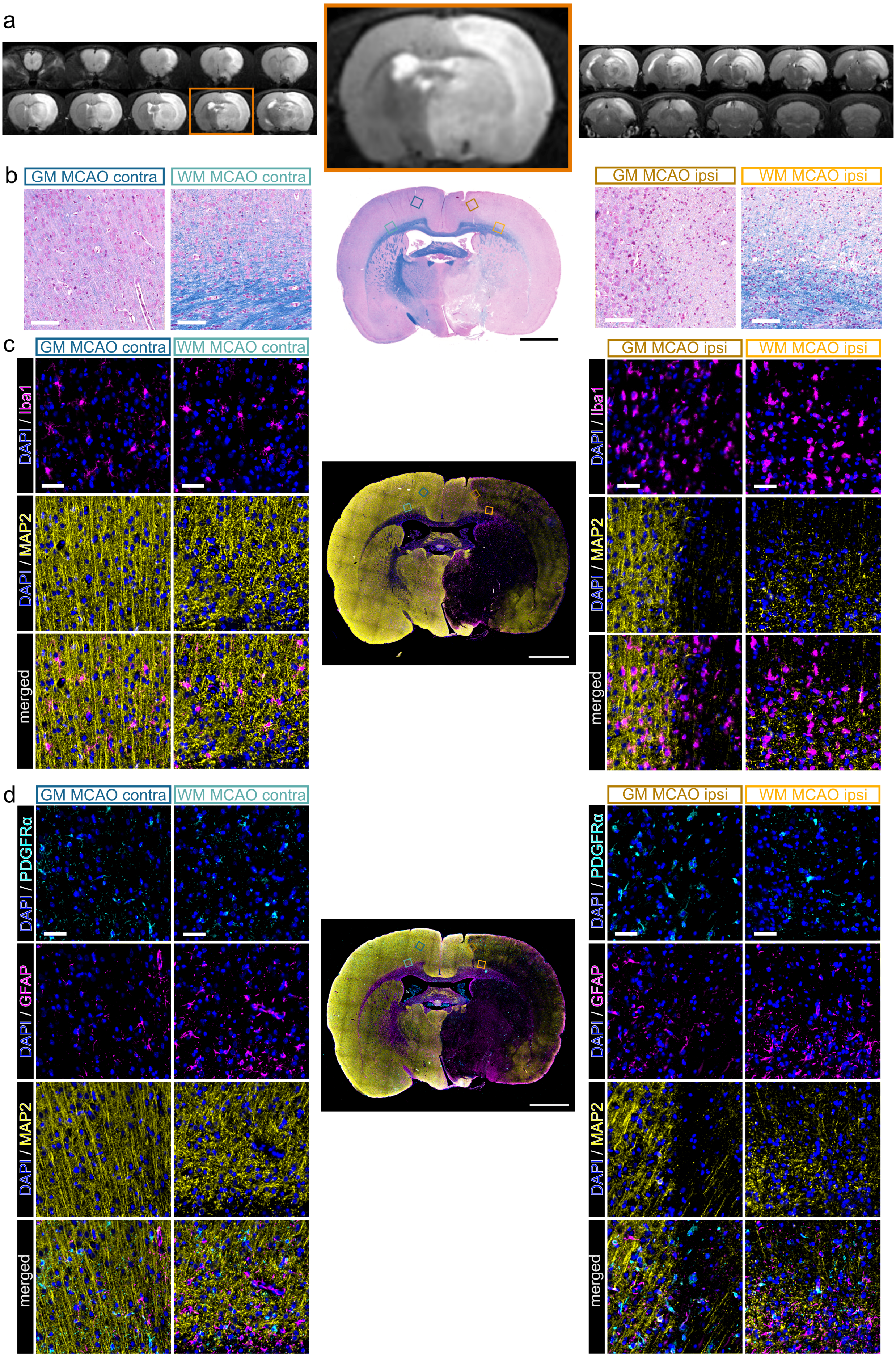

Supplementary Figure 2. (Legend on next page)

Supplementary Figure 2. Hyperintense lesions in T2-weighted MRI data correlate closely with histologically defined myelin and neuronal loss. a T2-weighted MRI image series from a rat brain 48h post filament based permanent middle cerebral artery occlusion (MCAO). Histological and immunofluorescence based stainings in **b-d** were performed on a coronal brain section from the same rat and correspond to the MRI section highlighted in orange. **b** Luxol Fast Blue staining of a coronal section from a rat brain 48h post MCAO, visualizing myelin loss. Close up images depict perilesional grey matter (GM) and affected white matter (WM) regions bordering infarcted tissue (GM MCAO ispi and WM MCAO ipsi, respectively), as well as anatomically corresponding regions of the hemisphere contralateral to infarction (GM MCAO contra and WM MCAO contra, respectively). Scale bars: Black scale bar in overview image: 2mm, white scale bars in close up images: 100µm. **c-d** Immunofluorescence based colocalization of (**c**) MAP2 (yellow) and Iba1 (magenta), as well as (**d**) MAP2 (yellow), GFAP (magenta) and PDGFRα (cyan) in a coronal section from a rat brain 48h post MCAO, visualizing neuronal loss and presence of myeloid and glial cells at the perilesional zone. Close up images depict perilesional grey matter (GM) and affected white matter (WM) regions bordering infarcted tissue (GM MCAO ispi and WM MCAO ipsi, respectively), as well as anatomically corresponding regions of the hemisphere contralateral to infarction (GM MCAO contra and WM MCAO contra, respectively). Scale bars: Scale bars in overview images: 2mm, Scale bars in close up images: 50µm.

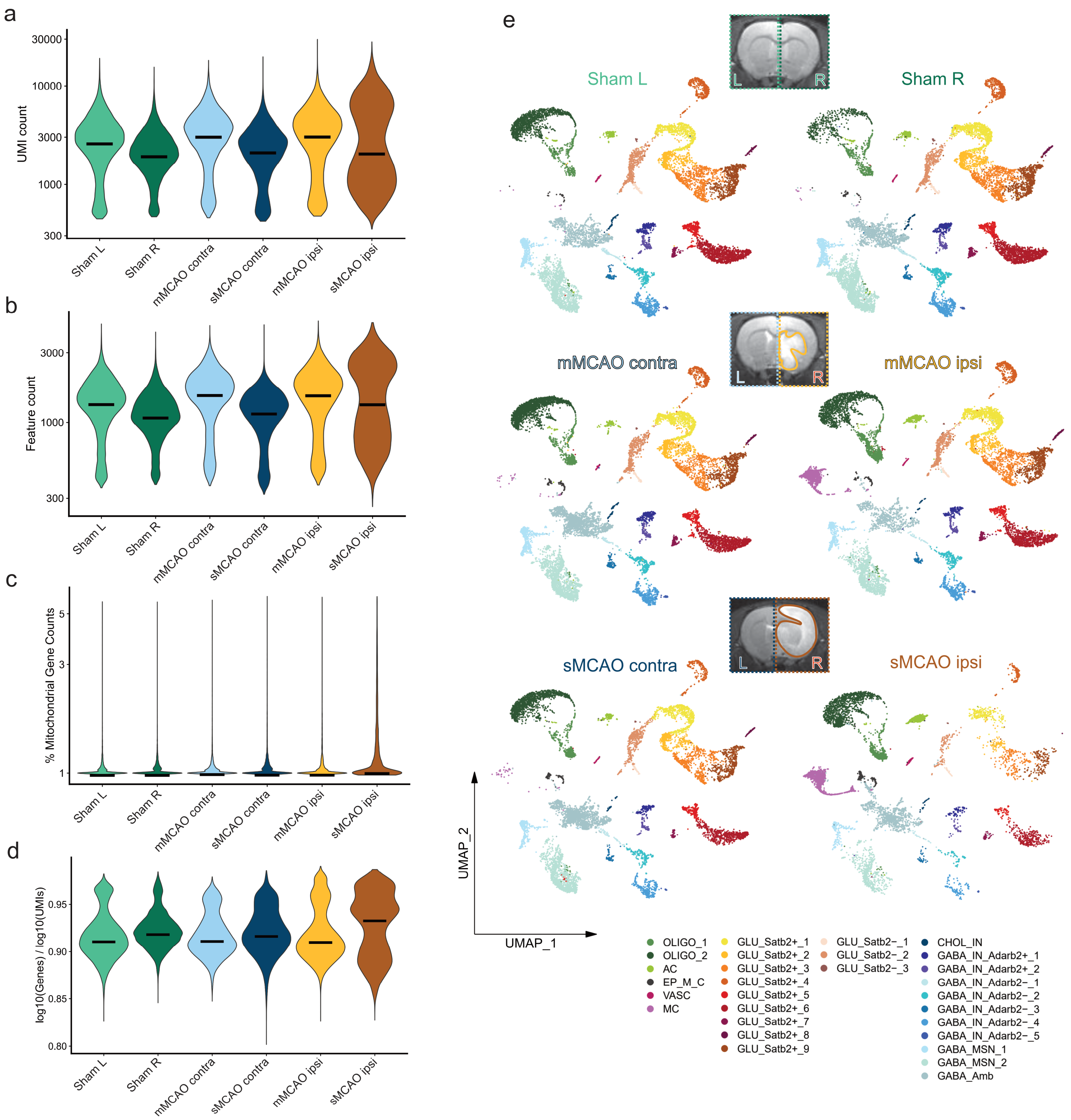

Supplementary Figure 3. snRNAseq quality control and nuclei distribution across samples. a-d Major quality control metrics reported for the final integrated dataset, derived from n=4 Sham control rats and n=7 middle cerebral artery occlusion (MCAO) group rats (n=3 moderate, n=4 severe infarctions). **a** Number of unique transcripts (=UMIs), **b** genes (=features), **c** percentages of reads aligned to mitochondrial genes and **d** the ratio of log10 of gene counts to log10 UMI counts (novelty score). All metrics are reported for each sequenced sample, with y axes scales log normalized. Black bars denote median values. **e** UMAP plots depict distribution of nuclei across major cell clusters, split by individual samples. T2-weighted MRI images show representative coronal brain sections of Sham group rats (top) and MCAO group rats with moderate (mMCAO) (middle) and severe infarctions (sMCAO) (bottom).

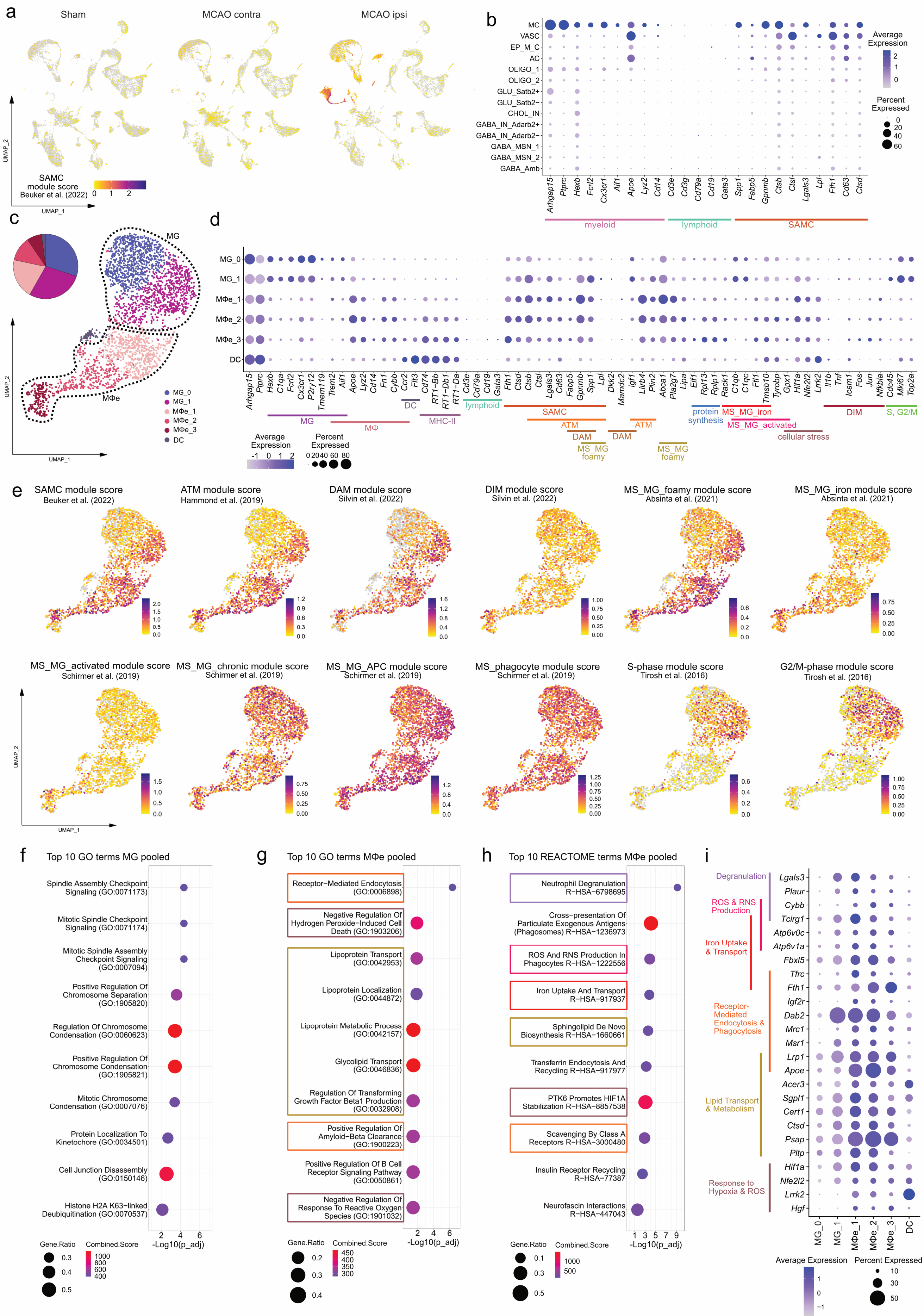

Supplementary Figure 4. (Legend on next page)

Supplementary Figure 4. A detailed transcriptional analysis of myeloid cells enriched within infarcted brain tissue. **a** Feature plots showing stroke associated myeloid cell (SAMC) gene module score expression projected onto main clustering UMAP plots, split by treatment group. **b** Dotplot depicting expression of canonical myeloid and lymphoid cell lineage markers, as well as the genes making up the SAMC module gene set within the main clusters of the integrated dataset. **c** Subclustering of myeloid cells derived from infarcted tissue (MCAO ipsi). UMAP plot depicting 2646 nuclei annotated to 6 sub clusters. The relative contribution of each subcluster to all MCAO ipsi enriched myeloid cells is presented as pie plot. **d** Dotplot depicting the expression of canonical microglia, macrophage, DC, MHC-II and lymphoid cell associated genes, as well as representative genes from previously described MC gene sets within each myeloid subcluster. **e** Module score feature plots projecting aggregate expressions of various gene sets on to MC subcluster UMAP plots. Abbreviations: MG: microglia, MΦ: macrophage, MΦe: macrophage enriched, DC: dendritic cell, MHC: major histocompatibility complex, SAMC: stroke associated myeloid cell, ATM: Axon Tract-Associated Microglia, DAM: disease-associated microglia, MS_MG: Multiple sclerosis associated microglia, DIM: disease inflammatory macrophage, APC: antigen-presenting cell. **f-i** Results of functional enrichment analyses of MG and MΦe cluster marker genes (log2FC >= 0.6, Bonferroni-adjusted p-values < 0.05) are depicted as dot plots. Top 10 enriched GO biological process terms, subset by combined score and order by $-\log_{10}$ of Benjamini-Hochberg method adjusted p values are shown for MG (**f**) and MΦe (**g**) clusters. **h** Top10 enriched REACTOME terms were derived from MΦe cluster markers. **i** Dotplot depicting the average expression of representative genes, associated to the top enriched terms derived from MΦe cluster markers, for each MC subcluster. Color coded functional annotations are given next to gene names on the y-axis.

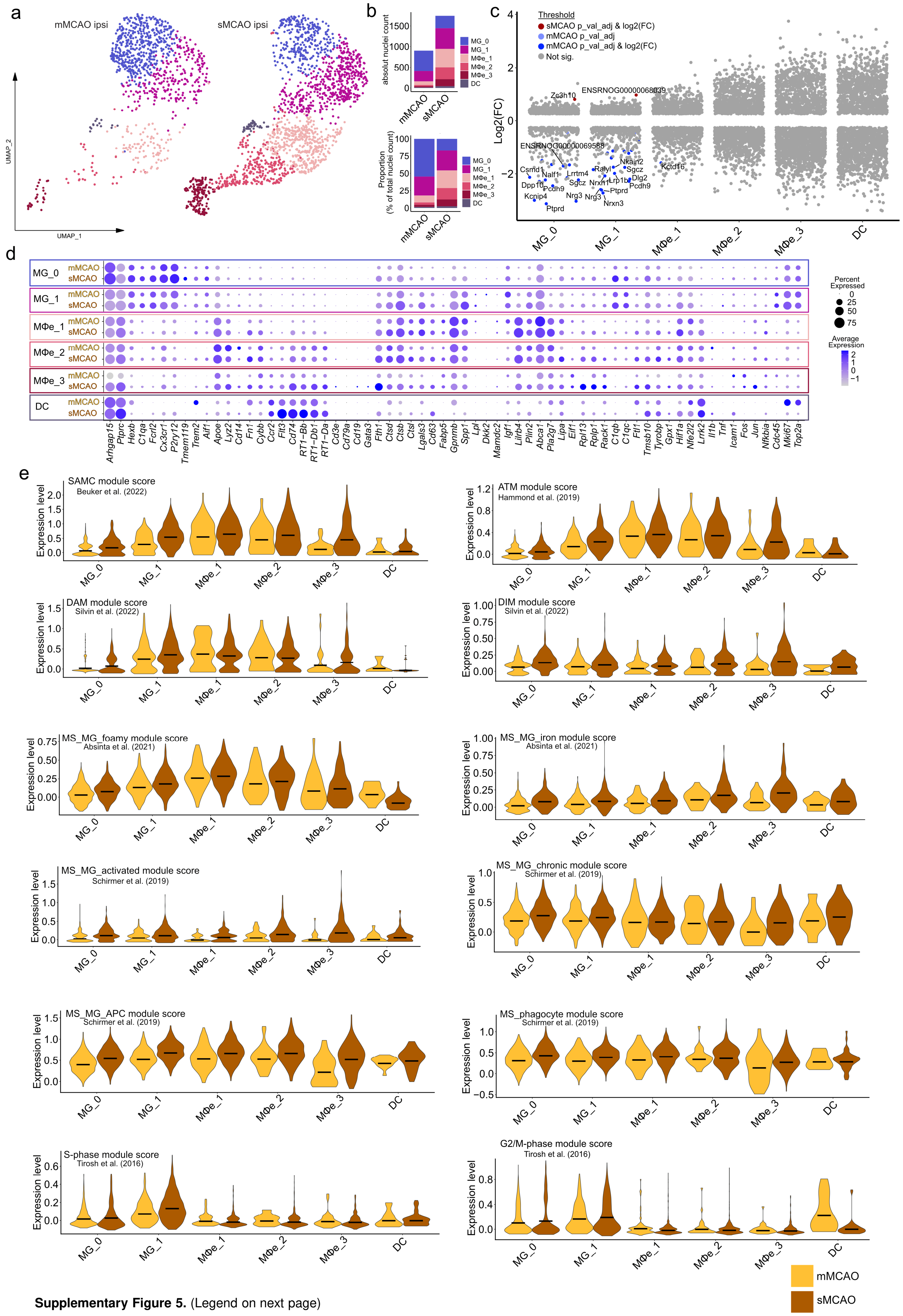

Supplementary Figure 5. (Legend on next page)

Supplementary Figure 5. Comparison of myeloid cell sub clusters derived from moderately and severely infarcted brain tissue. a UMAP plot depicting myeloid cell subclusters (2646 nuclei annotated to 6 sub clusters in total), derived from infarcted tissue (MCAO ipsi), split by stroke severity (moderate= mMCAO ipsi vs severe= sMCAO ipsi). **b** Stacked bar plots depicting the absolute and relative abundance of each cell cluster within each sample. **c** Results of DEG calculations, comparing gene expressions between datasets derived from severe (sMCAO) and moderate infarctions (mMCAO), for each myeloid cell subcluster. DEGs are presented as color coded strip plot, up to top 10 significantly (adjusted p-values <0.05) up- and downregulated DEGs, sorted by log2FC are labelled. **d** Dotplot depicting the expression of canonical microglia, macrophage, DC, MHC-II and lymphoid cell associated genes, as well as representative genes from previously described MC gene sets within each myeloid subcluster (described in Supplementary figure 4), split by stroke severity. **e** Module score violin plots showing aggregate expression levels of various gene sets for each subcluster, split by stroke severity. Abbreviations: MG: microglia, MΦ: macrophage, MΦe: macrophage enriched, DC: dendritic cell, MHC: major histocompatibility complex, SAMC: stroke associated myeloid cell, ATM: Axon Tract-Associated Microglia, DAM: disease-associated microglia, MS_MG: Multiple sclerosis associated microglia, DIM: disease inflammatory macrophage, APC: antigen-presenting cell.

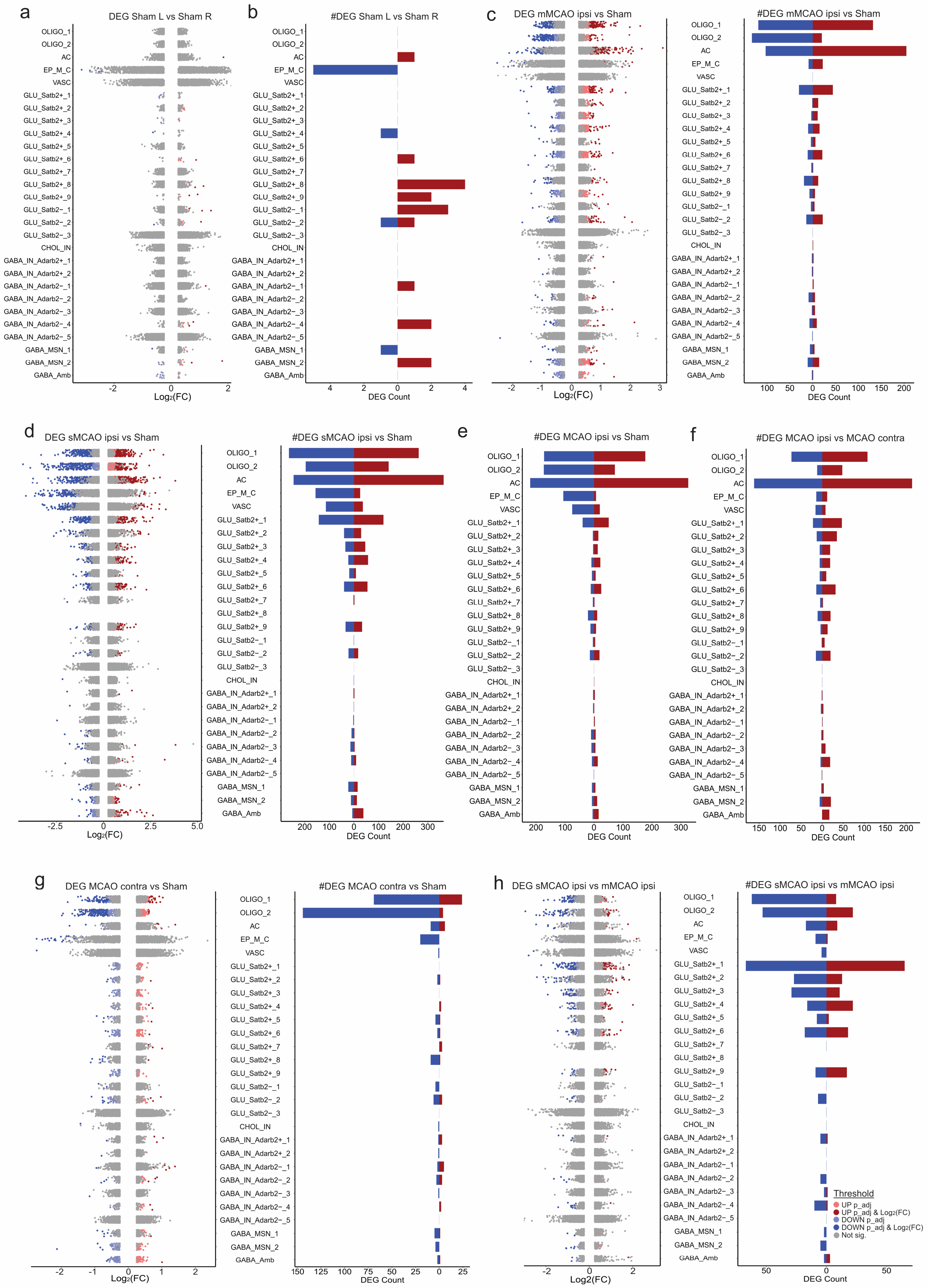

Supplementary Figure 6. (Legend on next page)

Supplementary Figure 6. Global distribution of MCAO induced differentially expressed genes (DEGs) across all major cell clusters. Results of DEG calculations for each conserved cell cluster comparing various groups. Bar plots depict the numbers of up- and downregulated DEGs, meeting cut offs for statistical significance (Bonferroni-adjusted p-values < 0.05) and magnitude of change in gene expression (|log2fold change ≥ 0.6|) (=DEG count). Strip plots depict distribution of DEGs, color coded by DEG cut offs, depicted in the lower right legend within the figure. DEGs were calculated using the MAST statistical framework as specified within the Methods section. Results for the following comparisons are shown: **a,b** DEG distribution (**a**) and DEG counts (**b**) from the comparison of the left (L) and right (R) hemisphere datasets derived from Sham control animals (n=4 animals). **c,d** DEG distribution left, DEG counts right from the comparison of datasets derived from hemispheres with moderate (mMCAO ipsi) (n=3 animals) (**c**) or severe (sMCAO ipsi) (n=4 animals) (**d**) infarction to Sham control datasets (n=4 animals). **e,f** DEG counts derived from the comparison of MCAO infarcted hemisphere datasets (moderate and severe pooled = MCAO ipsi, n=7 animals) to Sham control datasets (Sham, n=4 animals) (**e**) and datasets derived from the hemisphere contralateral to infarction (MCAO contra, n=7 animals) (**f**), corresponding strip plots are shown in figure 1. **g** DEG distribution left, DEG counts right from the comparison of datasets derived from hemispheres contralateral to infarction (MCAO contra, n=7 animals) to Sham datasets (n=4 animals). **h** DEG distribution left, DEG counts right from the comparison of datasets derived from severely infarcted (sMCAO ipsi) and moderately infarcted (mMCAO ipsi) tissue.

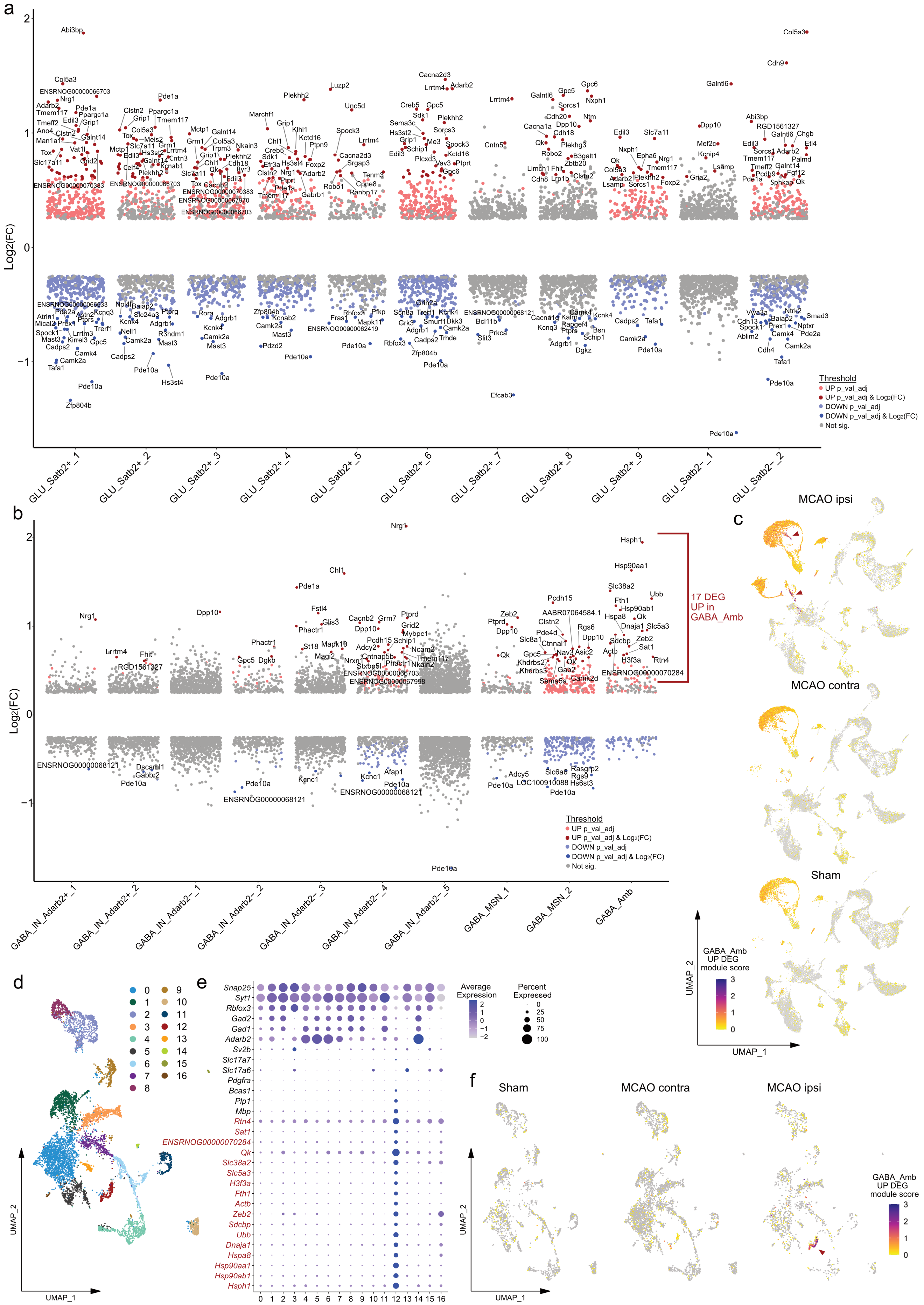

Supplementary Figure 7. (Legend on next page)

Supplementary Figure 7. DEG signatures of neurons within infarcted brain tissue. a,b Results of DEG calculations for glutamatergic (**a**) and GABAergic (**b**) neuronal cell clusters, comparing gene expressions within datasets derived from infarcted tissue (MCAO ipsi) to datasets from the hemisphere contralateral to infarction (MCAO contra) (n=7 animals), for each neuronal cluster. DEGs are presented as color coded strip plots, up to top 20 significantly (adjusted p-values <0.05) up- and downregulated DEGs, sorted by log2FC are labelled. DEGs were calculated using the MAST statistical framework as specified within the Methods section. **c** Module score feature plots depicting the aggregate expression of the 17 DEGs upregulated in the ambiguous GABAergic neuronal cluster (GABA_Amb) in infarcted brain tissue. Feature plots are split by group, red arrowheads highlight UMAP regions with highest module scores, DEGs included in the module score are highlighted in (**b**). **d** UMAP plot depicting unsupervised subclustering analysis of GABA_Amb. **e** Dotplot showing the expression of canonical pan neuronal, GABAergic and glutamatergic neuronal subset markers, as well as oligodendrocyte lineage associated markers and the 17 DEGs upregulated in the GABA_Amb cluster within MCAO ipsi, across all GABA_Amb subclusters. **f** Module score feature plots projecting the aggregate expression of the 17 DEGs upregulated in GABA_Amb in infarcted brain tissue onto the GABA_Amb subclustering UMAP plot, split by treatment group.

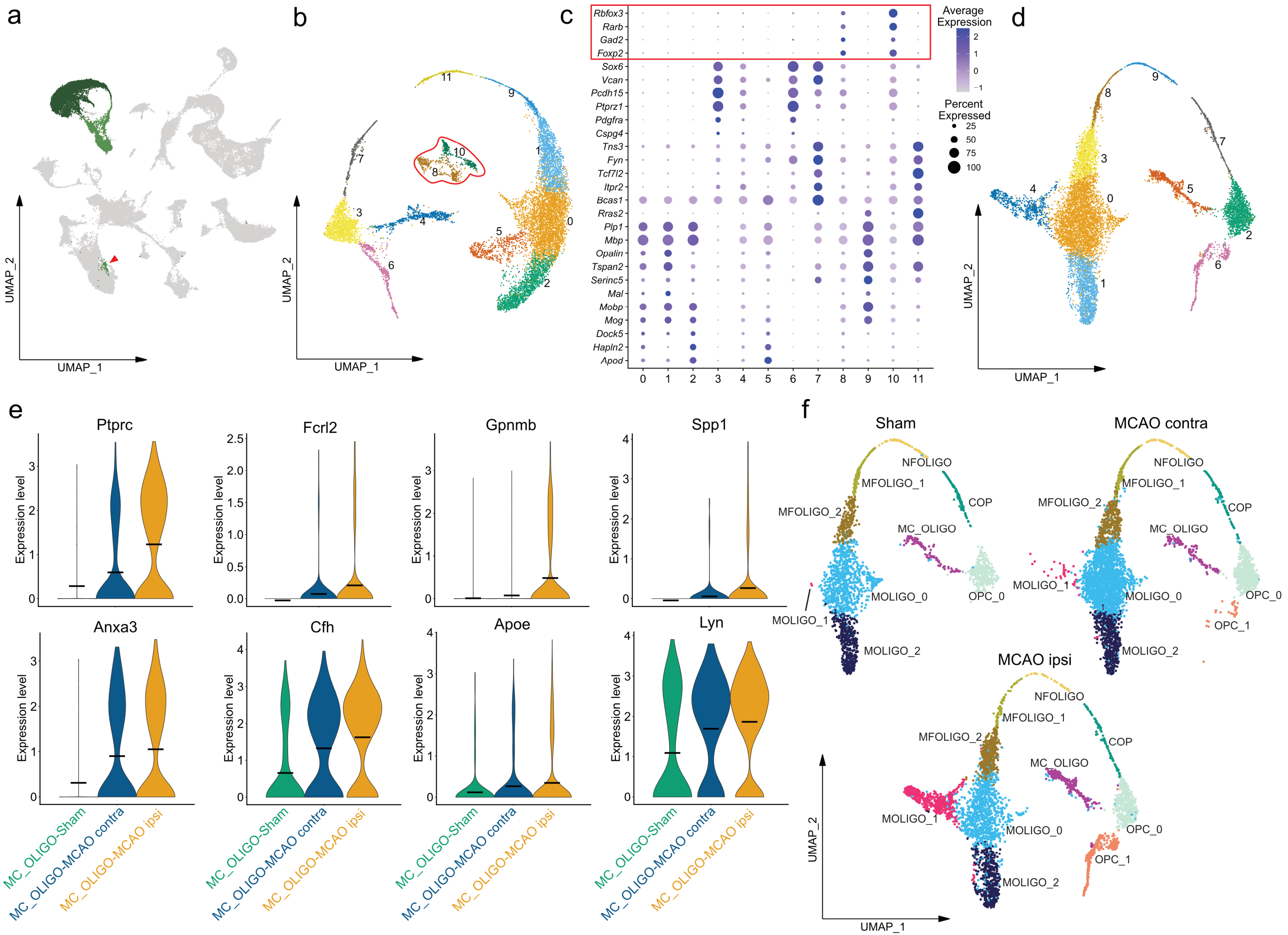

Supplementary Figure 8. Additional pre-processing steps pertaining to Figure 2. a Oligodendrocyte lineage clusters OLIGO_1 and OLIGO_2 are highlighted in the main clustering UMAP plot (green). The red arrowhead points to scattered OLIGO_1 nuclei in proximity to the neuronal cluster MSN_2. **b** UMAP showing unsupervised subclustering of oligodendrocyte lineage clusters, depicting Seurat clusters. **c** Dotplot depicting curated neuronal and oligodendrocyte marker genes. Clusters 8 and 10 and medium spiny neuron (MSN) signature genes specifically expressed within these cluster are highlighted in red. **d** UMAP plot depicting non-annotated oligodendrocyte subclusters, after removal of contaminating subclusters. This subclustering was used for subsequent analyses. **e** Violin plots showing gene expression levels of myeloid cell associated genes within the myeloid cell oligodendrocyte mixed subcluster (MC_OLIGO), split by treatment group. **f** UMAP plots depict distribution of nuclei across subclusters, split by treatment group.

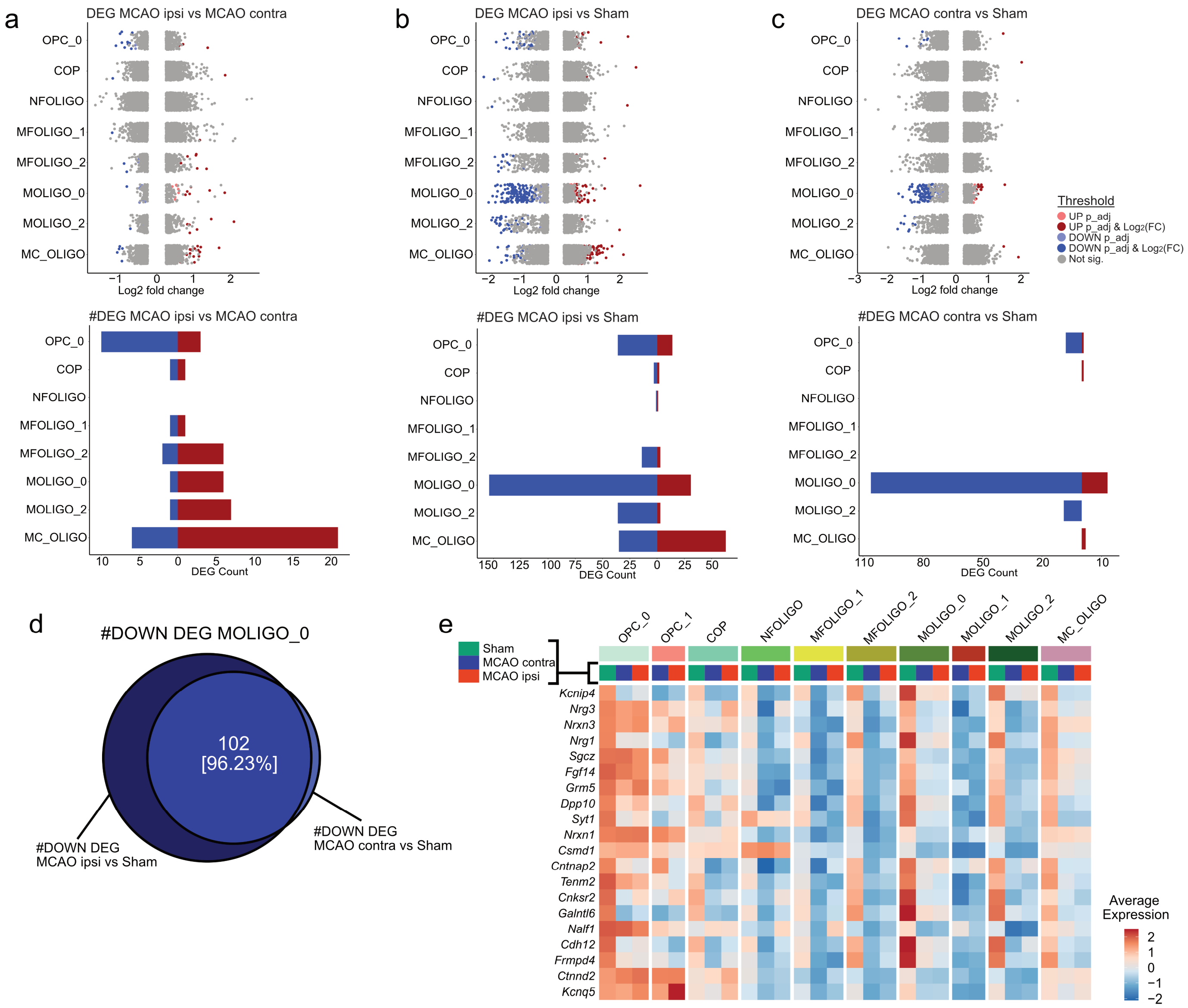

Supplementary Figure 9. Analysis of DEGs in conserved oligodendrocyte lineage sub clusters. Results of DEG calculations for each conserved oligodendrocyte lineage sub cluster, comparing various groups. **a-c** Strip plots (top row) depicting distribution of DEGs, color coded by DEG cut offs and corresponding bar plots (bottom row) depict the numbers of up- and downregulated DEGs, meeting cut offs for statistical significance (Bonferroni-adjusted p-values < 0.05) and magnitude of change in gene expression (|log2fold change ≥ 0.6|) (= DEG count). DEGs were calculated using the MAST statistical framework as specified within the Methods section. Results for the following comparisons are shown: Datasets derived from infarcted tissue (MCAO ipsi) compared to datasets derived from hemispheres contralateral to infarction (MCAO contra), n=7 animals, or Sham controls, n=4 animals (**a** and **b**, respectively) and datasets derived from MCAO contra vs Sham datasets (**c**). **d** Venn diagram depicting the overlap between downregulated DEGs within subcluster MOLIGO_0 in MCAO ipsi relative to Sham (left circle) and MCAO contra relative to Sham (right circle). Numbers denote the absolute and relative numbers of downregulated DEGs derived from the MCAO contra vs Sham comparison, which are also downregulated in MCAO ipsi relative to Sham datasets. **e** Heatmap depicting the average scaled gene expression of the Top 20 DEGs downregulated in MCAO contra relative to Sham, as sorted by log_2fold changes, split by subcluster and group. All of these genes were also significantly downregulated in the MCAO ipsi relative to Sham comparison.

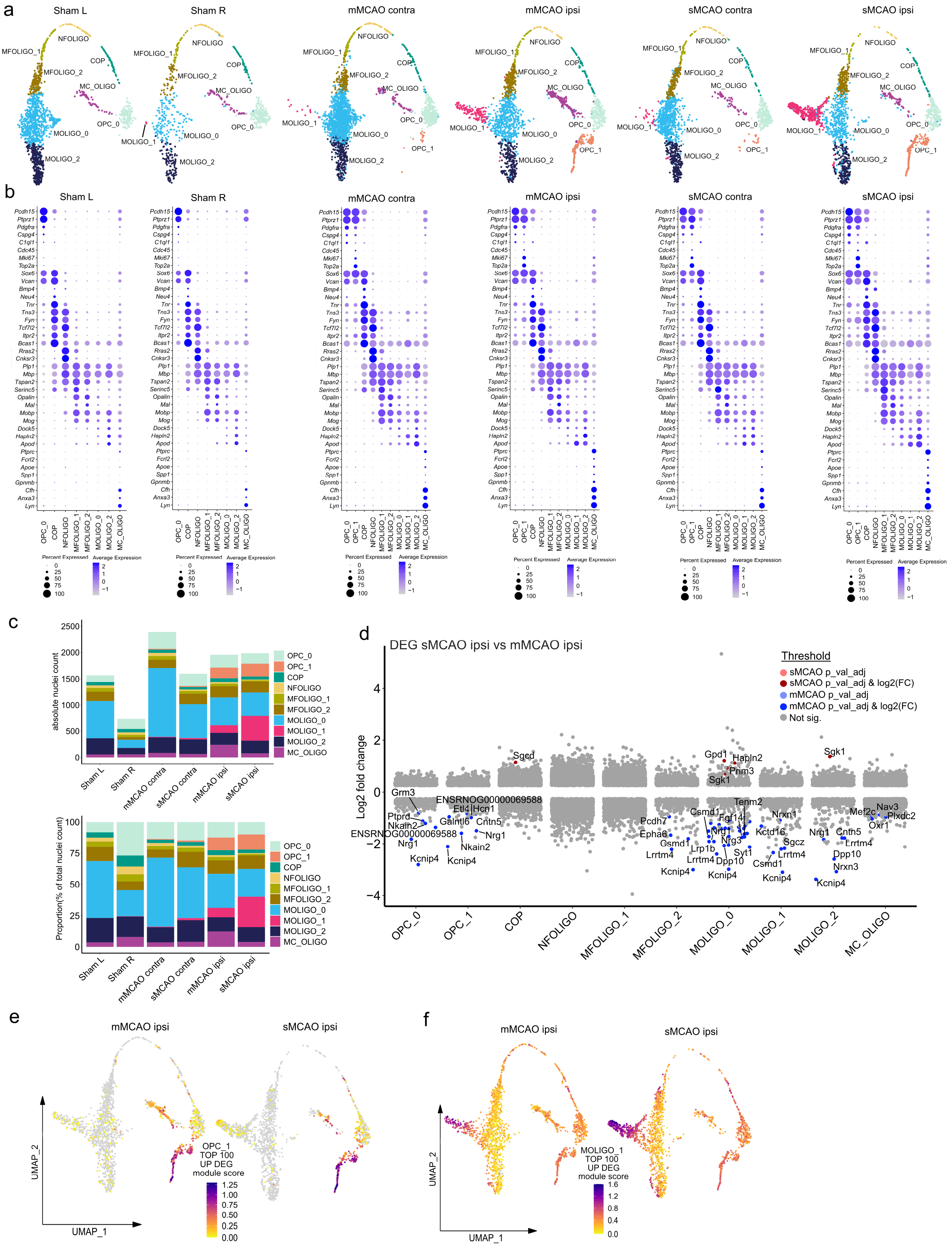

Supplementary Figure 10. (Legend on next page)

Supplementary Figure 10. Nuclei distribution and transcriptional profiles of oligodendrocyte sub clusters, across samples. **a** UMAP plots depicting distribution of nuclei across oligodendrocytes sub clusters, split by individual samples. **b** Dotplots depicting the oligodendrocyte lineage subcluster markers detailed in Fig.2 for all individual samples. **c** Stacked bar plots depicting the absolute and relative abundance of each subcluster within each sample. **d** Results of DEG calculations, comparing gene expressions between datasets derived from severe (sMCAO ipsi) and moderate infarctions (mMCAO ipsi), for each subcluster. DEGs are presented as color coded strip plot, up to top 10 significantly (adjusted p-values <0.05) up- and downregulated DEGs, sorted by log2FC are labelled. **e,f** The top 100 significantly (Bonferroni-adjusted p-values < 0.05) upregulated DEGs, sorted by descending log2fold changes, derived from the comparison of subcluster OPC_1 to OPC_0 (**e**) and MOLIGO_1 to MOLIGO_2 (**f**) were aggregated to module scores and projected onto subcluster UMAP plots, split by infarction severity. DEGs were calculated using the MAST statistical framework as specified within the Methods section.

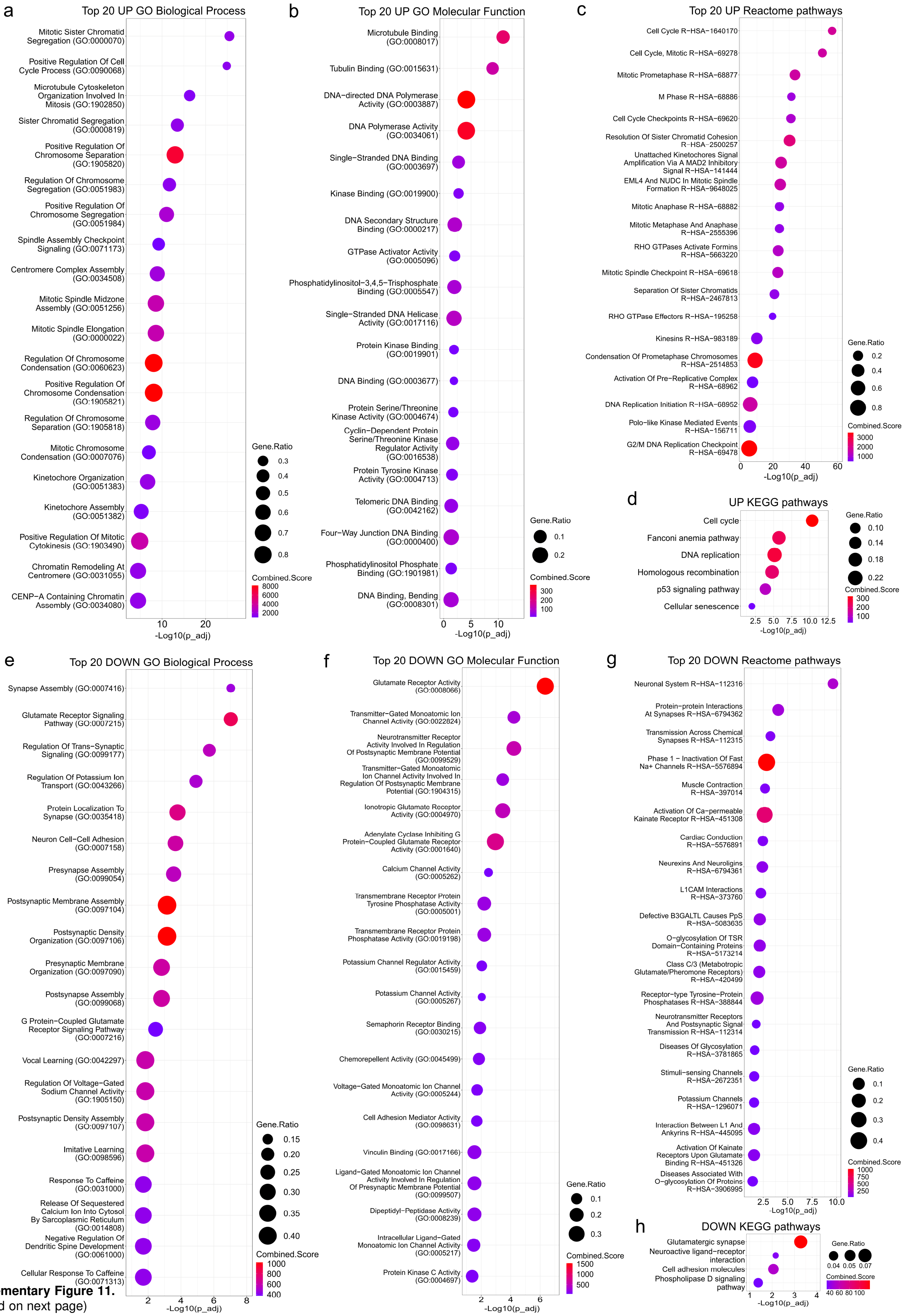

Supplementary Figure 11.
(Legend on next page)

Supplementary Figure 11. Enrichment analysis of OPC_1 marker genes. Results of functional enrichment analyses of DEGs (|log2fold change ≥ 0.6| and Bonferroni-adjusted p-value < 0.05) up- or downregulated in OPC_1 relative to OPC_0. Up to Top 20 enriched terms, subset by combined score and order by –log10 of Benjamini-Hochberg method adjusted p values are depicted as dotplots. **a,b** Enriched GO Biological Process (**a**) and GO Molecular Function (**b**) terms associated to DEGs upregulated in OPC_1. **c,d** Enriched Reactome (**c**) and KEGG (**d**) pathways associated to DEGs upregulated in OPC_1. **e,f** Enriched GO Biological Process (**e**) and GO Molecular Function (**b**) terms associated to DEGs downregulated in OPC_1. **g,h** Enriched Reactome (**g**) and KEGG (**h**) pathways associated to DEGs downregulated in OPC_1.

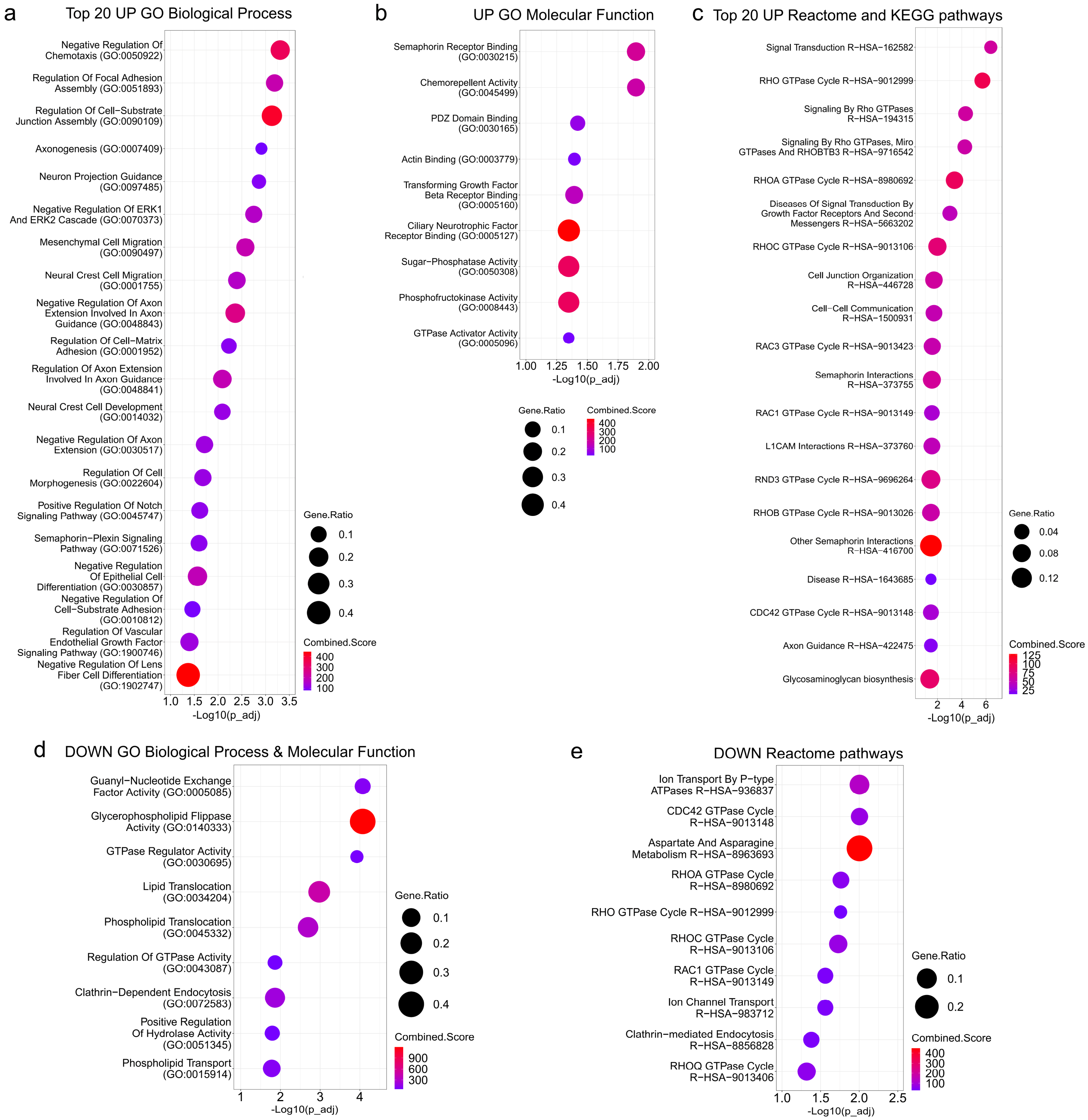

Supplementary Figure 12. Enrichment analysis of MOLIGO_1 marker genes. Results of functional enrichment analyses of DEGs (|log2fold change ≥ 0.6| and Bonferroni-adjusted p-value < 0.05) up- or downregulated in MOLIGO_1 relative to MOLIGO_2. Up to Top 20 enriched terms, subset by combined score and order by –log10 of Benjamini-Hochberg method adjusted p values are depicted as dotplots. **a,b** Enriched GO Biological Process (**a**) and GO Molecular Function (**b**) terms associated to DEGs upregulated in MOLIGO_1. **c** Enriched Reactome and KEGG pathways associated to DEGs upregulated in MOLIGO_1. **d** Enriched GO Biological Process and GO Molecular Function terms associated to DEGs downregulated in MOLIGO_1. **e** Enriched Reactome pathways associated to DEGs downregulated in MOLIGO_1.

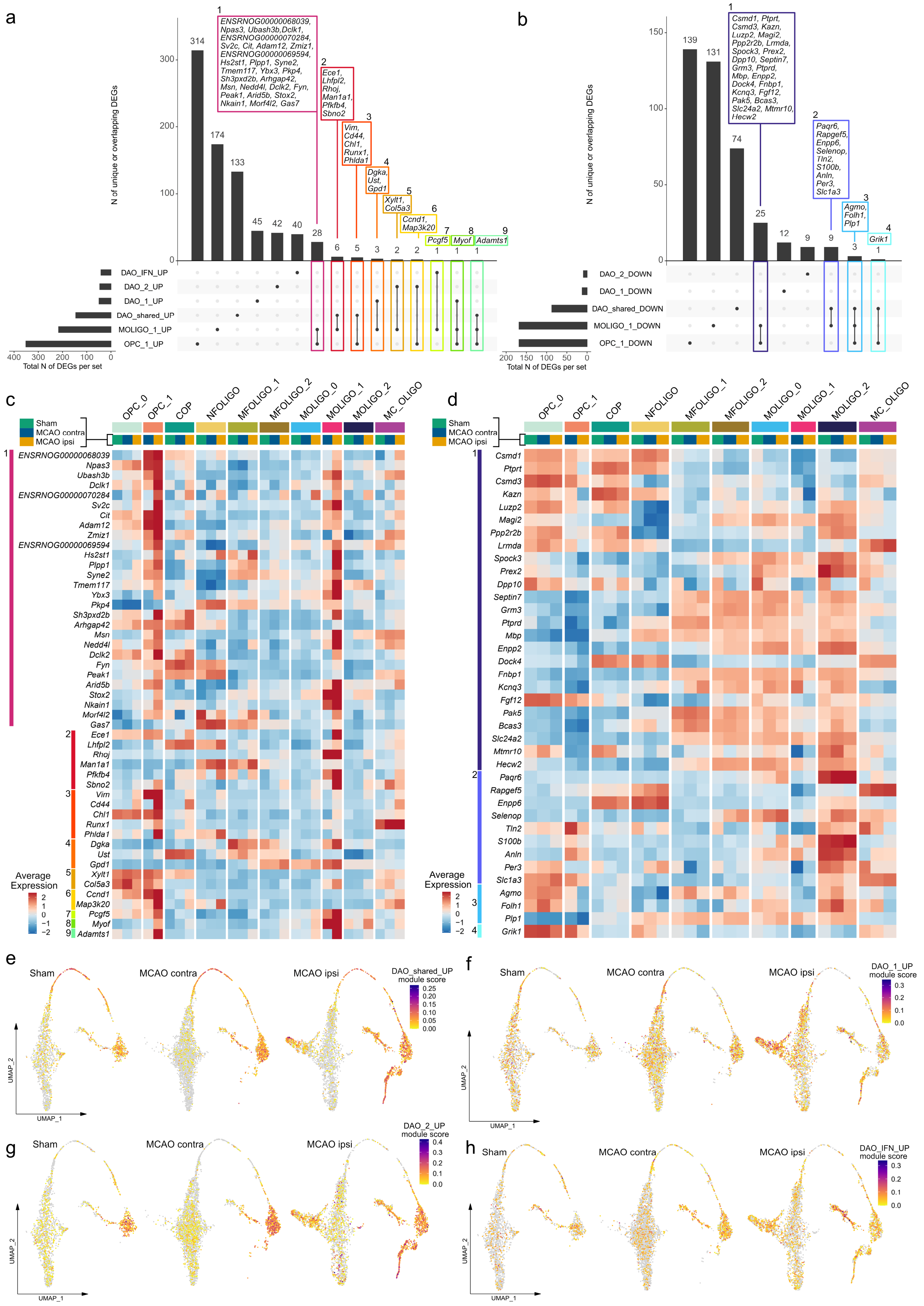

Supplementary Figure 13. (Legend on next page)

Supplementary Figure 13. Limited transcriptional overlap between stroke associated oligodendrocyte lineage cells and diseases associated oligodendrocytes (DAO). a,b UpSet plots depicting the numbers of total, unique and overlapping signature genes of OPC_1, MOLIGO_1 and the DAO signatures: DAO_1 (= immunogenic process associated), DAO_2 (= survival pathway associated) and DAO_IFN (= interferon response associated). **a** UpSet Plot comparing upregulated, **b** UpSet plot comparing downregulated signature genes. For OPC_1 and MOLIGO_1 all up and downregulated DEGs (Bonferroni-adjusted p-values < 0.05 and |log2fold change ≥ 0.6|), derived from the comparison of OPC_1 to OPC_0 and MOLIGO_1 to MOLIGO_2, were included as signature genes, full DAO signature gene sets are given in supplementary data file 1. Overlapping gene stes are named, color coded and numbered sequentially. **c,d** Heatmaps depicting the average scaled gene expression of shared signature genes, derived from the comparisons illustrated in **a,b**, split by subcluster and group, for up- (**c**) and downregulated (**d**) genes. Color codes and numbers on the left side of the gene names (y-axes) correspond to color codes and numbers in **a** and **b**, respectively**. e-h** Module score feature plots projecting aggregate expression of DAO gene sets on to oligodendrocyte subcluster UMAP plots, split by group. **e** Genes upregulated in all DAO sets, **f** genes upregulated in DAO_1, **g** genes upregulated in DAO_2, **h** genes upregulated in DAO_IFN.

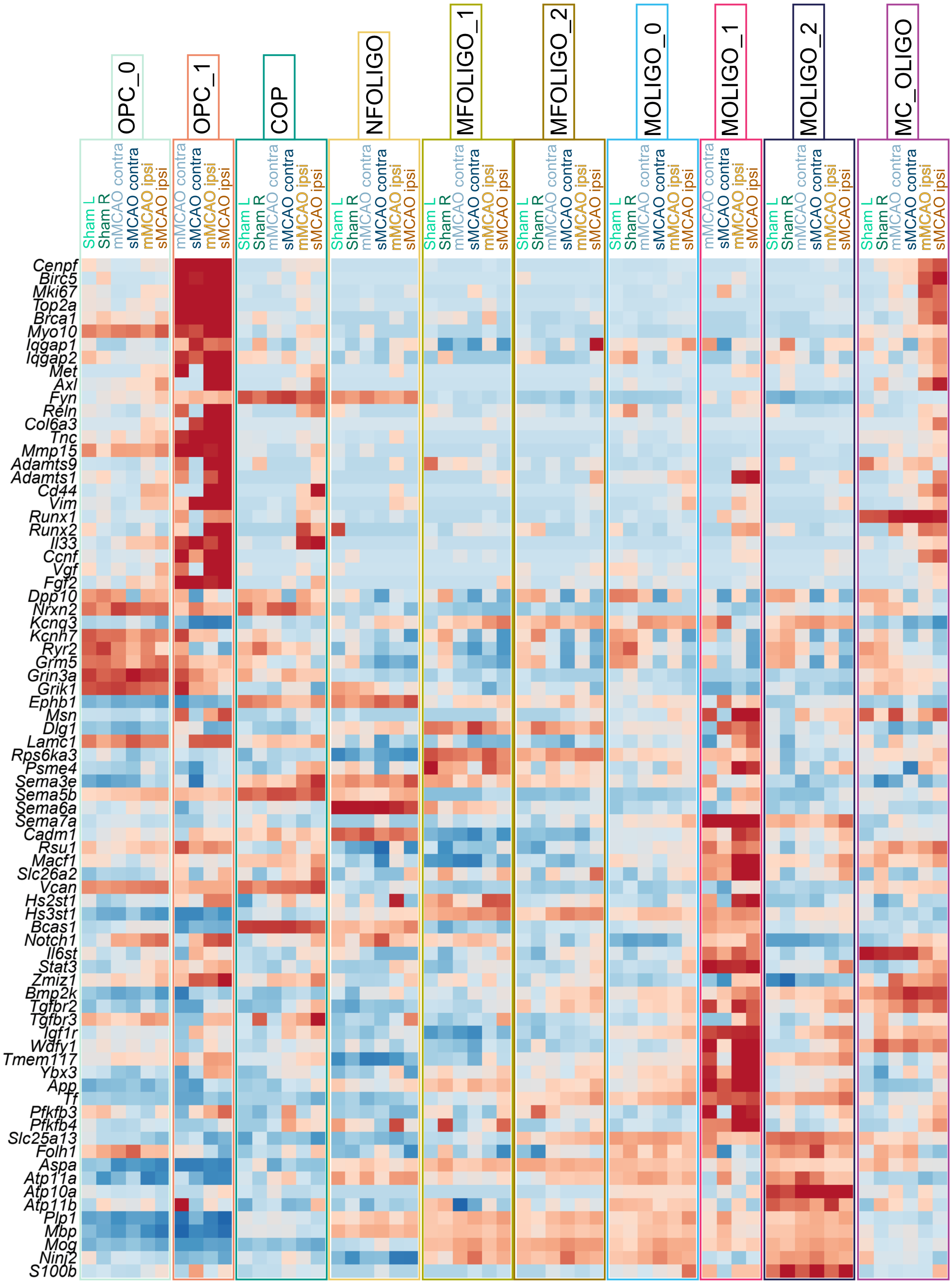

Supplementary Figure 14. Expression of curated OPC_1 and MOLIGO_1 marker DEGs within each sample. Heatmap depicting the average scaled gene expression of curated DEGs shown in Fig.2, split by subcluster and sample.

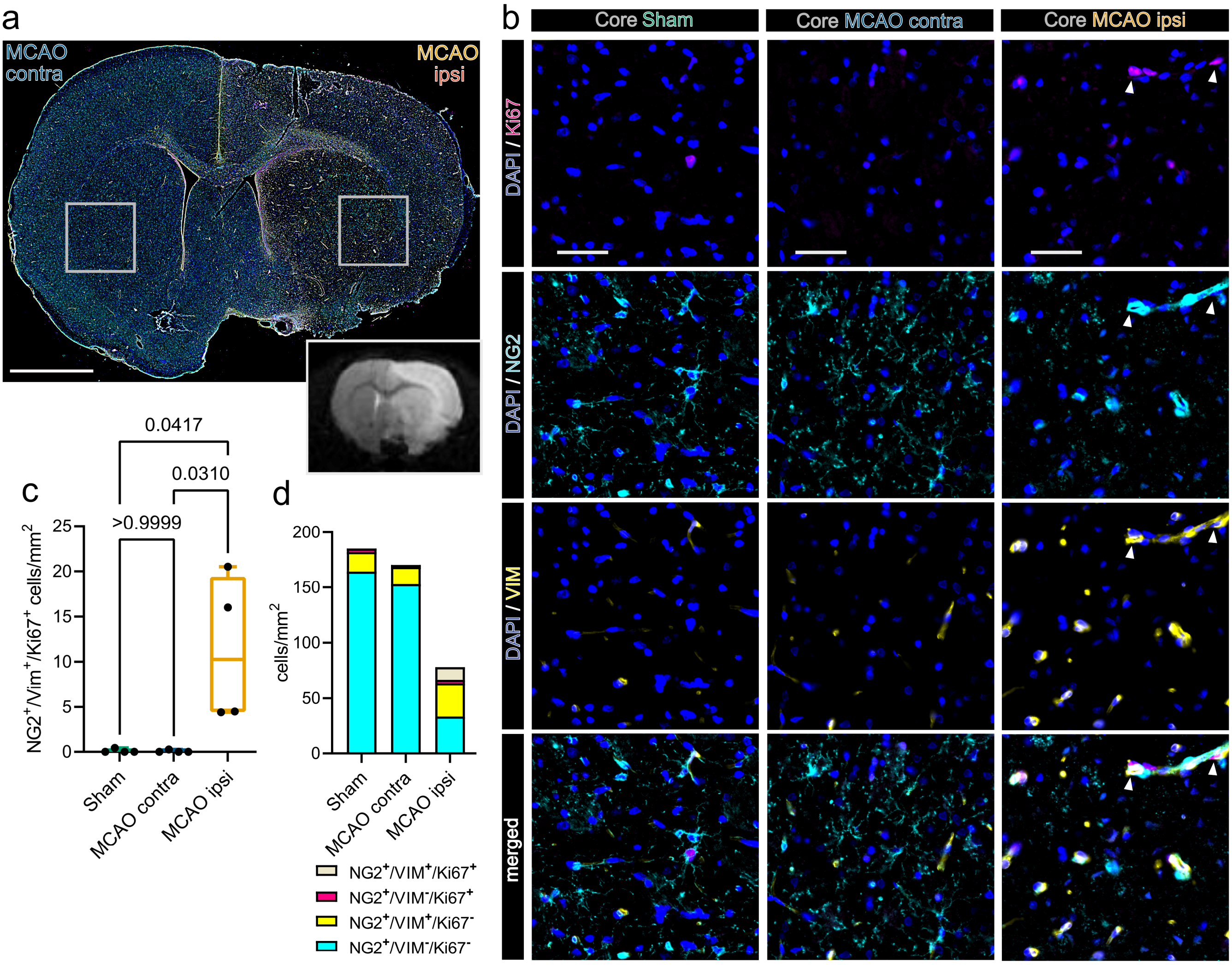

Supplementary Figure 15. Proliferating, VIM positive OPCs are found in the lesion core 48h after filament based permanent MCAO in rats. a Overview of a representative coronal rat brain section 48 h post middle cerebral artery occlusion (MCAO), stained for NG2, VIM and Ki67. Grey boxes highlight lesion core regions of interest (ROIs) within infarcted tissue (MCAO ipsi) and the anatomically corresponding ROI in the hemisphere contralateral to infarction (MCAO contra), lower right inset depicts a corresponding T2 weighted MRI image from the same animal. Bar = 2 mm. **b** Representative images from the lesion core ROI (MCAO ipsi), corresponding contralateral ROI (MCAO contra) and corresponding ROI in Sham operated rats (Sham), split by antigen. Ki67 = magenta, NG2 = Cyan, VIM = yellow, DAPI (nuclei) = blue, bars = 50 µm. White arrowheads point to triple positive cells. **c** Cell counts within lesion core and corresponding ROIs are presented as box plots for $NG2^+/VIM^+/Ki67^+$ triple positive cells. Box plots depict medians, 25th to 75th percentiles as hinges, minimal and maximal values as whiskers, and individual counts, for each animal as dots.**d** Cell counts for $NG2^+/VIM^+/Ki67^+$, $NG2^+/VIM^-/Ki67^+$, $NG2^+/VIM^+/Ki67^-$, $NG2^+/VIM^-/Ki67^-$ are jointly shown as colored stacked bar plot. Data derived from n = 4 animals per group, p values derived from Kruskal-Wallis-H-Tests, followed by Dunn's post hoc comparisons. Statistical comparison of all subsets within all ROIs is reported in Supplementary Data 7. Source data are provided with this paper.

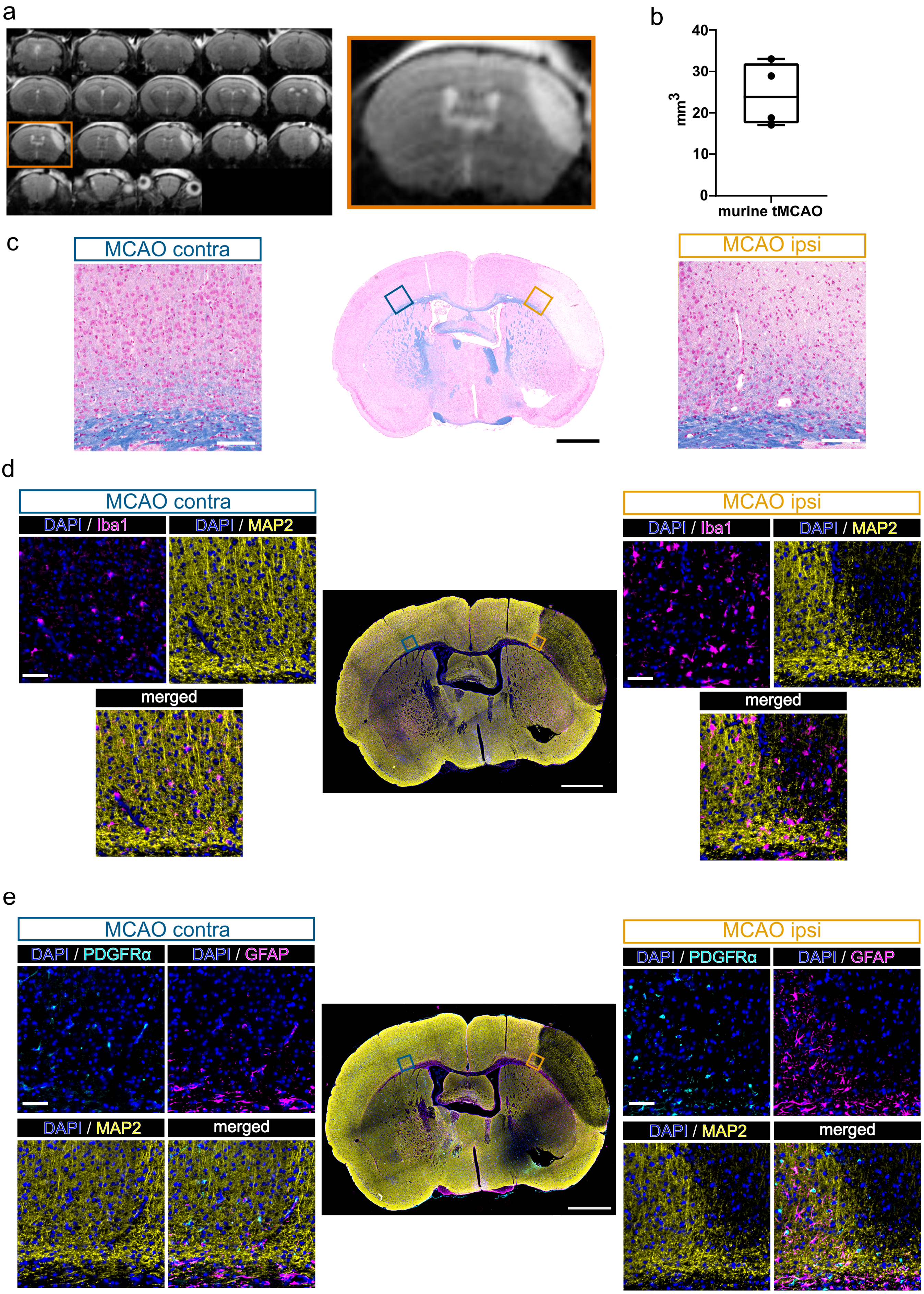

Supplementary Figure 16. (Legend on next page)

Supplementary Figure 16. MRI validation of cerebral infarction and histological characterization of ischemic lesions 48h after thromboembolic MCAO in mice. a T2-weighted MRI image series from a middle cerebral artery occlusion (MCAO) lesioned mouse brain. Histological and immunofluorescence based stainings in **c-e** were performed on a coronal brain section from the same mouse and correspond to the MRI section highlighted in orange. **b** Distribution of overall infarct lesion sizes in mice 48h after thromboembolic MCAO are presented as box plot, depicting medians, 25th to 75th percentiles as hinges, minimal and maximal values as whiskers, and individual lesion volumes for each animal as dots (n=4). **c** Luxol Fast Blue staining of a coronal section from a murine brain 48h post MCAO, visualizing myelin loss. Close up images depict the perilesional area at the grey-white matter border (MCAO ipsi), as well as the anatomically corresponding region of the hemisphere contralateral to infarction (MCAO contra). Scale bars: Black scale bar in overview image: 1mm, white scale bars in close up images: 100µm. **d,e** Immunofluoresence based colocalization of MAP2 (yellow) and Iba1 (magenta) (**d**), as well as MAP2 (yellow), GFAP (magenta) and PDGFRα (cyan) (**e**) in a coronal section from a murine brain 48h post MCAO, visualizing neuronal loss and presence of myeloid and glial cells at the lesion border. Close up images depict the perilesional area at the grey-white matter border (MCAO ipsi), as well as the anatomically corresponding region of the hemisphere contralateral to infarction (MCAO contra). Scale bars: Scale bars in overview images: 1mm, Scale bars in close up images: 50µm.

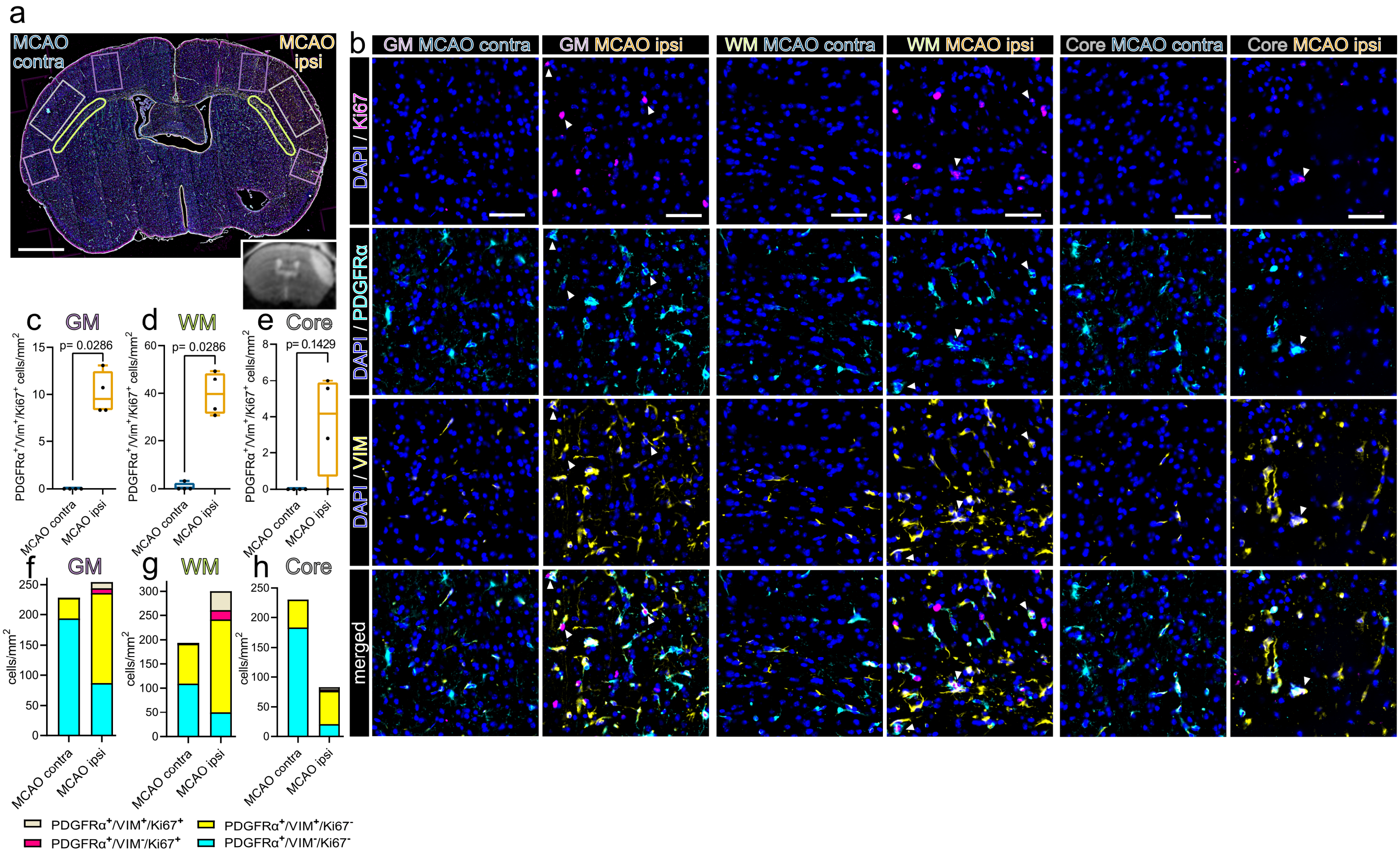

Supplementary Figure 17. Proliferating, VIM positive OPCs accumulate in the perilesional zone 48h after thromboembolic MCAO in mice. a Overview of a representative coronal mouse brain section 48 h post MCAO, stained for PDGFRα, VIM and Ki67. Grey matter regions of interest (ROIs) (GM) are highlighted in violet, white matter ROIs (WM) in lime green and the lesion core and its corresponding region in the contralateral hemisphere are highlighted in grey. Lower right inset depicts a corresponding T2 weighted MRI image from the same animal. Bar = 1 mm. **b** Representative images from GM, WM and core ROIs within the infarcted (MCAO ipsi) and contralateral (MCAO contra) hemisphere, split by antigen. Ki67 = magenta, PDGFRα = Cyan, VIM = yellow, DAPI (nuclei) = blue, bars = 50 µm. White arrowheads point to triple positive cells. **c-e** Cell counts within GM (**c**), WM (**d**) and Core (**e**) ROIs respectively are presented as box plots for PDGFRα$^+$/VIM$^+$/Ki67$^+$ triple positive cells. Box plots depict medians, 25th to 75th percentiles as hinges, minimal and maximal values as whiskers, and individual counts, for each animal as dots. Cell counts for PDGFRα$^+$/VIM$^+$/Ki67$^+$, PDGFRα$^+$/VIM$^-$/Ki67$^+$, PDGFRα$^+$/VIM$^+$/Ki67$^-$ and PDGFRα$^+$/VIM$^-$/Ki67$^-$ cell counts are also jointly shown as colored stacked bar plot. Data derived from n = 4 animals per group, p values derived from Mann-Whitney-U-tests. Statistical comparison of all subsets within all ROIs is reported in Supplementary Data 8. Source data are provided with this paper.

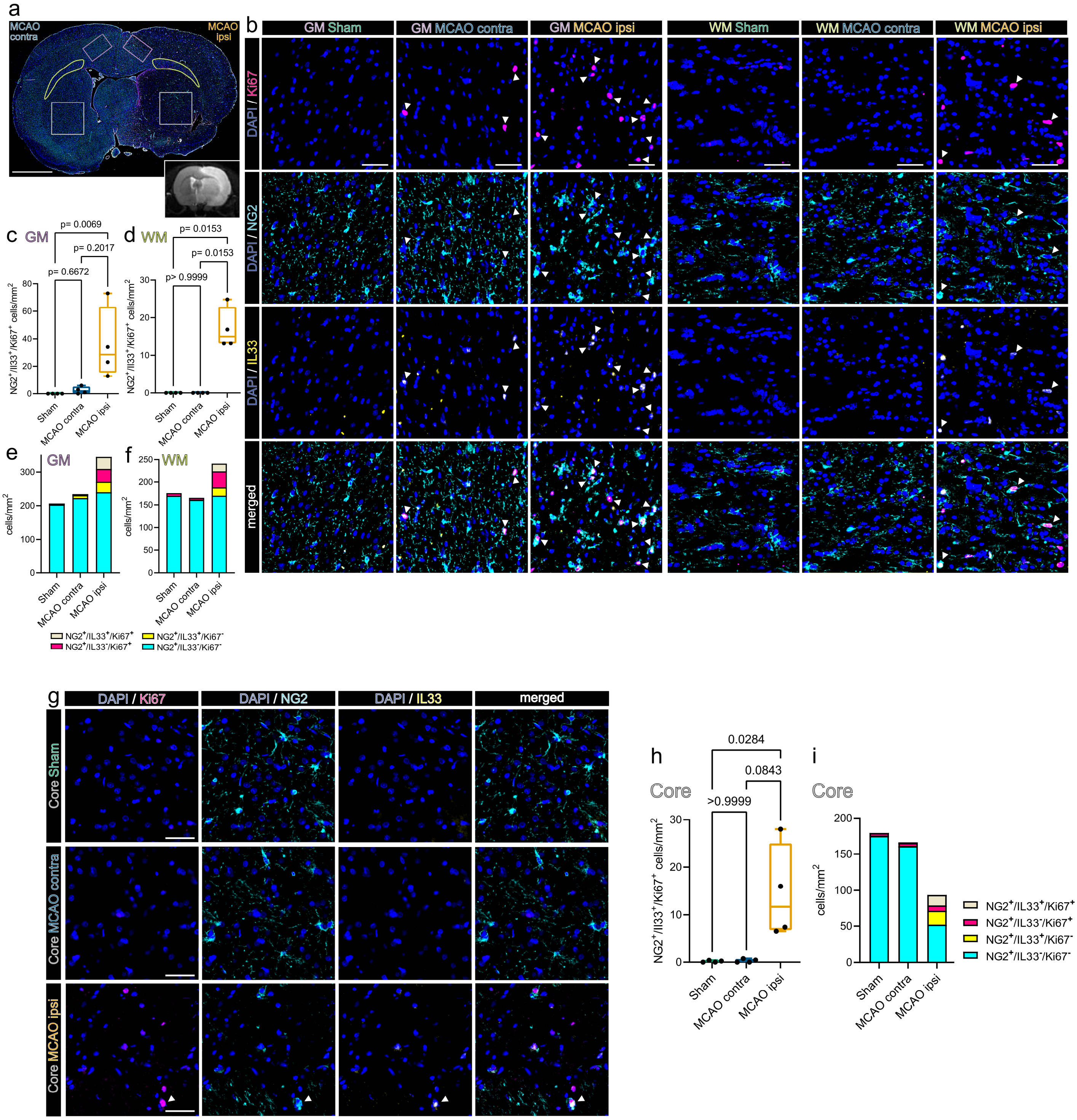

Supplementary Figure 18. Proliferating, IL33 positive OPCs are found in the perilesional grey matter, affected white matter and lesion core 48h after filament based permanent MCAO in rats. a Representative coronal overview, 48 h post middle cerebral artery occlusion (MCAO), stained for NG2, IL33 and Ki67. Grey matter regions of interest (ROIs) (GM) are highlighted in violet, white matter ROIs (WM) in lime green and the lesion core ROI and its corresponding ROI in the contralateral hemisphere are highlighted in grey. Lower right inset shows a corresponding MRI image from the same animal. Bar = 2 mm. **b** Representative images from GM and WM ROIs derived from Sham, MCAO contra and MCAO ipsi groups, split by antigen. Ki67 = magenta, NG2 = Cyan, IL33 = yellow, DAPI (nuclei) = blue, bars = 50 µm- White arrowheads point to triple positive cells. **c,d** Cell counts within GM (**c**) and WM (**d**) are presented as box plots for $NG2^+/IL33^+/Ki67^+$ triple positive cells. Cell counts for $NG2^+/IL33^+/Ki67^+$, $NG2^+/IL33^-/Ki67^+$, $NG2^+/IL33^+/Ki67^-$, $NG2^+/IL33^-/Ki67^-$ are jointly shown as colored stacked bar plot, for GM (**e**) and WM (**f**) ROIs. **g** Representative images from lesion core and anatomically corresponding ROIs derived from Sham, MCAOcontra and MCAOipsi groups, split by antigen. Ki67 = magenta, NG2 = Cyan, IL33 = yellow, DAPI (nuclei) = blue, bars = 50 µm. White arrowhead points to a triple positive cells. **h** Cell counts within lesion core and anatomically corresponding ROIs derived from Sham and MCAO contra are presented as box plots for $NG2^+/IL33^+/Ki67^+$ triple positive cells. **i** Cell counts for $NG2^+/IL33^+/Ki67^+$, $NG2^+/IL33^-/Ki67^+$, $NG2^+/IL33^+/Ki67^-$, $NG2^+/IL33^-/Ki67^-$ are jointly shown as colored stacked bar plot. All box plots depict medians, 25th to 75th percentiles as hinges, minimal and maximal values as whiskers, and individual counts, for each animal as dots. Data derived from n = 4 animals per group, p values derived from Kruskal-Wallis-H-Tests, followed by Dunn's post hoc comparisons. Statistical comparison of all subsets within all ROIs is reported in Supplementary Data 7. Source data are provided with this paper.

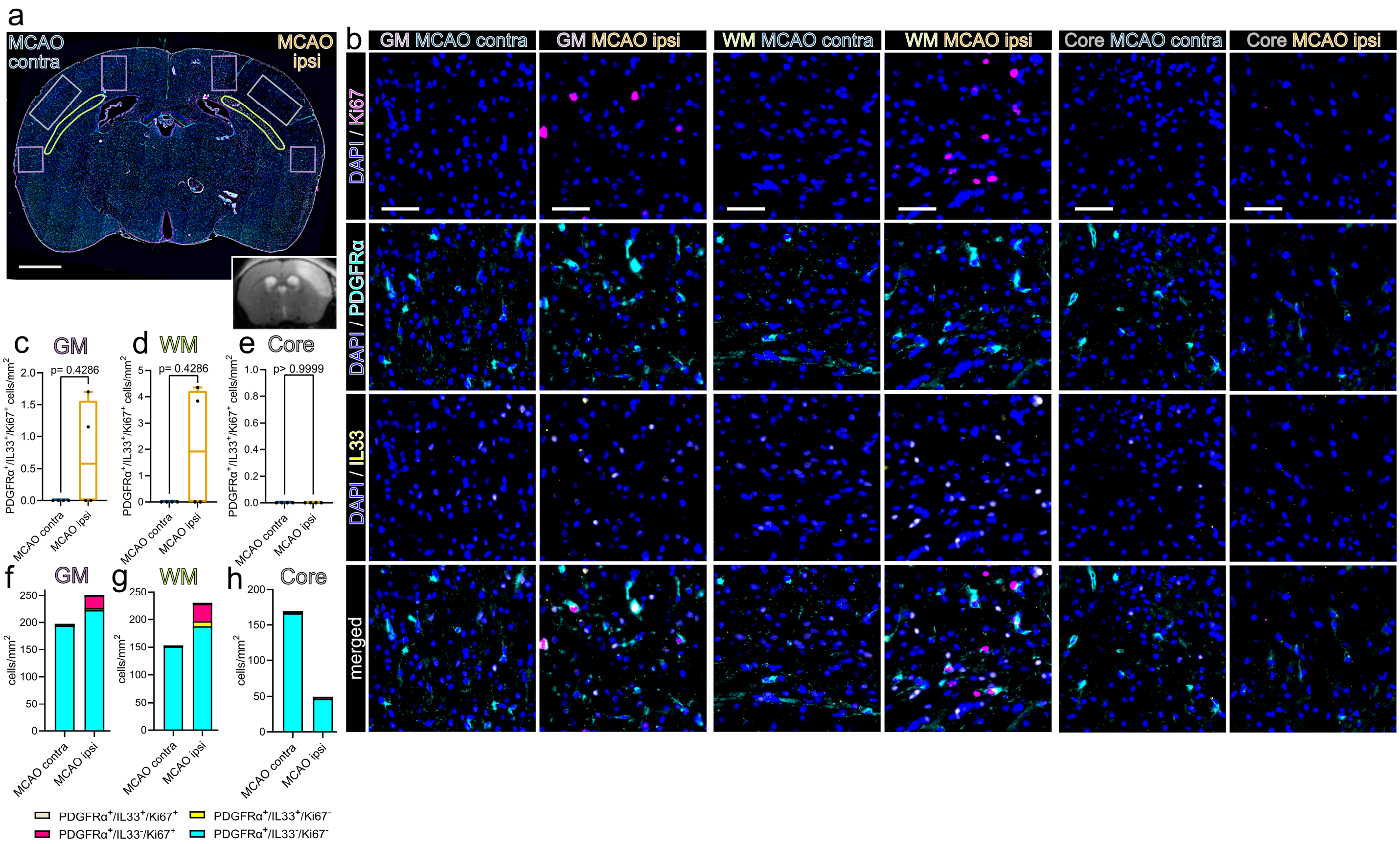

Supplementary Figure 19. Proliferating OPCs do not upregulate IL33 within infarcted hemispheres 48h after thromboembolic MCAO in mice. a Overview of a representative coronal mouse brain section 48 h post MCAO, stained for PDGFRα, IL33 and Ki67. Grey matter regions of interest (ROIs) (GM) are highlighted in violet, white matter ROIs (WM) in lime green and the lesion core and its corresponding region in the contralateral hemisphere are highlighted in grey. Lower right inset depicts a corresponding T2 weighted MRI image from the same animal. Bar = 1 mm. **b** Representative images from GM, WM and core ROIs within the infarcted (MCAO ipsi) and contralateral (MCAO contra) hemisphere, split by antigen. Ki67 = magenta, PDGFRα = Cyan, IL33 = yellow, DAPI (nuclei) = blue, bars = 50 µm. **c-e** Cell counts within GM (**c**), WM (**d**) and Core (**e**) ROIs respectively are presented as box plots for PDGFRα$^+$/IL33$^+$/Ki67$^+$ triple positive cells. Box plots depict medians, 25th to 75th percentiles as hinges, minimal and maximal values as whiskers, and individual counts, for each animal as dots. **f-h** Cell counts for PDGFRα$^+$/IL33$^+$/Ki67$^+$, PDGFRα$^+$/IL33$^-$/Ki67$^+$, PDGFRα$^+$/IL33$^+$/Ki67$^-$ and PDGFRα$^+$/IL33$^-$/Ki67$^-$ cell counts are jointly shown as colored stacked bar plot, for GM (**f**), WM (**g**) and core (**h**) ROIs. Data derived from n = 4 animals per group, p values derived from Mann-Whitney-U-tests. Statistical comparison of all subsets within all ROIs is reported in Supplementary Data 8. Source data are provided with this paper.

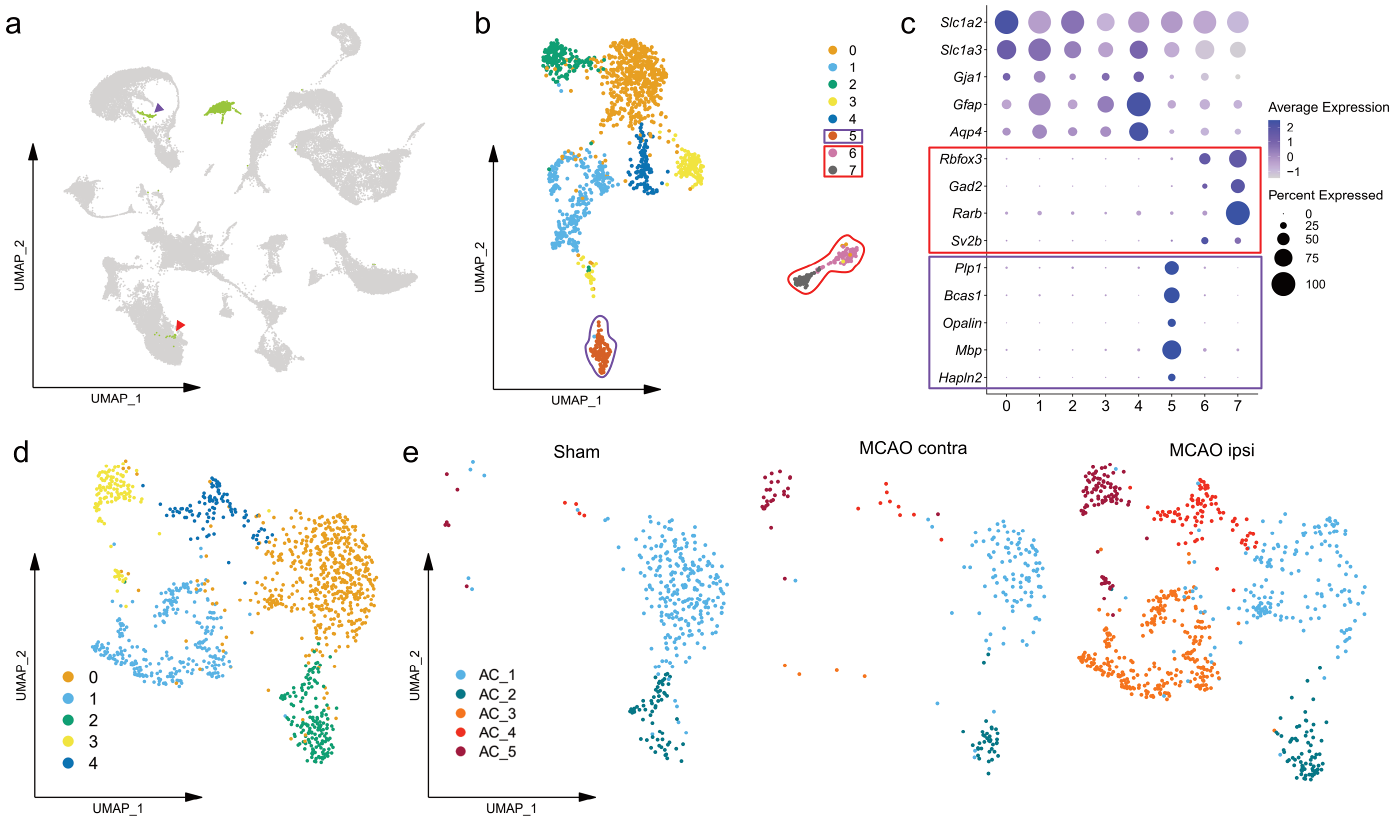

Supplementary Figure 20. Additional pre-processing steps pertaining to Figure 4. a-d Removal of neuronal and oligodendrocyte transcript contaminated subclusters. **a** Astrocyte cluster (AC) highlighted in the main UMAP plot (green). The arrowheads point to scattered AC nuclei in proximity to oligodendrocyte clusters and the neuronal cluster MSN_2. **b** UMAP plot showing non-annotated unsupervised subclustering of AC, with color coded Seurat clusters. **c** Corresponding dotplot, depicting curated astrocyte, neuronal and oligodendrocyte marker genes. Clusters contaminated with neuronal transcripts (6 and 7) and corresponding neuronal markers are highlighted in red in **b** and **c**. Cluster 5 contaminated with oligodendrocyte transcripts and corresponding oligodendrocyte marker genes are highlighted in violet in **b** and **c**. **d** UMAP plot showing non-annotated astrocyte subclusters, after removal of contaminating subclusters. **e** UMAP plots depict distribution of nuclei across subclusters, split by treatment group.

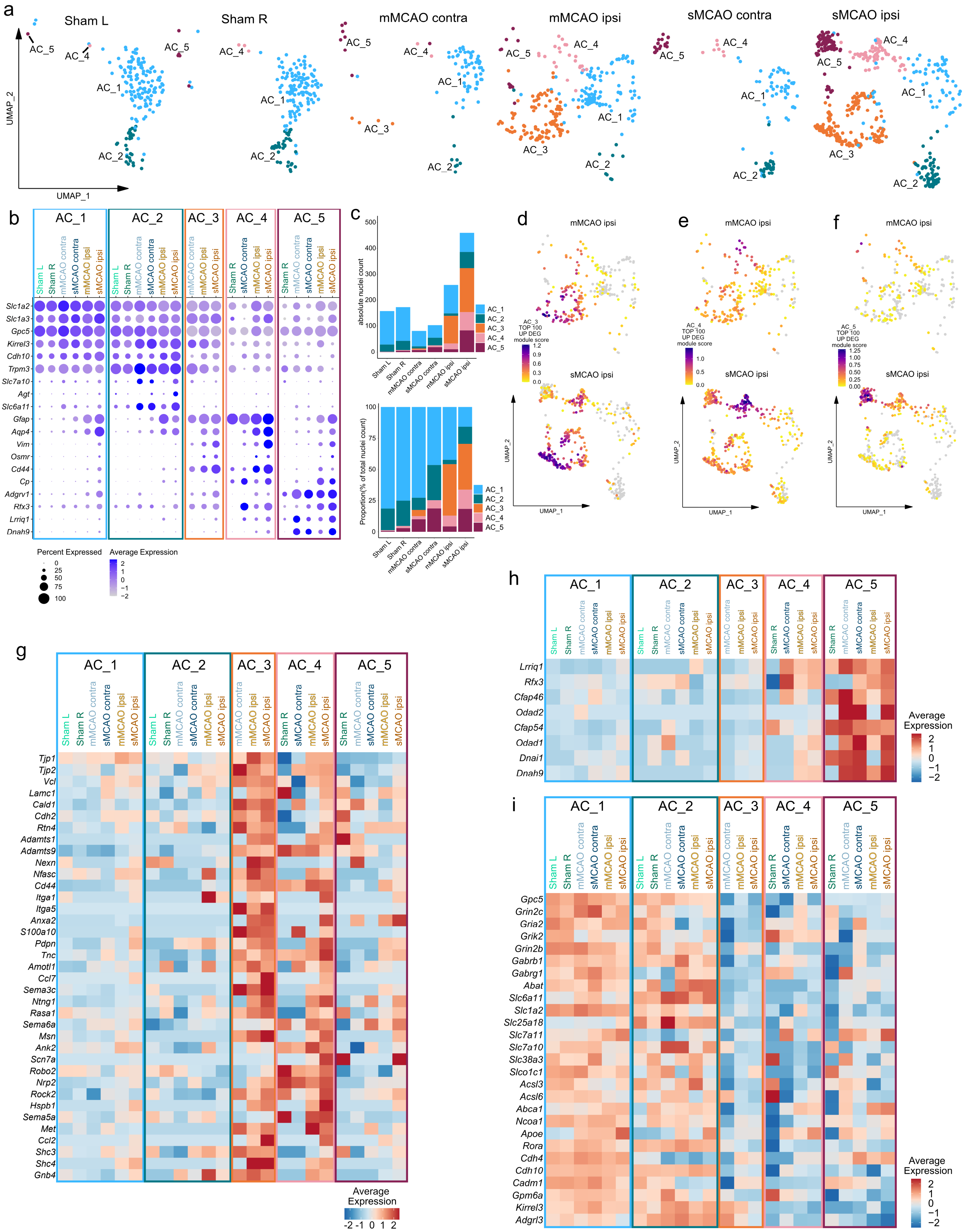

Supplementary Figure 21. (Legend on next page)

Supplementary Figure 21. Nuclei distribution and transcriptional profiles of astrocyte subclusters across samples. **a** UMAP plots depict distribution of nuclei across astrocyte subclusters, split by individual samples. **b** Dotplots depicting homeostatic and reactive subcluster markers detailed in Fig.4, for all individual samples. **c** Stacked bar plots depicting the absolute and relative abundance of each sub cluster within each sample. **d-f** The top 100 significantly (Bonferroni-adjusted p-values < 0.05) upregulated DEGs, sorted by descending log2fold changes, derived from the comparison of each reactive astrocyte subcluster, AC_3 (**d**), AC_4 (**e**) and AC_5 (**f**) to the homeostatic astrocyte subclusters (AC_1, AC_2 pooled) were aggregated to module scores and projected onto subcluster UMAP plots, split by infarction severity (mMCAO ipsi = moderate, sMCAO ipsi = severe infarctions). **g-i** Heatmaps depicting the average scaled gene expression of curated upregulated DEGs, derived from the comparison of AC_3 and AC_4 (**g**) and AC_5 (**h**) to homeostatic astrocytes, as well as DEGs downregulated in reactive astrocytes (**i**), split by subclusters and samples.

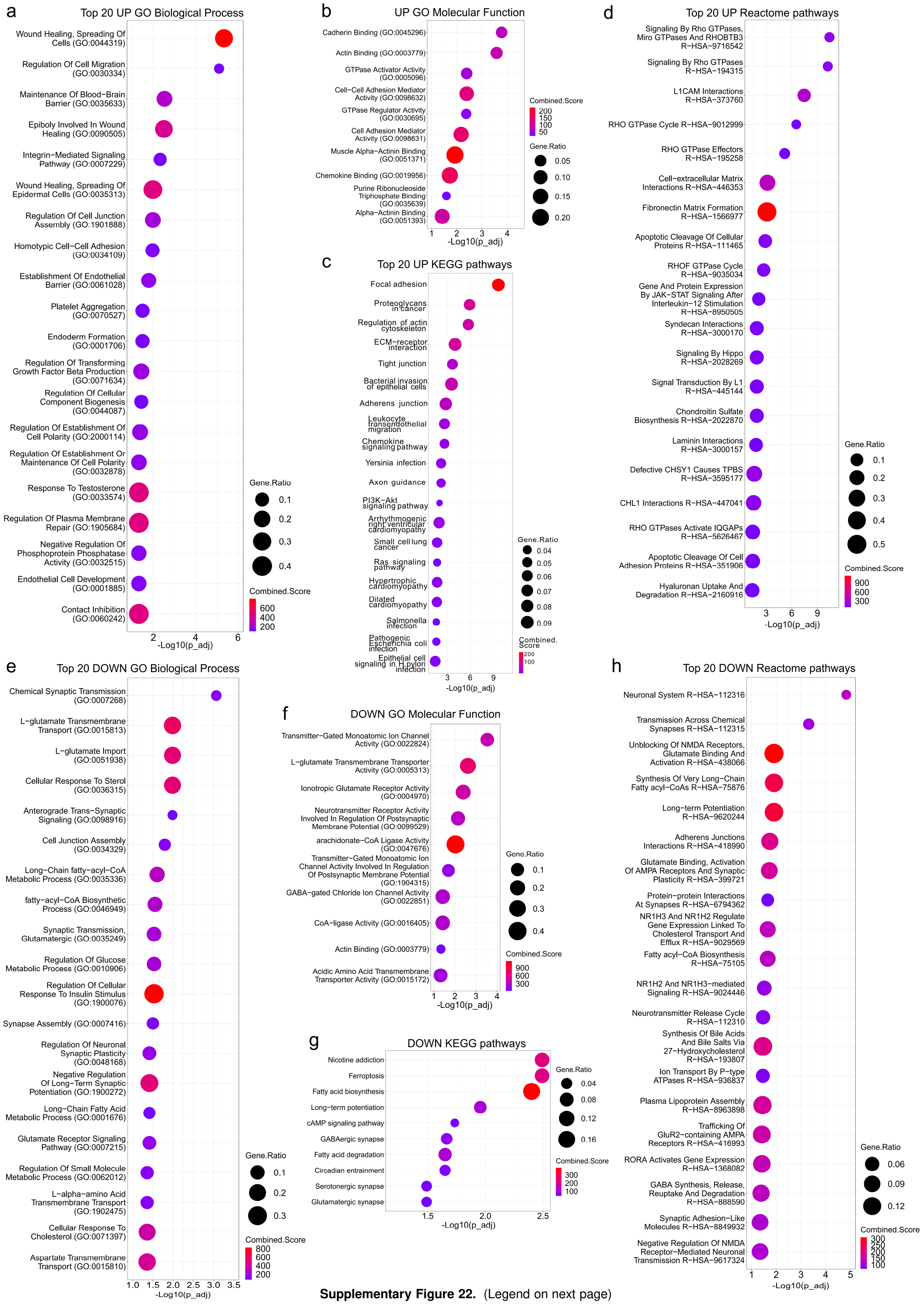

Supplementary Figure 22. (Legend on next page)

Supplementary Figure 22. Enrichment analysis of AC_3 marker genes. Results of functional enrichment analyses of DEGs (|log2fold change ≥ 0.6| and Bonferroni-adjusted p-value < 0.05) up- or downregulated in reactive astrocyte sub cluster AC_3 relative to homeostatic astrocytes (AC_1 and AC_2, pooled). Up to Top 20 enriched terms, subset by combined score and order by –log10 of Benjamini-Hochberg method adjusted p values are depicted as dotplots. **a,b** Enriched GO Biological Process (**a**) and GO Molecular Function (**b**) terms associated to DEGs upregulated in AC_3. **c,d** Enriched KEGG **(c)** and Reactome **(d)** pathways associated to DEGs upregulated in AC_3. **e,f** Enriched GO Biological Process (**e**) and GO Molecular Function (**f**) terms associated to DEGs downregulated in AC_3. **g,h** Enriched KEGG (**g**) and Reactome (**h**) pathways associated to DEGs downregulated in AC_3.

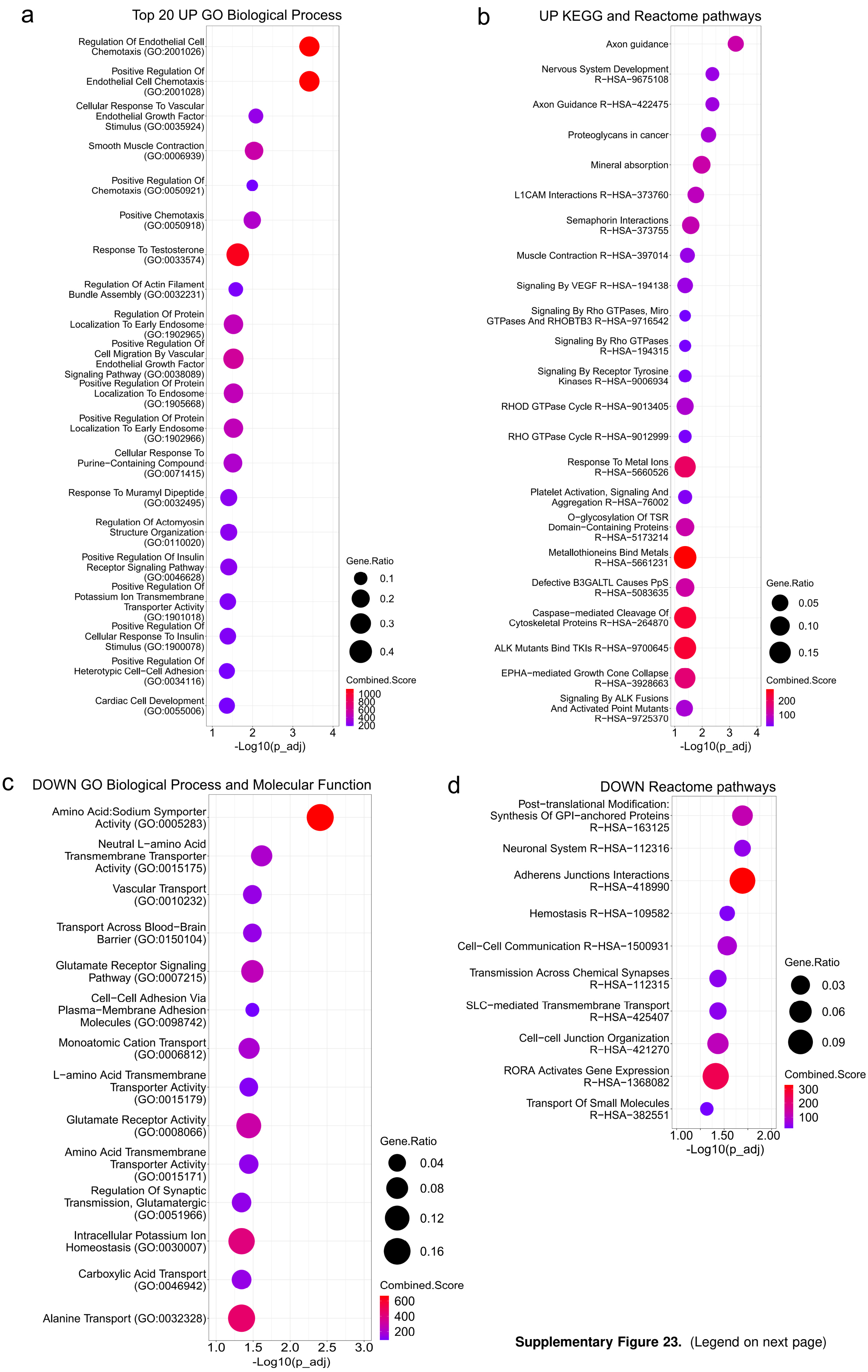

Supplementary Figure 23. (Legend on next page)

Supplementary Figure 23. Enrichment analysis of AC_4 marker genes. Results of functional enrichment analyses of DEGs (|log2fold change ≥ 0.6| and Bonferroni-adjusted p-value < 0.05) up- or downregulated in reactive astrocyte sub cluster AC_4 relative to homeostatic astrocytes (AC_1 and AC_2, pooled). Up to Top 20 enriched terms, subset by combined score and order by –log10 of Benjamini-Hochberg method adjusted p values are depicted as dotplots. **a** Enriched GO Biological Process terms associated to DEGs upregulated in AC_4. **b** Enriched KEGG and Reactome pathways associated to DEGs upregulated in AC_4. **c** Enriched GO Biological Process and GO Molecular Function terms associated to DEGs downregulated in AC_4. **d** Enriched Reactome pathways associated to DEGs downregulated in AC_4.

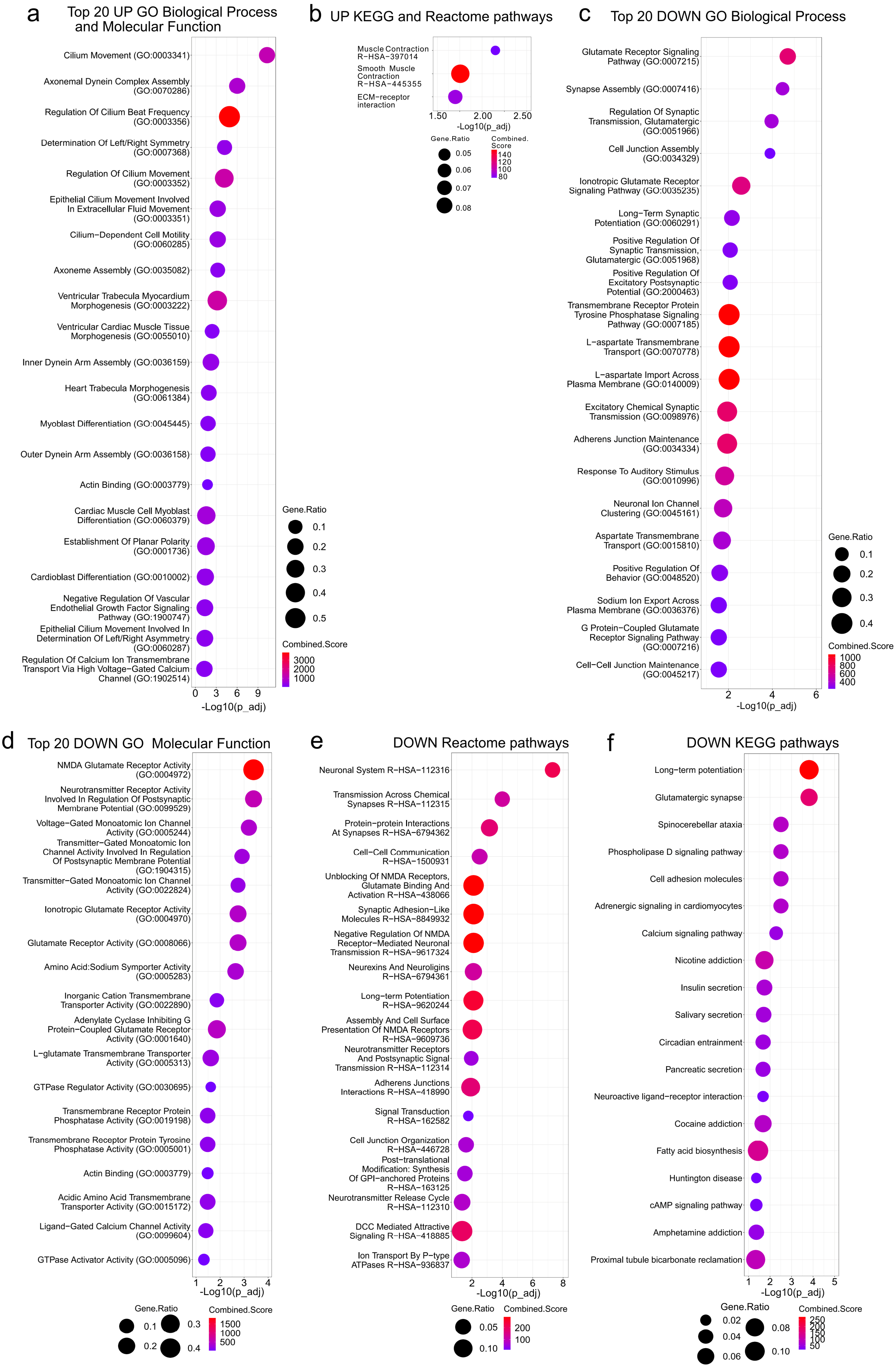

Supplementary Figure 24. (Legend on next page)

Supplementary Figure 24. Enrichment analysis of AC_5 marker genes. Results of functional enrichment analyses of DEGs (|log2fold change ≥ 0.6| and Bonferroni-adjusted p-value < 0.05) up- or downregulated in reactive astrocyte sub cluster AC_5 relative to homeostatic astrocytes (AC_1 and AC_2, pooled). Up to Top 20 enriched terms, subset by combined score and order by –log10 of Benjamini-Hochberg method adjusted p values are depicted as dotplots. **a** Enriched GO Biological Process and Molecular Function terms associated to DEGs upregulated in AC_5. **b** Enriched KEGG and Reactome pathways associated to DEGs upregulated in AC_5. **c,d** Enriched GO Biological Process (**c**) and GO Molecular Function (**d**) terms associated to DEGs downregulated in AC_5. **e,f** Enriched Reactome (**e**) and KEGG (**f**) pathways associated to DEGs downregulated in AC_5.

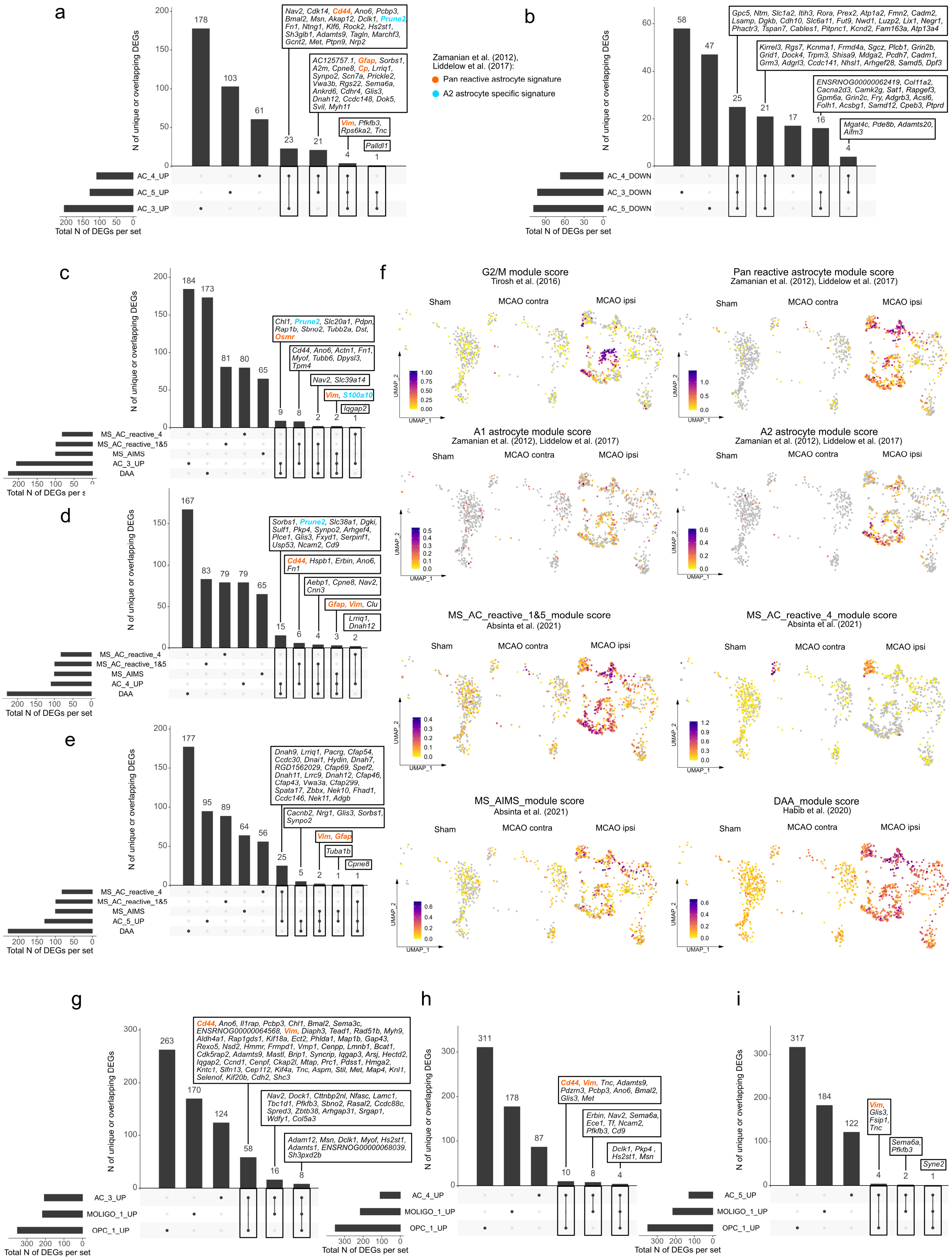

Supplementary Figure 25. (Legend on next page)

Supplementary Figure 25. Transcriptional signatures of reactive astrocytes in stroke compared to other neuropathologies. a,b UpSet plots depicting the numbers of total, unique and overlapping signature genes of the stroke associated reactive astrocyte (AC) subclusters (AC_3 to AC_5). All DEGs (Bonferroni-adjusted p-values < 0.05 and |log2fold change ≥ 0.6|) derived from the separate comparison of the reactive astrocyte subclusters AC_3, AC_4, and AC_5 to the homeostatic astrocyte subclusters (AC_1 and AC_2, pooled) were included. **a** upregulated, **b** downregulated DEGs of AC_3 to AC_5. Genes also included in the pan reactive astrocyte or A2 astrocyte gene sets, are highlighted in orange and cyan, respectively. **c-e** UpSet plots depicting the numbers of total, unique and overlapping signature genes of the stroke associated reactive astrocyte subclusters AC_3 (**c**), AC_4 (**d**), AC_5 (**e**), as well as reactive astrocyte signatures identified in multiple sclerosis (MS) and neurodegeneration (= disease associated astrocytes (DAA)). Gene sets used in this analysis are detailed in supplementary data file 1. **f** Module score feature plots projecting aggregate gene expressions of MS reactive astrocyte and DAA signatures onto astrocyte subclustering UMAP plots, split by treatment group. **g-i** UpSet plots depicting the numbers of total, unique and overlapping signature genes in reactive astrocytes and stroke associated oligodendrocyte lineage sub clusters. Up regulated DEGs in AC_3 (**g**), AC_4 (**h**) and AC_5 (**i**) are compared to upregulated DEGs in OPC_1 and MOLIGO_1.

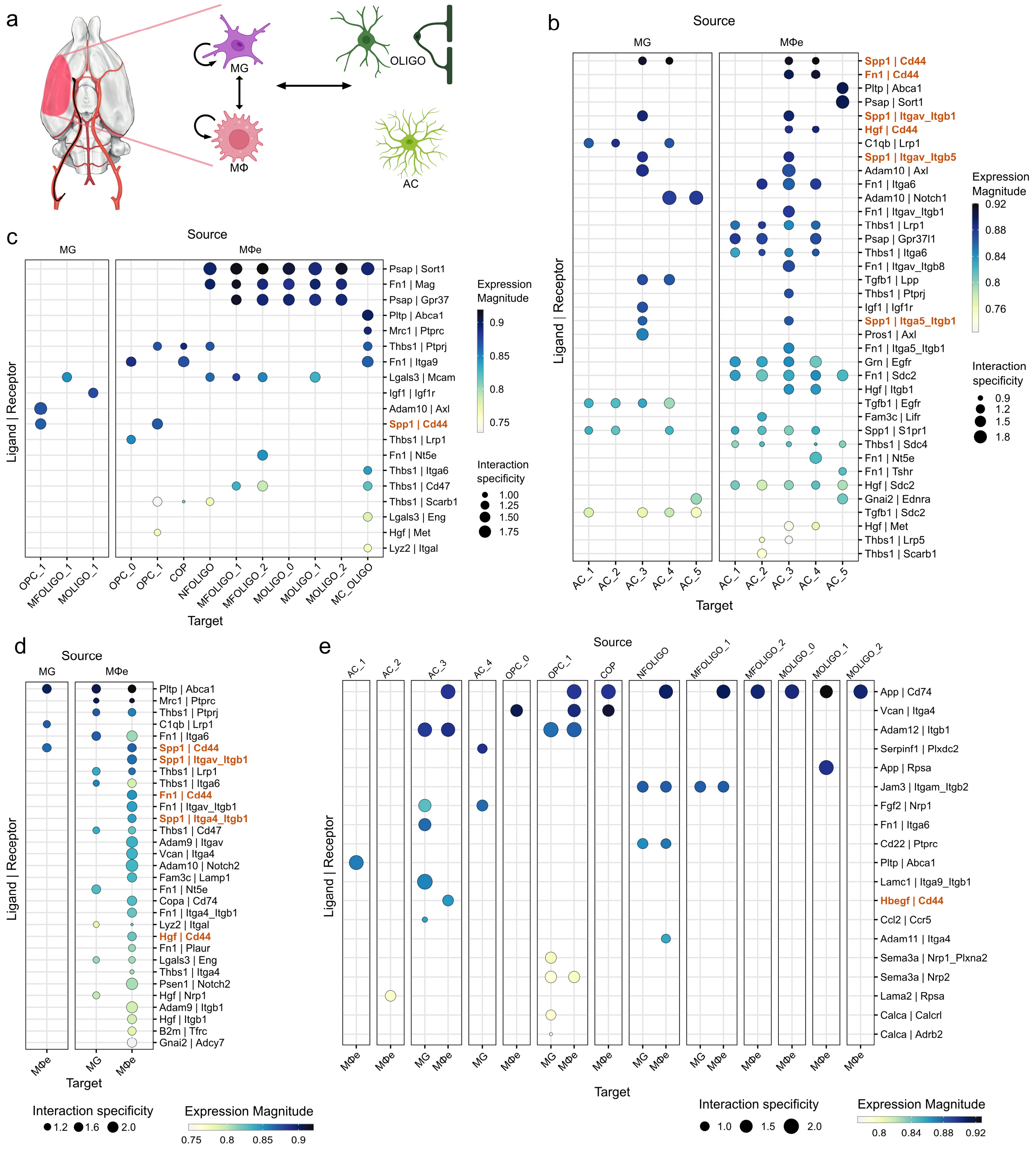

Supplementary Figure 26. Cell-cell communication analysis infers immuno-glial cross talk within infarcted brain tissue. a Illustration of cell-cell communication events included in this analysis. Illustrated using BioRender.com and SketchBook. **b-e** Dotplots depict all significant (aggregate rank scores < 0.05) ligand-receptor (LR) interactions, unique to datasets derived from infarcted tissue (MCAO ipsi). LR interactions between myeloid cell clusters as sources and astrocyte (**b**) and oligodendrocyte lineage cell clusters (**c**) as targets, between myeloid cell clusters (**d**) and neuroglia clusters as sources and myeloid cell clusters as targets (**e**) are shown. Interaction specificities (= NATMI edge specificity weights) are depicted as dot sizes and LR expression magnitudes (= SingleCellSignalR LR scores) are color coded. All interactions including Spp1 as ligand and/or Cd44 as receptor are highlighted in orange. Abbreviations: AC: Astrocyte, OLIGO: Oligodendrocyte lineage cells, MG: microglia, MΦ: macrophage, MΦe: macrophage enriched clusters, OPC: oligodendrocyte precursor cell, COP: committed oligodendrocyte precursor, NFOLIGO: newly formed oligodendrocyte, MFOLIGO: myelin forming oligodendrocyte, MOLIGO: mature oligodendrocyte, MC_OLIGO: myeloid cell oligodendrocyte mixed cluster.

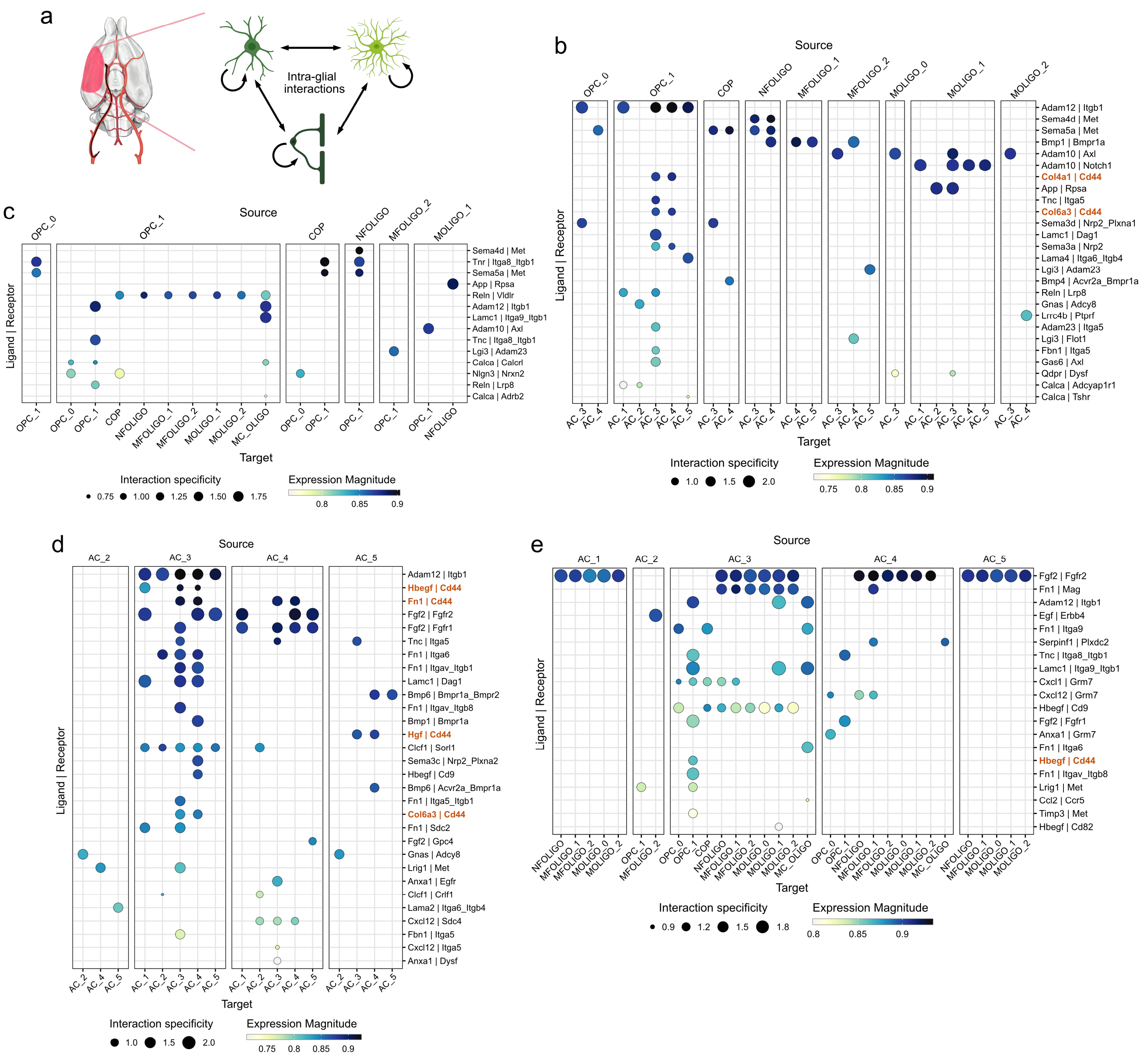

Supplementary Figure 27. Cell-cell communication analysis infers intra-glial cross talk within infarcted brain tissue. a Illustration of cell-cell communication events included in this analysis. Illustrated using BioRender.com and SketchBook. **b-e** Dotplots depict all significant (aggregate rank scores < 0.05) ligand-receptor (LR) interactions, unique to datasets derived from infarcted brain tissue (MCAO ipsi). LR interactions between oligodendrocytes as sources and astrocyte (**b**) and oligodendrocyte lineage cell clusters (**c**) as targets, between astrocyte subclusters (**d**) and between astrocyte subclusters as sources and oligodendrocyte subclusters as targets (**e**) are shown. Interaction specificities (= NATMI edge specificity weights) are depicted as dot sizes and LR expression magnitudes (= SingleCellSignalR LR scores) are color coded. All interactions including Spp1 as ligand and/or Cd44 as receptor are highlighted in orange. Abbreviations: AC: Astrocyte, OLIGO: Oligodendrocyte lineage cells, MG: microglia, MΦ: macrophage, MΦe: macrophage enriched clusters, OPC: oligodendrocyte precursor cell, COP: committed oligodendrocyte precursor, NFOLIGO: newly formed oligodendrocyte, MFOLIGO: myelin forming oligodendrocyte, MOLIGO: mature oligodendrocyte, MC_OLIGO: myeloid cell oligodendrocyte mixed.

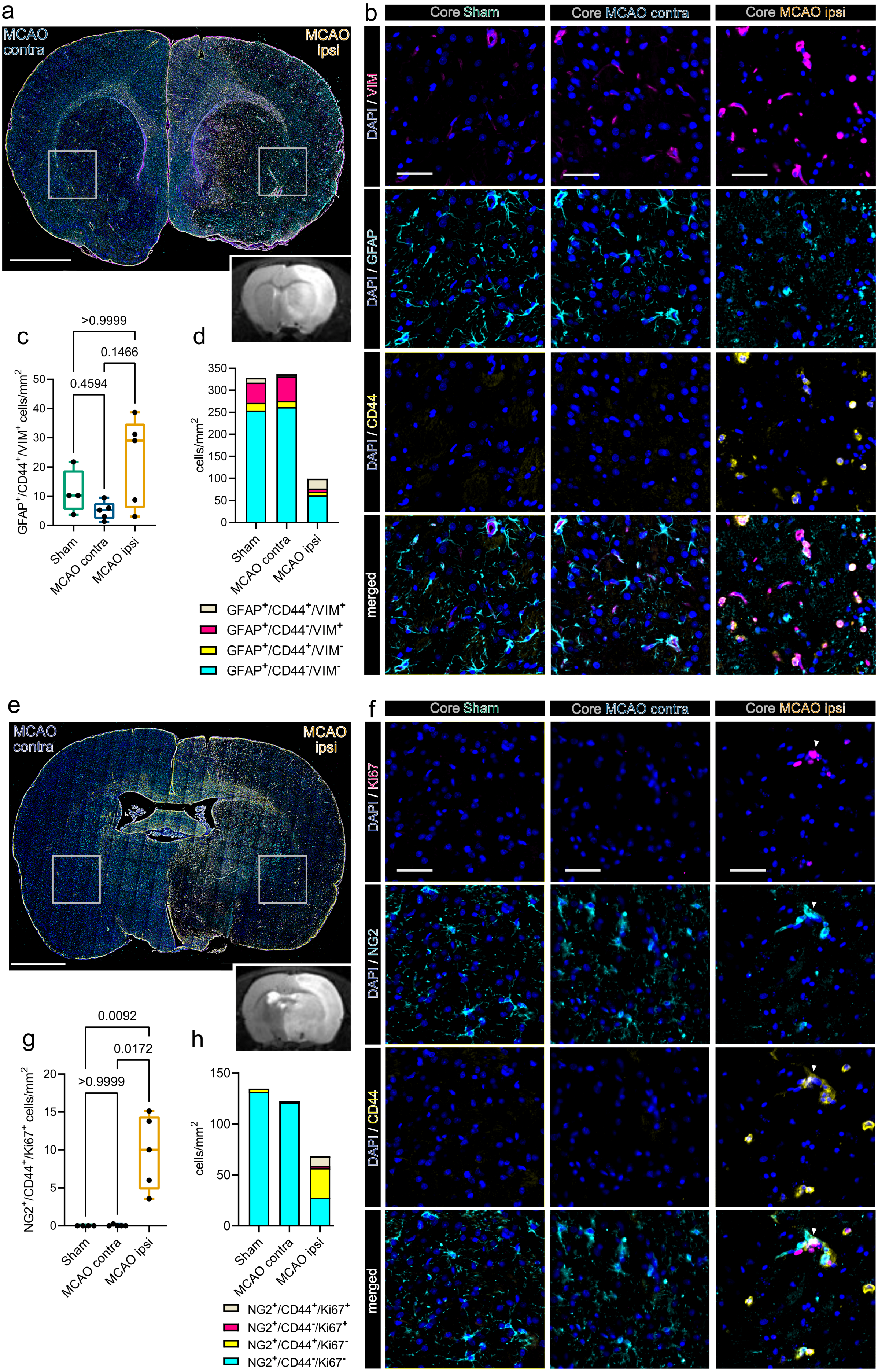

Supplementary Figure 28. (Legend on next page)

Supplementary Figure 28. Identification of CD44 positive reactive astrocytes and OPCs within the lesion core, 48h after filament based permanent MCAO in rats. a Overview of a representative coronal rat brain section 48 h post middle cerebral artery occlusion (MCAO), stained for GFAP, CD44 and VIM. Grey boxes highlight lesion core regions of interest (ROIs) within infarcted tissue (MCAO ipsi) and the anatomically corresponding ROI in the hemisphere contralateral to infarction (MCAO contra), lower right inset depicts a corresponding T2 weighted MRI image from the same animal. Bar = 2 mm. **b** Representative images from the lesion core ROI (MCAO ipsi), corresponding contralateral ROI (MCAO contra) and corresponding ROI in Sham operated rats (Sham), split by antigen. VIM = magenta, GFAP = Cyan, CD44 = yellow, DAPI (nuclei) = blue, bars = 50 µm. **c** Cell counts within lesion core and corresponding ROIs are presented as box plots for $GFAP^{+}/CD44^{+}/VIM^{+}$ triple positive cells. **d** Cell counts for $GFAP^{+}/CD44^{+}/VIM^{+}$, $GFAP^{+}/CD44^{-}/VIM^{+}$, $GFAP^{+}/CD44^{+}/VIM^{-}$, $GFAP^{+}/CD44^{-}/VIM^{-}$ positive cells are also jointly shown as colored stacked bar plots. **e** Overview of a representative coronal rat brain section 48 h post MCAO, stained for NG2, CD44 and Ki67. Grey boxes highlight lesion core regions of interest (ROIs) within MCAO ipsi and corresponding ROIs in MCAO contra, lower right inset depicts a corresponding T2 weighted MRI image from the same animal. Bar = 2 mm. **f** Representative images from the lesion core ROI (MCAO ipsi), corresponding contralateral ROI (MCAO contra) and corresponding ROI in Sham operated rats (Sham), split by antigen. Ki67 = magenta, NG2 = Cyan, CD44 = yellow, DAPI (nuclei) = blue, bars = 50 µm. **g** Cell counts within lesion core and corresponding ROIs are presented as box plots for $NG2^{+}/CD44^{+}/VIM^{+}$ triple positive cells. Cell counts for $NG2^{+}/CD44^{+}/VIM^{+}$, $NG2^{+}/CD44^{-}/VIM^{+}$, $NG2^{+}/CD44^{+}/VIM^{-}$, $NG2^{+}/CD44^{-}/VIM^{-}$ positive cells are also jointly shown as colored stacked bar plot. Data derived from n = 4 Sham control and n=5 MCAO group animals, p values derived from Kruskal-Wallis-H-Tests, followed by Dunn's post hoc comparisons. All Box plots depict medians, 25th to 75th percentiles as hinges, minimal and maximal values as whiskers, and individual counts, for each animal as dots. Statistical comparison of all subsets within all ROIs is reported in Supplementary Data 7. Source data are provided with this paper.

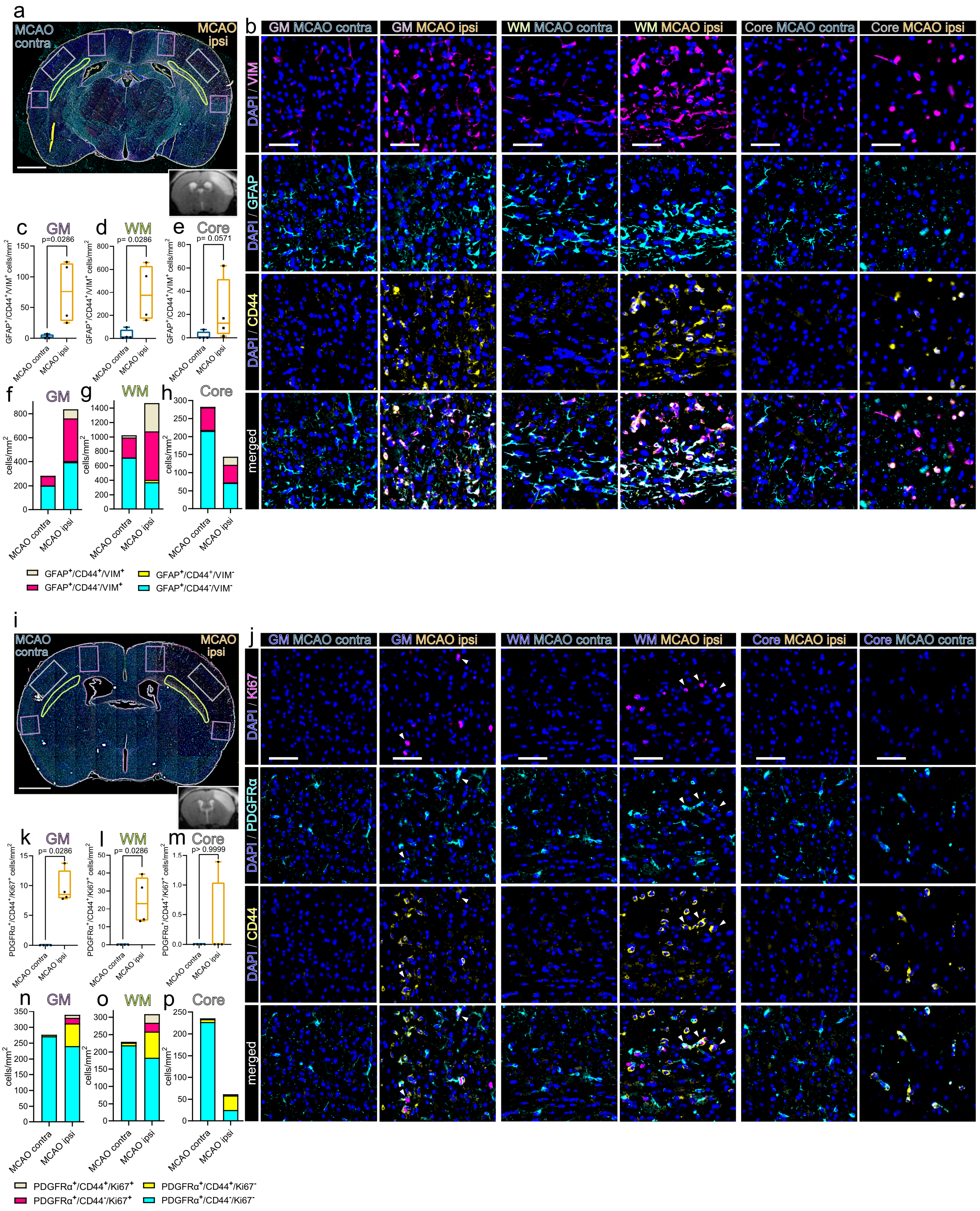

Supplementary Figure 29. (Legend on next page)

Supplementary Figure 29. Reactive astrocytes and proliferating OPCs are CD44 positive and abundant in the perilesional zone 48 h after thromboembolic MCAO in mice. a Overview of a representative coronal mouse brain section 48 h post MCAO, stained for GFAP, CD44 and VIM. Grey matter ROIs (GM) are highlighted in violet, white matter ROIs (WM) in lime green and the lesion core and its corresponding region in the contralateral hemisphere are highlighted in grey. Lower right inset depicts a corresponding T2 weighted MRI image from the same animal. Bar = 1mm. **b** Representative images from GM, WM and core ROIs within the infarcted (MCAO ipsi) and contralateral (MCAO contra) hemisphere, split by antigen. VIM = magenta, GFAP = Cyan, CD44 = yellow, all overlaid with DAPI (nuclei) = blue. Bars = 50 µm. **c-e** Cell counts within GM (**c**), WM (**d**) and core (**e**) ROIs are presented as box plots for $GFAP^{+}/CD44^{+}/VIM^{+}$ triple positive cells. **f-h** Cell counts for $GFAP^{+}/CD44^{+}/VIM^{+}$, $GFAP^{+}/CD44^{-}/VIM^{+}$, $GFAP^{+}/CD44^{+}/VIM^{-}$, $GFAP^{+}/CD44^{-}/VIM^{-}$ are jointly shown as colored stacked bar plots, for GM (**f**), WM (**g**) and core (**h**) ROIs. **i** Representative coronal overview, 48 h post MCAO, stained for PDGFRα, CD44, Ki67. GM ROIs in violet, WM ROIs in lime green, core ROIs in grey, lower right inset shows corresponding MRI image from the same animal. Bar = 1mm. **j** Representative images from GM, WM and core ROIs within the infarcted (MCAO ipsi) and contralateral (MCAO contra) hemisphere, split by antigen. Ki67 = magenta, PDGFRα = Cyan, CD44 = yellow. Bars = 50 µm. White arrowheads point to $PDGFR\alpha^{+}/CD44^{+}/Ki67^{+}$ triple positive cells. **k-m** Cell counts within GM (**k**) WM (**l**) and core (**m**) ROIs respectively are presented as box plots for $PDGFR\alpha^{+}/CD44^{+}/Ki67^{+}$. **n-p** Cell counts for $PDGFR\alpha^{+}/CD44^{+}/Ki67^{+}$, $PDGFR\alpha^{+}/CD44^{-}/Ki67^{+}$, $PDGFR\alpha^{+}/CD44^{+}/Ki67^{-}$, $PDGFR\alpha^{+}/CD44^{-}/Ki67^{-}$ are jointly shown as colored stacked bar plots, for GM (**n**), WM (**o**), Core (**p**). Data derived from n = 4 animals per group, p values derived from Mann-Whitney-U-tests. All Box plots depict medians, 25th to 75th percentiles as hinges, minimal and maximal values as whiskers, and individual counts, for each animal as dots. Statistical comparison of all subsets within all ROIs is reported in Supplementary Data 8. Source data are provided with this paper.

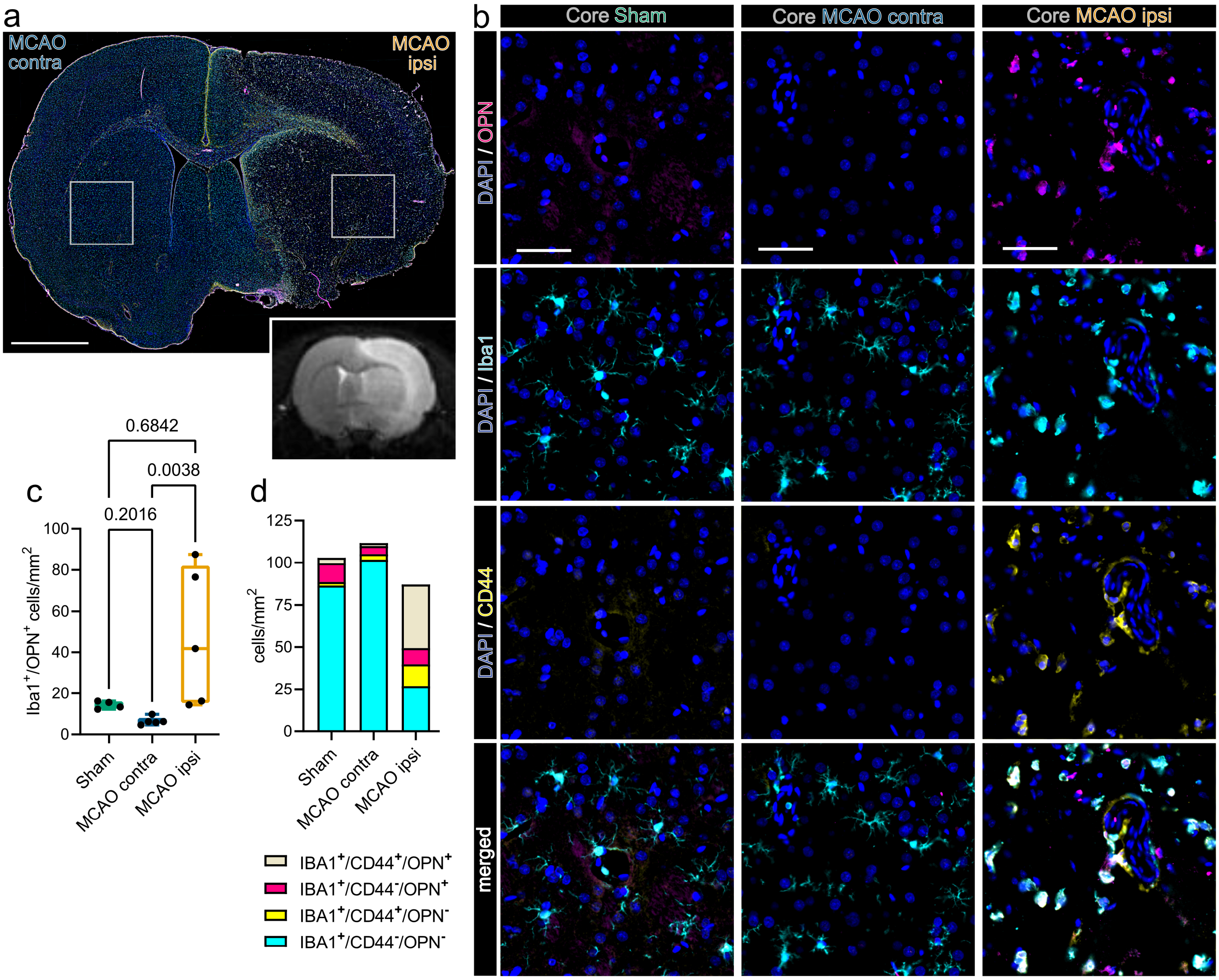

Supplementary Figure 30. Identification of osteopontin positive myeloid cells within the lesion core, 48h after filament based permanent MCAO in rats. a Overview of a representative coronal rat brain section 48 h post MCAO, stained for Iba1, CD44 and OPN. Grey boxes highlight lesion core regions of interest (ROIs) within infarcted tissue (MCAO ipsi) and the anatomically corresponding ROI in the hemisphere contralateral to infarction (MCAO contra), lower right inset depicts a corresponding T2 weighted MRI image from the same animal. Bar = 2 mm. **b** Representative images from the lesion core ROI (MCAO ipsi), corresponding contralateral ROI (MCAO contra) and corresponding ROI in Sham operated rats (Sham), split by antigen. OPN = magenta, Iba1 = cyan, CD44 = yellow. Bars = 50 µm. **c** Cell counts within lesion core and anatomically corresponding ROIs derived from Sham and MCAO contra are presented as box plots for Iba1+/OPN+ double positive cells. Box plots depict medians, 25th to 75th percentiles as hinges, minimal and maximal values as whiskers, and individual counts, for each animal as dots. **d** Cell counts for Iba1+/CD44+/OPN+, Iba1+/CD44-/OPN+, Iba1+/CD44+/OPN-, Iba1+/CD44-/OPN- are jointly shown as colored stacked bar plots. Data derived from from n = 4 Sham control and n=5 MCAO group animals, p values derived from Kruskal-Wallis-H-Tests, followed by Dunn's post hoc comparisons. Statistical comparison of all subsets within all ROIs is reported in Supplementary Data 7. Source data are provided with this paper.

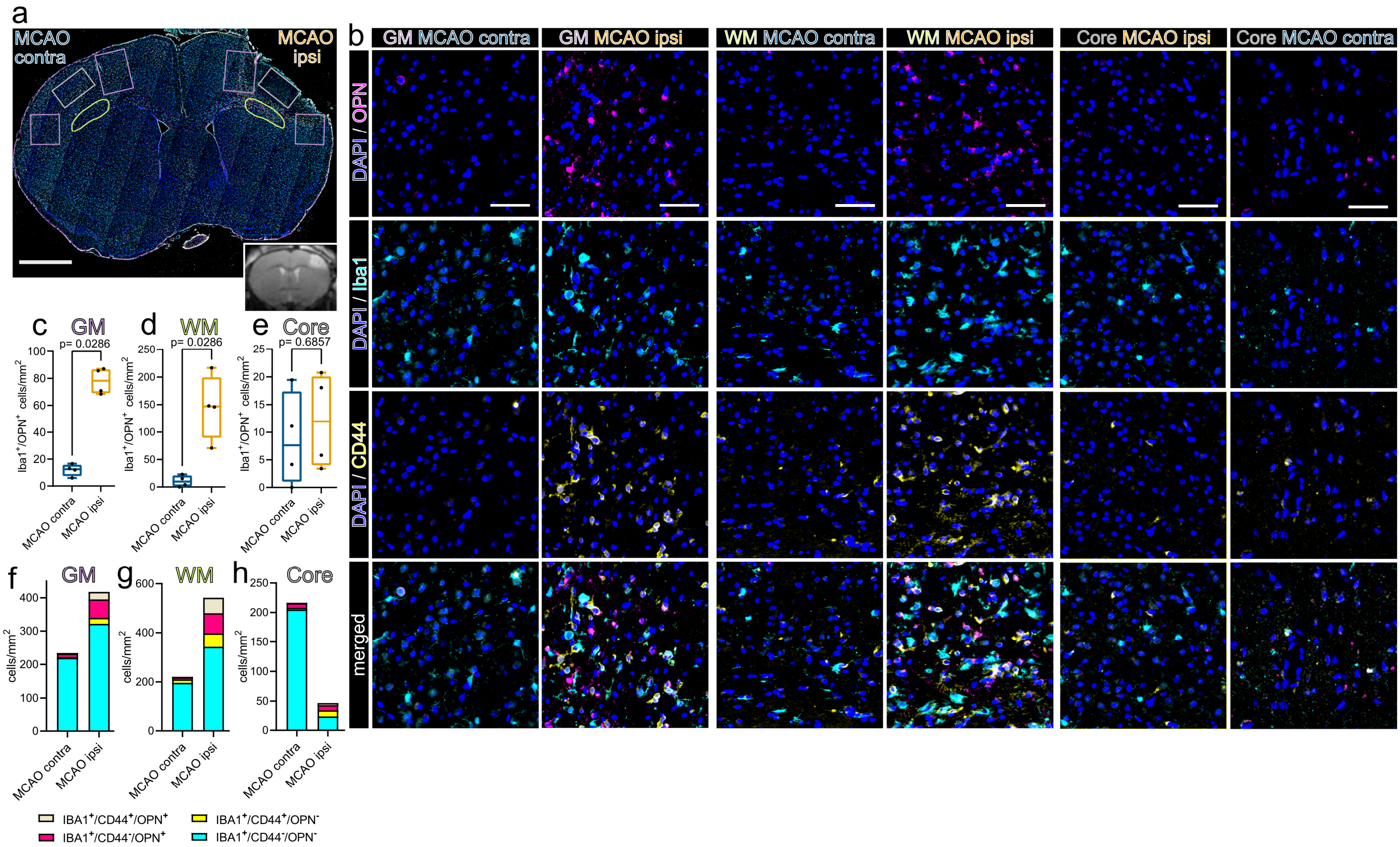

Supplementary Figure 31. Osteopontin positive myeloid cells accumulate in the perilesional zone in close proximity to CD44 positive cells 48 h after thromboembolic MCAO in mice. a Overview of a representative coronal mouse brain section 48 h post MCAO, stained for Iba1, CD44 and OPN. Grey matter ROIs (GM) are highlighted in violet, white matter ROIs (WM) in lime green and the lesion core and its corresponding region in the contralateral hemisphere are highlighted in grey. Lower right inset depicts a corresponding T2 weighted MRI image from the same animal. Bar = 1mm. **b** Representative images from GM, WM and core ROIs within the infarcted (MCAO ipsi) and contralateral (MCAO contra) hemisphere, split by antigen. OPN = magenta, Iba1 = cyan, CD44 = yellow. Bars = 50 µm. **c-d** Cell counts within GM (**c**), WM (**d**) and core (**e**) ROIs are presented as box plots for $Iba1^+/OPN^+$ double positive cells, cell counts for $Iba1^+/CD44^+/OPN^+$, $Iba1^+/CD44^-/OPN^+$, $Iba1^+/CD44^+/OPN^-$, $Iba1^+/CD44^-/OPN^-$ are jointly shown as colored stacked bar plots, for GM (**f**), WM (**g**) and core (**h**) ROIs. Data derived from n = 4 animals per group, p values derived from Mann-Whitney-U-tests. All Box plots depict medians, 25th to 75th percentiles as hinges, minimal and maximal values as whiskers, and individual counts, for each animal as dots. Statistical comparison of all subsets within all ROIs is reported in Supplementary Data 8. Source data are provided with this paper.

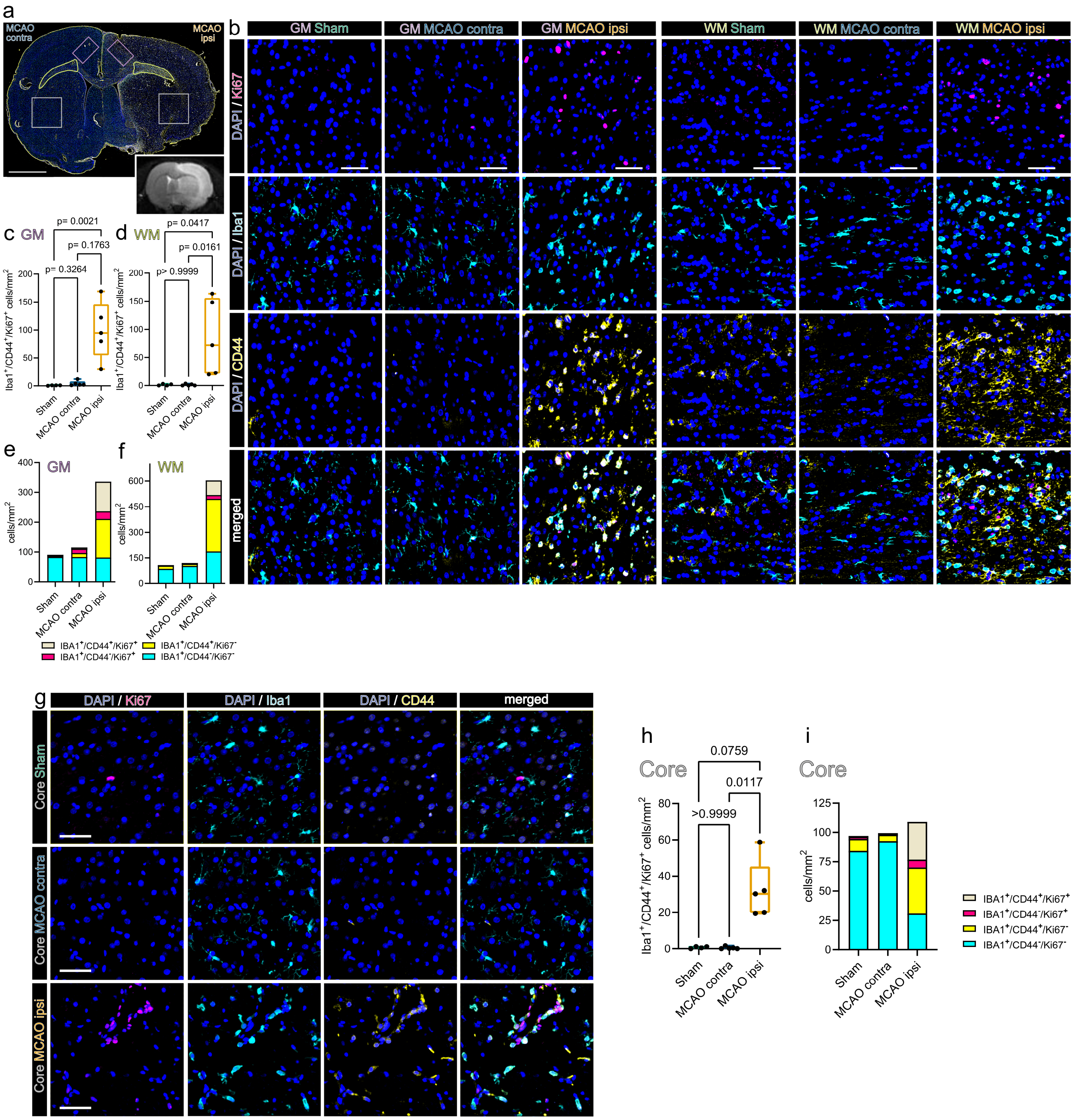

Supplementary figure 32. Abundant proliferating, CD44 positive myeloid cells are identified in the perilesional zone and lesion core. a Overview of a representative coronal rat brain section 48h post MCAO, stained for Iba1, CD44 and Ki67. Grey matter ROIs (GM) are highlighted in violet, white matter ROIs (WM) in lime green and the lesion core ROI and its corresponding ROI in the contralateral hemisphere are highlighted in grey, lower right inset depicts a corresponding T2 weighted MRI image from the same animal. Bar = 2 mm. **b** Representative images taken from and GM, WM ROIs of infarcted tissue (MCAO ipsi) the hemisphere contralateral to infarction (MCAO contra) and Sham operated animals (Sham), split by antigen. Ki67 = magenta, Iba1 = cyan, CD44 = yellow, all overlaid with DAPI (nuclei) = blue. Bars = 50 µm. **c,d** Cell counts within GM (**c**) and WM (**d**) are presented as box plots for Iba1$^+$/CD44$^+$/Ki67$^+$ triple positive cells. **e,f** Cell counts for Iba1$^+$/CD44$^+$/Ki67$^+$, Iba1$^+$/CD44$^-$/Ki67$^+$, Iba1$^+$/CD44$^+$/Ki67$^-$, Iba1$^+$/CD44$^-$/Ki67$^-$ are jointly shown as colored stacked bar plots, for GM (**e**) and WM (**f**) ROIs. **g** Representative images from lesion core and anatomically corresponding ROIs derived from Sham, MCAO contra and MCAO ipsi groups, split by antigen. Ki67 = magenta, Iba1 = Cyan, CD44 = yellow, DAPI (nuclei) = blue, bars = 50 µm. **h** Cell counts within lesion core and anatomically corresponding ROIs derived from Sham, MCAO contra and MCAO ipsi groups, are presented as box plots for Iba1$^+$/CD44$^+$/Ki67$^+$ triple positive cells. **i** Cell counts for Iba1$^+$/CD44$^+$/Ki67$^+$, Iba1$^+$/CD44$^-$/Ki67$^+$, Iba1$^+$/CD44$^+$/Ki67$^-$, Iba1$^+$/CD44$^-$/Ki67$^-$ are jointly shown as colored stacked bar plots. Data derived from n = 4 Sham control and n=5 MCAO group animals, p values derived from Kruskal-Wallis-H-Tests, followed by Dunn's post hoc comparisons. All Box plots depict medians, 25th to 75th percentiles as hinges, minimal and maximal values as whiskers, and individual counts, for each animal as dots. Statistical comparison of all subsets within all ROIs is reported in Supplementary Data 8. Source data are provided with this paper.

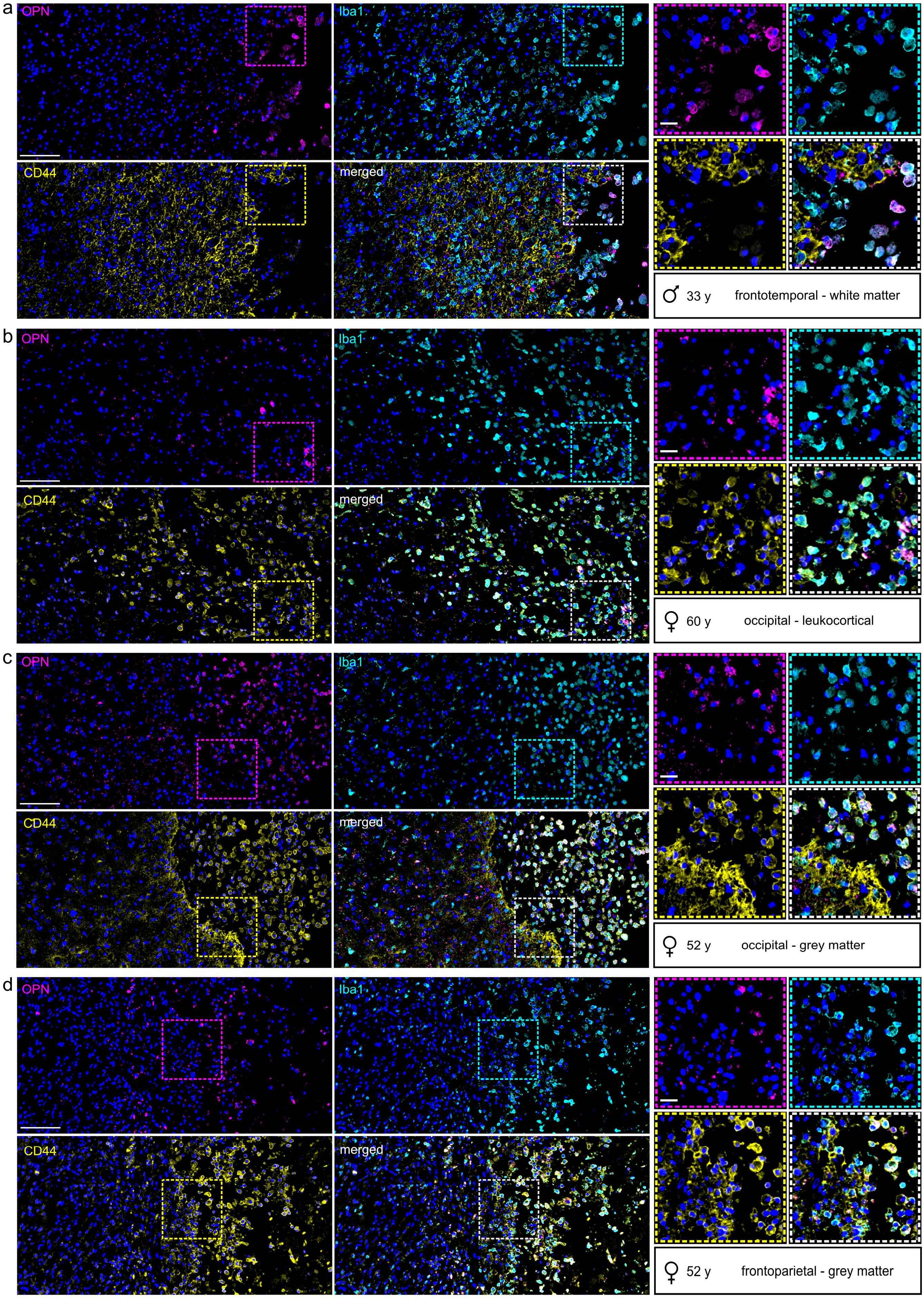

Supplementary Figure 33. (Legend on next page)

Supplementary figure 33. Spatial association of osteopontin positive myeloid cells to CD44 positive cells in human infarcted cerebral tissue. Human cerebral, biopsy derived tissue from n = 4 patients, is presented. Iba1, Ki67, OPN and nuclei were visualized using IF staining. **a - d** represent individual cases. Sex assigned at birth, age and sampled brain regions are given for each patient. All cases were staged as infarcted tissue in the stage of advanced macrophage resorption and beginning of pseudo cystic cavity formation (Stage II - III). For each case a 400 x 800 µm overview area at the border of macrophage resorption is presented, as well as 150 x 150 µm close up images, split by antigen, clockwise: OPN = magenta, Iba1 = cyan, CD44 = yellow, all channels merged = white, all channels were overlaid with DAPI (nuclei) = blue. Scale bars: 100 µm in overview, 20 µm in close ups.

Marker Gene	Main associated cell populations	Allen Atlas ISH Study	Hyperlink
Rasgrf2	L2/L3	Rasgrf2 - RP_051214_01_F08 - coronal	https://mouse.brain-map.org/gene/show/19181
Rorb	L4/L5	Rorb - RP_071018_01_H03 - coronal	https://mouse.brain-map.org/experiment/show/79556597
Serpine2	L5	Serpine2 - RP_051214_01_H06 - coronal	https://mouse.brain-map.org/gene/show/20482
Grik3	L5/6	Grik3 - RP_060606_03_A02 - coronal	https://mouse.brain-map.org/experiment/show/75749418
Tle4	L5/6	Tle4 - RP_051101_03_A08 - coronal	https://mouse.brain-map.org/experiment/show/73521809
Syt6	L6	Syt6 - RP_Baylor_103197 – coronal	https://mouse.brain-map.org/gene/show/33815
Synpr	L6/CLA	Synpr - RP_040507_02_G03 - coronal	https://mouse.brain-map.org/gene/show/47844
Gng2	L6/CLA	Gng2 - RP_040604_01_F06 - coronal	https://mouse.brain-map.org/experiment/show/67936006
Ndst4	LSr, PIR	Ndst4 - RP_060220_03_B07 - coronal	https://mouse.brain-map.org/gene/show/41177

Supplementary Table 1. Allen Brain Atlas database *in situ* hybridization (ISH) studies[1] referenced within this study. Abbreviations: L = layer (cortical layers L2 –L6), CLA = claustrum, LSr = lateral septal nucleus, PIR = piriform cortex.

Antibody	Source	Identifier
**Primary Antibodies**		
Rabbit polyclonal anti-NG2	Merck Millipore (Burlington, MA, USA)	Cat# AB5320, RRID:AB_91789
Rabbit anti-NG2 - Cy3® conjugated	Merck Millipore (Burlington, MA, USA)	Cat# AB5320C3, RRID:AB_11203295
Rabbit anti-BrdU - FITC conjugated	BD Bioscience (Franklin Lakes, NJ, USA)	From kit: Cat# 558662
Rabbit polyclonal anti-Ki67	Abcam (Cambridge, UK)	Cat# ab15580, RRID:AB_443209
Rabbit polyclonal anti-CD44	Abcam (Cambridge, UK)	Cat# ab157107, RRID:AB_2847859
Recombinant rabbit monoclonal anti-Iba1	Abcam (Cambridge, UK)	Cat# ab178846, RRID:AB_2636859
Chicken polyclonal anti-GFAP	Abcam (Cambridge, UK)	Cat# ab4674, RRID:AB_304558
Rabbit polyclonal anti-Osteopontin	Abcam (Cambridge, UK)	Cat# ab63856, RRID:AB_1524127
Mouse monoclonal IgG1 anti-Vimentin, Clone V9	DAKO - Agilent Technologies (Santa Clara, CA, USA)	Cat# M0725, RRID:AB_10013485
Rabbit monoclonal anti-Vimentin antibody	Abcam (Cambridge, UK)	Cat# ab92547, RRID:AB_10562134
Rabbit recombinant monoclonal anti-IL33	Abcam (Cambridge, UK)	Cat# ab187060, RRID:AB_2894704
Rabbit recombinant monoclonal anti-IL33	Abcam (Cambridge, UK)	Cat# ab207737, RRID:AB_2827630
Rabbit monoclonal anti-PDGF Receptor α	Cell Signaling Technology (Danvers, MA, USA)	Cat# D1E1E XP® #3174, RRID:AB_2162345
Mouse monoclonal anti-MAP2	Merck Millipore (Burlington, MA, USA)	Cat#: MAB3418, RRID:AB_94856
**Secondary Antibodies**		
Goat Anti-Rabbit IgG H&L (Alexa Fluor® 488)	Abcam (Cambridge, UK)	Cat# ab150077, RRID:AB_2630356
Goat anti-Mouse IgG1 Cross-Adsorbed Secondary Antibody (Alexa Fluor® 546)	Thermo Fisher Scientific (Waltham, MA, USA)	Cat# A-21123, RRID:AB_2535765
Goat anti-Chicken IgY (H+L) Secondary Antibody (Alexa Fluor® 647)	Thermo Fisher Scientific (Waltham, MA, USA)	Cat# A-21449, RRID:AB_2535866

Antibody labelling kits		
FlexAble CoraLite® Plus 488 Antibody Labeling Kit for Rabbit IgG	Proteintech (Rosemont, IL, USA)	Cat#: KFA001
FlexAble CoraLite® Plus 555 Antibody Labeling Kit for Rabbit IgG	Proteintech (Rosemont, IL, USA)	Cat# KFA002
FlexAble CoraLite® Plus 647 Antibody Labeling Kit for Rabbit IgG	Proteintech (Rosemont, IL, USA)	Cat# KFA003

Supplementary Table 2. Antibodies and antibody labelling kits used in this study

Colocalized targets	HIER	Non conjugated primary antibodies		Corresponding secondary antibodies		Fluorophore conjugated antibodies		
		Antibody	Dilution	Antibody	Dilution	Antibody	Labelling kit used	Final working con-centration
MAP2+Iba1 (in all species)	pH6	MAP2 (MAB3418)	1:1000	Goat anti-Mouse (Alexa Fluor® 546)	1:500			
		Iba1 (ab178846)	1:400	Goat Anti-Rabbit (Alexa Fluor® 488)	1:500			
MAP2+GFAP+ PDGFRα (in all species)	pH6	MAP2 (MAB3418)	1:1000	Goat anti-Mouse (Alexa Fluor® 546)	1:500			
		PDGFRα (D1E1E)	1:300	Goat Anti-Rabbit (Alexa Fluor® 488)	1:500			
		GFAP (ab4674)	1:800	Goat anti-Chicken (Alexa Fluor® 647)	1:500			
NG2+VIM+Ki67 (in rat tissue)	pH9	NG2 (AB5320)	1:100	Goat Anti-Rabbit (Alexa Fluor® 488)	1:500	Ki67 (ab15580)	FlexAble CoraLite® Plus 647	2 µg/ml
		VIM (M0725)	1:200	Goat anti-Mouse (Alexa Fluor® 546)	1:500			
PDGFRα+VIM+Ki67 (in mouse tissue)	pH6	PDGFRα (D1E1E)	1:300	Goat Anti-Rabbit (Alexa Fluor® 488)	1:500	VIM (ab92547)	FlexAble CoraLite® Plus 555	2,3 µg/ml
						Ki67 (ab15580)	FlexAble CoraLite® Plus 555	2 µg/ml
NG2+IL33+Ki67 (in rat tissue)	pH9	NG2 (AB5320)	1:100	Goat Anti-Rabbit (Alexa Fluor® 488)	1:500	IL33 (ab207737)	FlexAble CoraLite® Plus 555	1.7 µg/ml
						Ki67 (ab15580)	FlexAble CoraLite® Plus 555	2 µg/ml
PDGFRα+IL33+Ki67 (in mouse tissue)	pH6	PDGFRα (D1E1E)	1:300	Goat Anti-Rabbit (Alexa Fluor® 488)	1:500	IL33 (ab187060)	FlexAble CoraLite® Plus 555	1.7 µg/ml
						Ki67 (ab15580)	FlexAble CoraLite® Plus 555	2 µg/ml
GFAP+CD44+VIM (in rat tissue)	pH6	GFAP (ab4674)	1:800	Goat anti-Chicken (Alexa Fluor® 647)	1:400			
		VIM (M0725)	1:200	Goat anti-Mouse (Alexa Fluor® 546)	1:500			
		CD44 (ab157107)	1:500	Goat Anti-Rabbit (Alexa Fluor® 488)	1:500			
GFAP+CD44+VIM (in murine tissue)	pH6	GFAP (ab4674)	1:800	Goat anti-Chicken (Alexa Fluor® 647)	1:400	VIM (ab92547)	FlexAble CoraLite® Plus 488	2,3 µg/ml
						CD44 (ab157107)	FlexAble CoraLite® Plus 555	1 µg/ml
NG2+CD44+Ki67 (in rat tissue)	pH9	NG2 (AB5320)	1:100	Goat Anti-Rabbit (Alexa Fluor® 488)	1:500	CD44 (ab157107)	FlexAble CoraLite® Plus 555	1 µg/ml
						Ki67 (ab15580)	FlexAble CoraLite® Plus 647	2 µg/ml
PDGFRα+CD44+ Ki67 (in mouse tissue)	pH6	PDGFRα (D1E1E)	1:300	Goat Anti-Rabbit (Alexa Fluor® 488)	1:500	CD44 (ab157107)	FlexAble CoraLite® Plus 555	1 µg/ml
						Ki67 (ab15580)	FlexAble CoraLite® Plus 647	2 µg/ml
Iba1+OPN+CD44 (in all species)	pH6	Iba1 (ab178846)	1:400	Goat Anti-Rabbit (Alexa Fluor® 488)	1:500	OPN (ab63856)	FlexAble CoraLite® Plus 555	3.3 µg/ml
						CD44 (ab157107)	FlexAble CoraLite® Plus 647	1 µg/ml
Iba1+CD44+Ki67 (in all species)	pH6	Iba1 (ab178846)	1:400	Goat Anti-Rabbit (Alexa Fluor® 488)	1:500	CD44 (ab157107)	FlexAble CoraLite® Plus 555	1 µg/ml
						Ki67 (ab15580)	FlexAble CoraLite® Plus 647	2 µg/ml

Supplementary Table 3 Antibody combinations, Heat induced epitope retrieval (HIER) pH and antibody dilutions are summarized for each performed Immunofluorescence staining.

Supplementary notes – Detailed description of cluster annotation

The overall interpretation of our results regarding cell type specific transcriptional perturbations in response to ischemic stroke is given in the main text. In this supplementary note we describe the performed clustering analyses and the curation of marker genes for cell cluster annotation in detail.

Main cell cluster annotation

Unsupervised clustering of the integrated dataset, using the first 23 principal components (PC) and a resolution of 0.4 revealed 32 clusters, which were then annotated to 29 major cell clusters, according to established marker genes (Fig.1b-e). Clusters were first grouped in neuronal and non-neuronal populations using the pan-neuronal markers *Rbfox3*, *Snap25* and *Syt1* [2, 3, 4, 5]. Among the non-neuronal populations, we identified two communities expressing overarching oligodendrocyte lineage markers, such as *Plp1*, canonical markers of oligodendrocyte precursor cells (OPC) and immature oligodendrocytes, such as *Vcan*, *Pdgfra* and *Bcas1* (cluster OLIGO_1) and markers of myelinating and mature oligodendrocytes, such as *Mbp*, *Mog* and *Apod* (cluster OLIGO_2) [6, 7]. We identified one cluster enriched in several canonical pan-astrocyte markers, such as *Slc1a2* and *Slc1a3* [8] and astrocyte associated markers such as *Aqp4* and *Gfap* [6, 9], which we annotated astrocyte cluster (AC). We observed the concomitant enrichment for several ependymal cell marker genes, such as *Tmem212, Ccdc162* [6], *Cfap299* [10] and *Dnah11* [9] in one cluster. Notably, the same cluster also faintly co-expressed *Pdgfra*, and several other genes concomitantly expressed in vascular leptomeningeal cell types such as *Dcn*, *Ptgds* and *Aldh1a2* [6]. Thus this cluster was annotated ependymal and mural cell cluster (EP_M_C), due to the possible contribution of both ependymal and mural cell derived transcripts. The expression of canonical endothelial cell markers such as *Pecam1*, *Vwf* and *Flt1* [9, 11] was restricted to one small cluster, which also expressed pericyte associated markers such as *Rgs5* and *Vtn* [11, 12]. Due to the small cluster size this cluster could not be resolved into endothelial, pericyte and mural cell subclusters and was annotated vascular cell cluster (VASC). Lastly, we identified one immune cell cluster, which was close to exclusively derived from infarcted brain hemispheres. This cluster selectively expressed the broad myeloid cell marker *Ptprc*, the immune cell enriched marker *Arhgap15*, microglia associated transcripts, such as *Hexb*, *Fcrl2* and *Cx3cr1*, shared markers of microglia and macrophages, such as *Aif1* and to a lesser degree macrophage and granulocyte associated marker genes such as *Apoe*, *Lyz2* and *Cd14* [13, 14, 15]. We identified a substantial overlap between the core stroke-associated myeloid cell (SAMC) signature, consisting of the TOP 10 SAMC marker genes: *Spp1, Fabp5, Gpnmb, Ctsb, Ctsl, Lgals3, Lpl, Fth1, Cd63*, and *Ctsd* [14] and the transcriptional signature of the immune cells in our dataset. Notably, lymphoid lineage cluster markers such as the T-cell markers *Cd3e*, *Cd3g*, B-cell markers *Cd19*, *Cd79a*, innate lymphoid cell type marker *Gata3* and NK cell markers *Nkg7* and *Gzma* [14, 15] were virtually absent in our dataset. Details on the subclustering analysis of infarction enriched myeloid cells are given below.

Neuronal clusters were grouped first into excitatory and inhibitory populations, using the established markers *Slc17a7*, *Slc17a6* and *Sv2b* for excitatory, glutamatergic neurons and *Gad1* and *Gad2* for inhibitory, GABAergic neurons [16, 17, 18].

Glutamatergic neurons were first segregated into 2 broad communities, defined by the absence or expression of *Satb2*. Glutamatergic *Satb2* positive (GLU_Satb2+) and negative (GLU_Satb2-) populations were then further characterized by the expression of known markers of topographical identity. In agreement with the observation that *Satb2* is highly enriched in isocortical excitatory neurons [9, 19], the marker genes of the *Satb2* positive populations GLU_Satb2+_1 to 9, largely overlapped with established transcriptional signatures associated to specific cortical layers (Fig. 1c).

GLU_Satb2+_1 exhibited concomitant enrichment for the L2 associated marker *Otof* [20], the recently identified L2/3 specific marker *Ccbe1* [21], as well as the L2/3 associated markers *Rasgrf2*, *Glis3*, *Nectin3*, *Cux1* and *Cux2* [4, 17, 20, 22, 23]. *Cux1* and *Cux2* were also enriched in GLU_Satb2+_2 and GLU_Satb2+_8. Notably, *Cux1* and *2* are also expressed in deeper cortical layers, for example adjacent to the Claustrum, or layer 4 cortical neurons [20, 21]. The established cortical layer 4-5 marker *Rorb* [9, 17], as well as the *Rorb* target *Thsd7a* [24] were concomitantly enriched in GLU_Satb2+_2 and GLU_Satb2+_3. GLU_Satb2+_3 also expressed the cortical layer 4 to 5 associated marker *Il1rapl2* [17], while the layer 5 and 6 associated markers *Tox* and *Grik3*, [4, 9, 17], where co-expressed to varying degrees from GLU_Satb2+_3 to GLU_Satb2+_7. GLU_Satb2+_4 to 7 expressed *Bcl11b*, which was shown to be highly expressed in layer 5 cortical neurons [25]. Correspondingly, GLU_Satb2+_4 concomitantly expressed the layer 5 associated marker *Parm1* [11] and *Serpine2*, which was previously used as markers of cortical Layer 5b identity in the adult rodent motor cortex [26]. GLU_Satb2+_5 to 7 prominently expressed *Tle4*, which recent evidence suggested as a specific marker of layer 6 corticothalamic projection neurons [27]. GLU_Satb2+_5 and 6 also expressed the corticothalamic projection neuron associated marker *Thsd7b* [20]. GLU_Satb2+_6 was strongly enriched for *Foxp2*, which was associated to cortical layers 5 and 6 [17, 28], as well as the layer 6 associated marker *Syt 6* [9, 22]. Of note, the *Tle4*, *Foxp2* double positive cluster GLU_Satb2+_7, strongly expressed *Vwc2l*, a marker of L5/6 near-projecting neurons [20]. This cluster however also expressed the lateral cortex layer 6 marker *Col24a1* [6] and the cortical layer 5 and 6 associated marker *Tshz2* [28], whereas GLU_Satb2+_8 most prominently expressed the L6 and claustrum associated markers *Synpr* [22, 29] and *Nr4a2* [9, 22] , corroborated by the prominent and selective expression of the claustrum enriched gene *Gng2* (Fig. 1c). GLU_Satb2+_7 and 8 further shared the concomitant expression of Grin3a and the layer 5 and 6 associated marker *Htr2c* [4].

GLU_Satb2+_9 shared several L5 and L6 associated features with GLU_Satb2+_3, but distinctly expressed several allocortex associated marker genes, such as *Rspo2* and *Rxfp1*, which are known to be enriched in subcortical structures such as the basolateral amygdala [30], the hippocampal formation, or various diencephalic structures [31]. GLU_Satb2+_9 further exhibited enrichments for *Sema3e* and *Adamts3*. GLU_Satb2-_1 and 2 both coexpressed *Abi3bp* and *Ndst4*, which have been associated to glutamatergic neurons in anterior olfactory areas [6]. In contrast to its neighbouring cluster, GLU_Satb2-_1 strongly and specifically expressed *Ankfn1*, which was shown to be enriched in the superchiasmatic nucleus [32]. This cluster also prominently expressed *Ntng1* which was shown to be particularly enriched in hypothalamic and thalamic areas [33]. GLU_Satb2-_3 showed a strong enrichment for the prosubiculum associated marker *Arhgap6* and expressed Rerg and Pkp2, which are enriched in the hippocampal formation [20]. Congruently, this cluster was also enriched for *Rasgrf2* and *Rfx3*, which are particularly densely expressed in upper layer cortical and hippocampal pyramidal cells (Fig. 1c). We identified one cluster (CHOL_IN) co-expressing *Chat*, *Lhx8*, *Slc17a8* and *Slc5a7*, matching the transcriptional signature of cholinergic interneurons [6].

GABAergic populations were segregated into clusters matching known interneuron (GABA_IN) and GABAergic medium spiny neuron identities (GABA_MSN). In accordance with previously established annotation strategies [16, 20, 28] we first broadly separated the GABAergic neuronal clusters into *Adarb2* positive (GABA_IN_Adarb2+), thus likely caudal ganglionic eminence (CGE) derived and Adarb2 negative (GABA_IN_Adarb2-), thus likely medial ganglionic eminence (MGE) derived inhibitory interneuron communities. Congruently, GABA _Adarb2+_1 selectively expressed *Vip*, [16, 17, 20, 28] and *Cnr*, which are known to be enriched in several Adarb2 positive corticohippocampal interneurons [6].

GABA _Adarb2+_2 was selectively enriched for the CGE interneuron associated markers *Jam2*, and *Lamp5* [20], and expressed *Sv2c* and *Reln*, previously used to denote several cortical interneuron subsets [3, 17], this cluster also strongly expressed *Cyct* and *Sema5a*.

In agreement with their presumed MGE origin the Adarb2 negative clusters GABA_Adarb2-_1-5 were all *Sox6* positive as previously described [20] and GABA _Adarb2-_1,2 and 4 , further expressed *Satb1*, a previously characterized driver of the terminal differentiation of MGE derived interneurons (Close et al., 2012). As expected these *Adarb2-*, *Sox6+* populations were also *Lhx6* positive albeit faintly in our dataset.

The canonical MGE interneuron subset marker *Sst* [16, 20, 28] was expressed robustly in GABA _Adarb2-_1. Notably, The distinct concomitant expression of *Sst*, *Nos1*, *Npy*, *Tacr1* and *Chodl*, observed in this cluster matches the transcriptional profile of cortico-hippocamapal, long-range projection interneurons [6]. However, the coexpression of *Sst*, and *Npy* has also been described in distinct subsets of striatal [34] and hypothalamic [35] GABAergic interneurons, brain regions which were also sampled in our study.

Only few cells in the neighbouring cluster GABA _Adarb2-_2 were *Sst* positive, however the concomitant expression of *Grik3*, *Elfn1* and *Lypd6* observed in this cluster was reminiscent of the transcriptional profile of *Sst* positive hippocamposeptal interneurons [6]. Of note, the coexpression of *Grik3* and *Elfn1* was also observed in GABAergic interneurons of the globus pallidus externus [11].
Several markers of previously described MGE interneuron subsets, such as *Etv1*, *Pde3a* [20], and *Eya1* [28], were selectively enriched in GABA _Adarb2-_3, which was also strongly *Dpf3* positive.
Interestingly, the axon guidance and progenitor cell proliferation master regulator *Slit 2* [36] was selectively enriched in GABA_Adarb2-_4. This *Pvalb* positive interneuron population was also enriched in *Eya4*, as well as *Rspo2* and *Esrrg*, known to be enriched in the amygdala and thalamus, respectively [6].
GABA_Adarb2-_5 interneurons distinctly coexpressed *Col5a2*, *Hs3st2*, *Uncb5* and *Vipr2*. *Vipr2* has been previously suggested as a marker of a distinct subset of *Pvalb* interneurons [20, 28]. However, in our dataset the *Vipr2* positive cluster only contained few *Pvalb* positive cells. Likewise, *Unc5b*, which was suggested as a marker of *Pvalb*+ chandelier cells [16], was in our dataset selectively expressed in cluster GABA_Adarb2-_5.
Strikingly, two GABAergic communities distinctly coexpressed *Rgs9*, *Rarb* and *Gng7*, markers which are largely restricted to the basal ganglia and the cortical sub plate [1, 9]. These clusters matched previously validated transcriptional profiles of striatal medium spiny projection neurons and were annotated GABAergic medium spiny neuron clusters 1 and 2 (GABA_MSN_1 and _2). GABA_MSN_1 selectively expressed *Tshz1*, which is known to be densely expressed in olfactory areas, the thalamus and the basal ganglia, while in the later it has been established as a marker of striosomal direct pathway MSNs [11, 37]. This cluster additionally robustly coexpressed, *Adarb2*, *Foxp2,* and *Olfm3* and might thus harbour a recently described, distinct *Foxp2/Olfm3* double positive striatal subpopulation [38], further corroborated by the selective expression of *Otof*, which among GABAergic neurons has been suggested as a marker of eccentric MSNs [11]. *Rgs9*, *Rarb*, *Gng7* and the basal ganglia associated orphan GPCR gene *Gpr149* [39] were expressed more prominently in GABA_MSN_2, which also expressed markers of canonical striatal medium spiny projection neurons such as *Ppp1r1b* and *Drd2* [6, 11].
In comparison, the remaining GABAergic community, lacked specific markers and did not match to known transcriptional interneuron and MSN identities, and was thus termed ambiguous GABAergic cell cluster (GABA_Amb). Most notably, this cluster distinctly coexpressed *Slc17a6*, which was found to be expressed in Gad1/2 positive cells of deep grey matter structures such as the hypothalamus [5, 35] and the cortical sub plate enriched gene *Glra3*. It is thus plausible that several non-cortical interneuron populations might have contributed to this cluster.

Subclustering of infarction enriched myeloid cells

Unsupervised subclustering of MCAO ipsi derived MCs (n= 2646 nuclei) using the first 10 PCs and a resolution of 0.4 corroborated the myeloid cell identity of this cluster revealing 6 subclusters (Suppl.Fig.3c-e). Two clusters (MG_0 and MG_1) where predominantly enriched in pan microglia markers such as *C1qa* and *Fcrls*, expressed the homeostatic microglia markers *Cx3cr1*, *P2ry12* and *Tmem119* [13, 40] to varying degrees, but where almost devoid of macrophage associated transcripts. Of note we observed a higher average expression of homeostatic microglia markers in the more M0-like cluster MG_0 than in MG_1. Three clusters, only sparsely expressed microglia marker genes, however where enriched in the macrophage associated transcripts *Apoe* and *Lyz2*, other macrophage associated transcripts such as *Cd14*, *Ccr2*, *Fn1* and *Cybb* where expressed to variable degrees in these cluster [14, 15]. We thus reasoned that these cluster are primarily derived from macrophage nuclei. However, previous work denoted an extensive overlap of the transcriptional signatures of microglia and central nervous system macrophages and a loss of microglia marker signatures under several pathological conditions, as well as acquisition of macrophage associated markers such as *Apoe* and *Lyz2* [14, 15, 40, 41]. Therefore, these clusters were annotated macrophage enriched clusters MΦe_1 to 3, conceding that a contribution of microglia to these subclusters cannot be excluded. The remaining cluster was strongly enriched for the dendritic cell associated marker *Flt3* [15] and accordingly most robustly expressed *Cd74* and several rat orthologues of the human major histocompatibility class II complex such as *RT1-Bb* and *RT1-Db1*. This cluster was therefore annotated dendritic cell cluster (DC).

Subclustering of oligodendrocyte lineage cells

Joint unsupervised sub clustering of OLIGO_1 and OLIGO_2, using the first 13 PCs and a clustering resolution of 0.3, revealed 12 sub clusters (Fig.2). Two sub clusters (total n= 639 nuclei) only faintly expressed canonical oligodendrocyte lineage markers but were enriched for *Rbfox3, Rarb, Gad2* and *Foxp2*, reminiscent of MSN neurons. Due to this biologically implausible marker combination these clusters were deemed to be clustering artefacts and removed from downstream analyses (Suppl.Fig.6a-d). Thereafter, the dataset was reclustered at the above described parameters. The remaining 10240 nuclei split into 10 sub clusters. 3 populations were enriched for the shared OPC and committed oligodendrocyte precursor (COP) associated markers *Sox6* and *Vcan* [7, 42, 43], of which 2 populations (OPC_0, OPC_1) also expressed canonical OPC markers such as *Pcdh15*, *Ptprz1*, *Pdgfra*, *Cspg4,* or *C1ql1* [6, 7, 43]. Branching of OPC_0 we identified one cluster with a residual *Pcdh15, Ptprz11* expression and strong enrichment for the canonical COP markers *Bmp4*, *Neu4* and *Tnr*, COP migration associated genes *Tns3* and *Fyn* and the immature oligodendrocyte marker *Bcas1* [6, 7, 44]. The COP cluster also strongly expressed genes, which have been shown to be upregulated upon the transition of COPs to newly formed oligodendrocytes (NFOLIGO), such as *Tcf7l2* or *Itpr2* [7]. Next to the COP cluster we identified one cluster positive for these genes, concomitantly expressing the *bona fide* NFOLIGO markers *Rras2* and *Cnksr3* [6]. These markers where residually expressed in the adjacent myelin forming oligodendrocyte cluster 1 (MFOLIGO_1). Both myelin forming Oligodendrocyte clusters (MFOLIGO_1 and MFOLIGO_2) exhibited a robust concomitant enrichment for the markers of myelin formation *Opalin, Serinc5, Mbp , Mobp, Mal, Mog, Plp1*, [6, 7, 11, 42, 43], as well as the early stage myelination associated marker *Tspan2* [45]. Importantly, these clusters distinctly lacked the expression of the late stage oligodendrocyte differentiation gene *Hapln2* [6, 9, 43] and only faintly expressed other mature oligodendrocyte associated genes, such as *Dock5* and *Apod* [7, 43]. The clusters co-expressing the established oligodendrocyte maturity markers *Dock5*, *Hapln2* and *Apod,* were termed mature oligodendrocyte clusters (MOLIGO_1 to 3). We identified one sub cluster coexpressing oligodendrocyte transcripts and immune cell (e.g. *Ptprc*, or *Fcrl2*) and immune process (e.g. *Cfh, Anxa3, Lyn*) associated genes. As detailed in the main text, previous research indicated the possibility of oligodendrocyte transcript phagocytosis by myeloid cells and enrichment of these transcripts within the nuclear compartment, in pathological conditions such as in MS lesions [46]. Congruently, particularly myeloid cell markers (e.g. *Ptprc*, *Fcrl2*), SAMC associated genes (e.g. *Gpnmb*, *Spp1*) and the phagocytosis related gene *Anxa3* [47], were derived from infarcted, but not Sham group brain tissue (Suppl.Fig.6e). However previous research also described the occurrence of immune process related genes in *bona fide* OPCs and oligodendrocytes termed immune oligodendroglia [42, 48, 49]. It is therefore possible that immune oligodendroglia transcript might have also contributed to this cluster. However, due to the obvious contribution of myeloid cells we opted to annotate this subcluster as a myeloid cell oligodendrocyte mixed cluster (MC_OLIGO).

Subclustering of astrocytes

Unsupervised sub clustering of astrocytes, using the first 14 principal components (PCs) and a clustering resolution of 0.3, revealed 8 sub clusters. Similar to the results of the oligodendrocyte subclustering analysis we observed two sub clusters, with evident neuronal transcript contamination (e.g. *Rbfox3*). We also observed one cluster which only weakly expressed pan-astrocyte markers, such as *Slc1a2*, *Slc1a3*, or *Gja1* or markers of reactive astrocytes, such as *Gfap* or *Aqp4*. Recent research provided evidence for the reprograming of mature oligodendrocytes into astrocytes, via a Plp+/GFAP+ "AO"-stage during acute brain injury [50]. However, within the astrocyte subclustering in our dataset the subcluster concomitantly expressing astrocyte and oligodendrocyte transcripts lacked reactive astrocyte marker genes, was conserved across all datasets and not associated to infarcted hemispheres. Furthermore, DEG analyses comparing the transcriptional profiles within this cluster between Sham hemispheres, infarcted hemispheres and hemispheres contralateral to infarction did not reveal any DEGs. Because we thereby excluded that this cluster harbors a stroke specific expressional signature in line with the AO-stage, we treated this cluster as a technical artefact and did not consider it in further calculations. In total we removed 225 nuclei from downstream analyses and the remaining 1233 nuclei were reclustered at the above described parameters, revealing 5 filtered sub clusters.

Astrocyte subclusters could be broadly split into homeostatic astrocyte gene enriched (e.g. *Gpc5*, *Kirrel3*, *Cdh10*, *Trpm3*) [46, 51, 52] sub clusters (AC_1 and AC_2) and reactive astrocyte clusters (*Gfap*, *Vim*, *Osmr*, *Cd44*, or *Cp*) [53, 54] (AC_3 to AC_5). AC_2 differed from AC_1 in the expression of *Slc7a10*, which is more enriched in astrocytes of the olfactory area and *Agt* and *Slc6a11* which are restricted to non telencephalic astrocyte subsets [6].

Supplementary References

1. Lein ES*, et al.* Genome-wide atlas of gene expression in the adult mouse brain. *Nature* **445**, 168-176 (2007).

2. Ximerakis M*, et al.* Single-cell transcriptomic profiling of the aging mouse brain. *Nat Neurosci* **22**, 1696-1708 (2019).

3. Velmeshev D*, et al.* Single-cell genomics identifies cell type-specific molecular changes in autism. *Science* **364**, 685-689 (2019).

4. Lake BB*, et al.* Neuronal subtypes and diversity revealed by single-nucleus RNA sequencing of the human brain. *Science* **352**, 1586-1590 (2016).

5. Mickelsen LE*, et al.* Single-cell transcriptomic analysis of the lateral hypothalamic area reveals molecularly distinct populations of inhibitory and excitatory neurons. *Nat Neurosci* **22**, 642-656 (2019).

6. Zeisel A*, et al.* Molecular Architecture of the Mouse Nervous System. *Cell* **174**, 999-1014.e1022 (2018).

7. Marques S*, et al.* Oligodendrocyte heterogeneity in the mouse juvenile and adult central nervous system. *Science* **352**, 1326-1329 (2016).

8. Batiuk MY*, et al.* Identification of region-specific astrocyte subtypes at single cell resolution. *Nat Commun* **11**, 1220 (2020).

9. Rosenberg AB*, et al.* Single-cell profiling of the developing mouse brain and spinal cord with split-pool barcoding. *Science* **360**, 176-182 (2018).

10. Fullard JF*, et al.* Single-nucleus transcriptome analysis of human brain immune response in patients with severe COVID-19. *Genome Med* **13**, 118 (2021).

11. Saunders A*, et al.* Molecular Diversity and Specializations among the Cells of the Adult Mouse Brain. *Cell* **174**, 1015-1030.e1016 (2018).

12. Duan L*, et al.* PDGFRβ Cells Rapidly Relay Inflammatory Signal from the Circulatory System to Neurons via Chemokine CCL2. *Neuron* **100**, 183-200.e188 (2018).

13. Hammond TR*, et al.* Single-Cell RNA Sequencing of Microglia throughout the Mouse Lifespan and in the Injured Brain Reveals Complex Cell-State Changes. *Immunity* **50**, 253-271.e256 (2019).

14. Beuker C*, et al.* Stroke induces disease-specific myeloid cells in the brain parenchyma and pia. *Nat Commun* **13**, 945 (2022).

15. Li X*, et al.* Single-cell transcriptomic analysis of the immune cell landscape in the aged mouse brain after ischemic stroke. *J Neuroinflammation* **19**, 83 (2022).

16. Hodge RD*, et al.* Conserved cell types with divergent features in human versus mouse cortex. *Nature* **573**, 61-68 (2019).

17. Lake BB*, et al.* Integrative single-cell analysis of transcriptional and epigenetic states in the human adult brain. *Nat Biotechnol* **36**, 70-80 (2018).

18. Pazarlar BA, Aripaka SS, Petukhov V, Pinborg L, Khodosevich K, Mikkelsen JD. Expression profile of synaptic vesicle glycoprotein 2A, B, and C paralogues in temporal neocortex tissue from patients with temporal lobe epilepsy (TLE). *Mol Brain* **15**, 45 (2022).

19. Huang Y*, et al.* Expression of transcription factor Satb2 in adult mouse brain. *Anat Rec (Hoboken)* **296**, 452-461 (2013).

20. Yao Z*, et al.* A taxonomy of transcriptomic cell types across the isocortex and hippocampal formation. *Cell* **184**, 3222-3241.e3226 (2021).

21. Cheng S*, et al.* Vision-dependent specification of cell types and function in the developing cortex. *Cell* **185**, 311-327.e324 (2022).

22. Zeisel A*, et al.* Brain structure. Cell types in the mouse cortex and hippocampus revealed by single-cell RNA-seq. *Science* **347**, 1138-1142 (2015).

23. Tomorsky J, Parker PRL, Doe CQ, Niell CM. Precise levels of nectin-3 are required for proper synapse formation in postnatal visual cortex. *Neural Dev* **15**, 13 (2020).

24. Clark EA*, et al.* Cortical RORβ is required for layer 4 transcriptional identity and barrel integrity. *Elife* **9**, (2020).

25. Lennon MJ, Jones SP, Lovelace MD, Guillemin GJ, Brew BJ. Bcl11b-A Critical Neurodevelopmental Transcription Factor-Roles in Health and Disease. *Front Cell Neurosci* **11**, 89 (2017).

26. Poplawski GHD*, et al.* Injured adult neurons regress to an embryonic transcriptional growth state. *Nature* **581**, 77-82 (2020).

27. Tsyporin J*, et al.* Transcriptional repression by FEZF2 restricts alternative identities of cortical projection neurons. *Cell Rep* **35**, 109269 (2021).

28. Tasic B*, et al.* Shared and distinct transcriptomic cell types across neocortical areas. *Nature* **563**, 72-78 (2018).

29. Erwin SR*, et al.* Spatially patterned excitatory neuron subtypes and projections of the claustrum. *Elife* **10**, (2021).

30. Shi Y*, et al.* Single-Nucleus RNA Sequencing Reveals that Decorin Expression in the Amygdala Regulates Perineuronal Nets Expression and Fear Conditioning Response after Traumatic Brain Injury. *Adv Sci (Weinh)* **9**, e2104112 (2022).

31. Ma S, Shen PJ, Burazin TC, Tregear GW, Gundlach AL. Comparative localization of leucine-rich repeat-containing G-protein-coupled receptor-7 (RXFP1) mRNA and [33P]-relaxin binding sites in rat brain: restricted somatic co-expression a clue to relaxin action? *Neuroscience* **141**, 329-344 (2006).

32. Liu S*, et al.* WIDE AWAKE mediates the circadian timing of sleep onset. *Neuron* **82**, 151-166 (2014).

33. Yaguchi K, Nishimura-Akiyoshi S, Kuroki S, Onodera T, Itohara S. Identification of transcriptional regulatory elements for Ntng1 and Ntng2 genes in mice. *Mol Brain* **7**, 19 (2014).

34. Muñoz-Manchado AB*, et al.* Diversity of Interneurons in the Dorsal Striatum Revealed by Single-Cell RNA Sequencing and PatchSeq. *Cell Rep* **24**, 2179-2190.e2177 (2018).

35. Steuernagel L*, et al.* HypoMap-a unified single-cell gene expression atlas of the murine hypothalamus. *Nat Metab* **4**, 1402-1419 (2022).

36. Gonda Y, Namba T, Hanashima C. Beyond Axon Guidance: Roles of Slit-Robo Signaling in Neocortical Formation. *Front Cell Dev Biol* **8**, 607415 (2020).

37. Xiao X*, et al.* A Genetically Defined Compartmentalized Striatal Direct Pathway for Negative Reinforcement. *Cell* **183**, 211-227.e220 (2020).

38. Lee H*, et al.* Cell Type-Specific Transcriptomics Reveals that Mutant Huntingtin Leads to Mitochondrial RNA Release and Neuronal Innate Immune Activation. *Neuron* **107**, 891-908.e898 (2020).

39. Sjöstedt E*, et al.* An atlas of the protein-coding genes in the human, pig, and mouse brain. *Science* **367**, (2020).

40. Mendes MS, Majewska AK. An overview of microglia ontogeny and maturation in the homeostatic and pathological brain. *Eur J Neurosci* **53**, 3525-3547 (2021).

41. Sousa C*, et al.* Single-cell transcriptomics reveals distinct inflammation-induced microglia signatures. *EMBO Rep* **19**, (2018).

42. Jäkel S*, et al.* Altered human oligodendrocyte heterogeneity in multiple sclerosis. *Nature* **566**, 543-547 (2019).

43. Hilscher MM, Langseth CM, Kukanja P, Yokota C, Nilsson M, Castelo-Branco G. Spatial and temporal heterogeneity in the lineage progression of fine oligodendrocyte subtypes. *BMC Biol* **20**, 122 (2022).

44. Fard MK*, et al.* BCAS1 expression defines a population of early myelinating oligodendrocytes in multiple sclerosis lesions. *Sci Transl Med* **9**, (2017).

45. Birling MC, Tait S, Hardy RJ, Brophy PJ. A novel rat tetraspan protein in cells of the oligodendrocyte lineage. *J Neurochem* **73**, 2600-2608 (1999).

46. Schirmer L*, et al.* Neuronal vulnerability and multilineage diversity in multiple sclerosis. *Nature* **573**, 75-82 (2019).

47. Junker H*, et al.* Proteomic identification of an upregulated isoform of annexin A3 in the rat brain following reversible cerebral ischemia. *Glia* **55**, 1630-1637 (2007).

48. Falcão AM*, et al.* Disease-specific oligodendrocyte lineage cells arise in multiple sclerosis. *Nat Med* **24**, 1837-1844 (2018).

49. Kirby L*, et al.* Oligodendrocyte precursor cells present antigen and are cytotoxic targets in inflammatory demyelination. *Nat Commun* **10**, 3887 (2019).

50. Bai X*, et al.* In the mouse cortex, oligodendrocytes regain a plastic capacity, transforming into astrocytes after acute injury. *Dev Cell* **58**, 1153-1169.e1155 (2023).

51. Absinta M*, et al.* A lymphocyte-microglia-astrocyte axis in chronic active multiple sclerosis. *Nature* **597**, 709-714 (2021).

52. Habib N*, et al.* Disease-associated astrocytes in Alzheimer's disease and aging. *Nat Neurosci* **23**, 701-706 (2020).

53. Zamanian JL*, et al.* Genomic analysis of reactive astrogliosis. *J Neurosci* **32**, 6391-6410 (2012).

54. Liddelow SA*, et al.* Neurotoxic reactive astrocytes are induced by activated microglia. *Nature* **541**, 481-487 (2017).
