# Supplementary material for: Single-nucleus RNA sequencing reveals glial cell type-specific responses to ischemic stroke in male rodents
Source: Nat Commun. 2024 Jul 24;15:6232. doi: 10.1038/s41467-024-50465-z (PMC11266704; doi:10.1038/s41467-024-50465-z)
Supplement: Supplementary file 14 — Reporting Summary [file 41467_2024_50465_MOESM14_ESM.pdf]

nature portfolio

Corresponding author(s): Michael Mildner, Hendrik Jan Ankersmit

Last updated by author(s): Jun 26, 2024

Reporting Summary

Nature Portfolio wishes to improve the reproducibility of the work that we publish. This form provides structure for consistency and transparency in reporting. For further information on Nature Portfolio policies, see our Editorial Policies and the Editorial Policy Checklist.

Statistics

For all statistical analyses, confirm that the following items are present in the figure legend, table legend, main text, or Methods section.

n/a	Confirmed	
	x	The exact sample size (*n*) for each experimental group/condition, given as a discrete number and unit of measurement
	x	A statement on whether measurements were taken from distinct samples or whether the same sample was measured repeatedly
	x	The statistical test(s) used AND whether they are one- or two-sided<br>*Only common tests should be described solely by name; describe more complex techniques in the Methods section.*
x		A description of all covariates tested
	x	A description of any assumptions or corrections, such as tests of normality and adjustment for multiple comparisons
	x	A full description of the statistical parameters including central tendency (e.g. means) or other basic estimates (e.g. regression coefficient) AND variation (e.g. standard deviation) or associated estimates of uncertainty (e.g. confidence intervals)
	x	For null hypothesis testing, the test statistic (e.g. *F*, *t*, *r*) with confidence intervals, effect sizes, degrees of freedom and *P* value noted<br>*Give P values as exact values whenever suitable.*
x		For Bayesian analysis, information on the choice of priors and Markov chain Monte Carlo settings
	x	For hierarchical and complex designs, identification of the appropriate level for tests and full reporting of outcomes
x		Estimates of effect sizes (e.g. Cohen's *d*, Pearson's *r*), indicating how they were calculated

Our web collection on statistics for biologists contains articles on many of the points above.

Software and code

Policy information about availability of computer code

Data collection	For single nucleus RNA seq data raw data was generated via paired-end sequencing, with dual indexing (read length 50bp) using a NovaSeq 6000 (Illumina, San Diego, CA, USA). Demultiplexing and alignment of reads was performed using the Cellranger v.7.0.0 pipeline.
Data analysis	Bioinformatics analyses were conducted using R and R Studio (R version v.4.2.2). Unless otherwise stated, all computational snRNAseq analyses were carried out within the environment of the Seurat package v.4.3.0. Additionally, the following R packages were used: SoupX v1.6.2, DoubletFinder v2.0.3, Monocle3 v.1.3.1, decoupleR 2.7.1, LIgand-receptor ANalysis frAmework (LIANA) v.0.1.12, as well as the Enrichr Database.<br>The following R packages were used for data visualization: Seurat v.4.3.0, Monocle3 v.1.3.1, ggplot2 v.3.2.2 , EnhancedVolcano v.1.16.0, UpSetR v.1.4.0, scCustomize v.1.1.1, SCPubr v. 2.0.1, ComplexHeatmap v. 2.14.0, pheatmap v. 1.0.12, bioRender, Sketchbook v.5.1(Autodesk Inc., San Rafael, CA, USA), Affinity Designer v.1.10.6.1665 and Affinity Photo v.1.10.5.1342 (Serif, Nottingham, United Kingdom).<br>All analyses were conducted using the cited packages, according to publicly available instructions, provided by the developers. No novel costume code, or novel data analysis tools have been generated.<br>Immunofluorescence image data analyses were carried out using QuPath v.0.4.3. Hyperintense stroke lesion on T2 weighted MRI images were quantified using ImageJ v.1.54. GraphPad Prism v.9.0.0 was used for statistical analyses.

For manuscripts utilizing custom algorithms or software that are central to the research but not yet described in published literature, software must be made available to editors and reviewers. We strongly encourage code deposition in a community repository (e.g. GitHub). See the Nature Portfolio guidelines for submitting code & software for further information.

Data

Policy information about availability of data

All manuscripts must include a data availability statement. This statement should provide the following information, where applicable:

- Accession codes, unique identifiers, or web links for publicly available datasets
- A description of any restrictions on data availability
- For clinical datasets or third party data, please ensure that the statement adheres to our policy

All single nucleus RNA-seq datasets reported in the present paper have been deposited at the NCBI-GEO database under the GEO accession number: GSE250245. Additional source data underlying all reported bioniformatical analyses, as well as IF stainings and in vitro experiments are provided in the supplementary data and source data files.

Research involving human participants, their data, or biological material

Policy information about studies with human participants or human data. See also policy information about sex, gender (identity/presentation), and sexual orientation and race, ethnicity and racism.

Reporting on sex and gender	Archived brain tissue sections from N=1 male and N=3 female cases have been included. Sex refers to sex assigned at birth.
Reporting on race, ethnicity, or other socially relevant groupings	All archived brain tissue sections included in this study were derived from caucasian donors, further information on ethnicity or socially relevant groupings are not available.
Population characteristics	All included brain tissue sections were derived from donors between the age of 33 and 62 years and staged as infarcted tissue in the stage of advanced macrophage resorption and beginning of pseudo cystic cavity formation, by trained neuropathologists.
Recruitment	Archived tissue samples were derived from biopsy tissue which was bio banked after completion of all routine clinical diagnostic procedures, from patients how have provided informed consent. No compensation was offered to the donors.
Ethics oversight	Archived human biopsy derived brain tissue material was used in agreement with the Medical University of Vienna ethics committee votes: EK1636/2019, EK1454/2018).

Note that full information on the approval of the study protocol must also be provided in the manuscript.

Field-specific reporting

Please select the one below that is the best fit for your research. If you are not sure, read the appropriate sections before making your selection.

[x] Life sciences [ ] Behavioural & social sciences [ ] Ecological, evolutionary & environmental sciences

For a reference copy of the document with all sections, see nature.com/documents/nr-reporting-summary-flat.pdf

Life sciences study design

All studies must disclose on these points even when the disclosure is negative.

Sample size	No formal statistical sample size calculation was performed to determine sample size for in vivo experiments. The biological N for single nucleus RNA sequencing and validating immunofluorescence and cell culture assays was extrapolated from previous work, with similar research questions and study designs.
Data exclusions	None of the collected biological samples was excluded from subsequent analysis. During snRNAseq analysis nuclei with < 500 UMI counts, <250 or >5000 expressed genes and > 5% mitochondrial genes expressed, were removed from downstream analysis. All genes with less than 3 UMI counts per feature and all mitochondrial genes were removed from downstream analyses. Furthermore, potential nuclei doublets and ambient RNA signal was removed using DoubletFinder v2.0.3 and SoupX v1.6.2, using the instructions provided by the respective developers.
Replication	For single nucleus RNA sequencing studies tissue from a total of n= 4 Sham operated rats and n= 7 MCAO operated rats was used. For Immunofluorescence stainings tissue from a total of n= 4 Sham operated rats, n= 5 MCAO operated rats and 4 MCAO operated miced was used. Tissue from a total of N = 20 E20 fetal rats, derived from three timed pregnant Sprague Dawley rat dams was used for cell culture assays. The number of migrated NG2+ cells was assessed in 3 independent experiments, with 3-4 replicates (where one independent well constitutes one technical replicate) per group and experiment. The percentages of Ki67 positive cells was assessed in 2 independent experiments with 3-4 independent replicates per group and experiment. The BrdU assay was performed twice independently, assessing the percentage of BrdU positive cells in 3-4 replicates per group and experiment. Sample size for human pathohistology case studies (N =4) reported in the supplementary materials was limited by available archived tissue.
Randomization	Animals were randomly allocated to the MCAO and Sham control groups.
Blinding	MRI Lesion volumes were determined by a trained investigator blinded to condition. However ischemic lesions are macroscopically evident on MRI data. Cell counts obtained from cell culture assays were determined by blinded investigators. Cell counts obtained from immunofluorescence stainings of tissue sections were determined using a standardized, automated approach using QuPath software. On

tissue sections ischemic lesions are macroscopically evident and corresponding MRI data were used to define regions of interest, therefore investigators could not be blinded to condition while performing these image analyses.

Reporting for specific materials, systems and methods

We require information from authors about some types of materials, experimental systems and methods used in many studies. Here, indicate whether each material, system or method listed is relevant to your study. If you are not sure if a list item applies to your research, read the appropriate section before selecting a response.

Materials & experimental systems

n/a	Involved in the study
☐	☒ Antibodies
☒	☐ Eukaryotic cell lines
☒	☐ Palaeontology and archaeology
☐	☒ Animals and other organisms
☒	☐ Clinical data
☒	☐ Dual use research of concern
☒	☐ Plants

Methods

n/a	Involved in the study
☒	☐ ChIP-seq
☒	☐ Flow cytometry
☐	☒ MRI-based neuroimaging

Antibodies

Antibodies used

Rabbit polyclonal anti-NG2: Merck Millipore (Burlington, MA, USA) - Cat# AB5320, RRID:AB_91789.
Rabbit anti-NG2 -Cy3® conjugated: Merck Millipore (Burlington, MA, USA) - Cat# AB5320C3, RRID:AB_11203295.
Rabbit anti-BrdU -FITC conjugated: BD Bioscience (Franklin Lakes, NJ, USA) From kit: Cat# 558662.
Rabbit polyclonal anti-Ki67: Abcam (Cambridge, UK) - Cat# ab15580, RRID:AB_443209.
Rabbit polyclonal anti-CD44: Abcam (Cambridge, UK) - Cat# ab157107, RRID:AB_2847859.
Recombinant rabbit monoclonal anti-Iba1: Abcam (Cambridge, UK) - Cat# ab178846, RRID:AB_2636859.
Chicken polyclonal anti-GFAP: Abcam (Cambridge, UK) - Cat# ab4674, RRID:AB_304558.
Rabbit polyclonal anti-Osteopontin: Abcam (Cambridge, UK) - Cat# ab63856, RRID:AB_1524127.
Mouse monoclonal IgG1 anti-Vimentin, Clone V9: DAKO -Agilent Technologies (Santa Clara, CA, USA) - Cat# M0725, RRID:AB_10013485.
Rabbit monoclonal anti-Vimentin antibody: Abcam (Cambridge, UK) - Cat# ab92547, RRID:AB_10562134.
Rabbit recombinant monoclonal anti-IL33: Abcam (Cambridge, UK) - Cat# ab187060, RRID:AB_2894704.
Rabbit recombinant monoclonal anti-IL33: Abcam (Cambridge, UK) - Cat# ab207737, RRID:AB_2827630.
Rabbit monoclonal anti-PDGF Receptor α: Cell Signaling Technology (Danvers, MA, USA)- Cat# D1E1E XP® #3174, RRID:AB_2162345.
Mouse monoclonal anti-MAP2: Merck Millipore (Burlington, MA, USA) - Cat#: MAB3418, RRID:AB_94856.
Goat Anti-Rabbit IgG H&L (Alexa Fluor® 488): Abcam (Cambridge, UK) - Cat# ab150077, RRID:AB_2630356
Goat anti-Mouse IgG1 Cross-Adsorbed Secondary Antibody (Alexa Fluor® 546): Thermo Fisher Scientific (Waltham, MA, USA)- Cat# A-21123, RRID:AB_2535765.
Goat anti-Chicken IgY (H+L) Secondary Antibody (Alexa Fluor® 647) Thermo Fisher Scientific (Waltham, MA, USA) -Cat# A-21449, RRID:AB_2535866.
A full list of all antibodies is also given within the Supplementary Information provided in this paper in Supplementary Table 2.

Validation

Catalog numbers an RRIDs, all used dilutions and all additional eptiope retrival details for all antibodies used within this study are given within supplementary tables 2 and 3 in the supplementary information. All relevant validation information provided by the manufacturer, as well as studies referencing the use of the antibody can be retrieved via the provided Catalog number and RRID information given. Second step only, or linker protein only controls were run in parallel to all stainings within this study.

Animals and other research organisms

Policy information about studies involving animals; ARRIVE guidelines recommended for reporting animal research, and Sex and Gender in Research

Laboratory animals

In vivo experiments were performed on male Wistar rats (6 weeks at receipt, ± 30g, Janvier Lab, Le Genest-Sainte-Isle) and male C57BL/6J mice (6-7 weeks at receipt, BW: 20-25g, Janvier Lab, Le Genest-Sainte-Isle). For the generation of primary rat glial cells E20 fetal rat cortices, derived from pregnant Sprague Dawley rat dams (Charles river) were used.

Wild animals

No wild animals were included within this study.

Reporting on sex

All animals included in in vivo experiments were male, to reduce variability within the study cohort. Potential limitations associated to this approach are mentioned explicitly within the main text of the paper.

Field-collected samples

No field-collected samples were included within this study.

Ethics oversight | The protocol was submitted for ethic approval to the French Ministry of Research and the ethical committee (CENOMEXA – registered under the reference CENOMEXA-C2EA – 54) and received the agreement numbers #36435 and #36135.

Note that full information on the approval of the study protocol must also be provided in the manuscript.

Plants

Seed stocks | N/A

Novel plant genotypes | N/A

Authentication | N/A

Magnetic resonance imaging

Experimental design

Design type | No functional MRI (fMRI) data were acquired.

Design specifications | n/a

Behavioral performance measures | n/a

Acquisition

Imaging type(s) | Structural MRI

Field strength | 7T

Sequence & imaging parameters | T2-weighted images were acquired using a multislice multiecho sequence: TE/TR 33 ms/2500 ms, on a Pharmascan 7T MRI system, using surface coils (Bruker, Germany)

Area of acquisition | Whole brain scan

Diffusion MRI ☐ Used ☒ Not used

Preprocessing

Preprocessing software | n/a

Normalization | n/a

Normalization template | n/a

Noise and artifact removal | n/a

Volume censoring | n/a

Statistical modeling & inference

Model type and settings | MRI was conducted to confirm that middle cerebral artery occlusion (MCAO) induced T2 hyper intense lesions in MCAO as opposed to Sham operated animals. Lesion sizes were quantified on raw T2 images using ImageJ software. Lesion volumes were determined by a trained investigator blinded to condition and are expressed in mm3. Beyond the descriptive report of lesion sizes no statistical comparisons have been performed.

Effect(s) tested | n/a

Specify type of analysis: ☒ Whole brain ☐ ROI-based ☐ Both

Statistic type for inference | n/a

(See Eklund et al. 2016)

Correction | n/a

Models & analysis

n/a | Involved in the study
- [x] | Functional and/or effective connectivity
- [x] | Graph analysis
- [x] | Multivariate modeling or predictive analysis
