# Supplementary material for: Single-nucleus RNA sequencing reveals glial cell type-specific responses to ischemic stroke in male rodents
Source: Nat Commun. 2024 Jul 24;15:6232. doi: 10.1038/s41467-024-50465-z (PMC11266704; doi:10.1038/s41467-024-50465-z)
Supplement: Supplementary file 2 — Description of Additional Supplementary Files [file 41467_2024_50465_MOESM2_ESM.pdf]

# Description of Additional Supplementary Files

File Name: **Supplementary Data 1**

Description: **Nuclei counts and cluster markers for all cell clusters.** This data file contains the absolute and relative nuclei numbers for all major cell clusters, infarcted hemisphere derived myeloid cell subclusters, oligodendrocyte lineage cell subclusters and astrocyte subclusters. Furthermore, this file contains all raw “FindAllMarkers” - MAST outputs from which cluster markers for each main cluster and each subcluster, were derived, for all performed clustering and subclustering analyses. For further details on the performed calculations and nomenclature the reader is referred to the Methods and Results sections of the main text.

File Name: **Supplementary Data 2**

Description: **Signature gene sets derived from previous studies.** This data file contains all gene set lists used for the comparisons of DEGs derived from this dataset with previously published gene sets and the calculation of module scores.

File Name: **Supplementary Data 3**

Description: **Results of differentially expressed gene calculations, for each major cell cluster.** This data file contains all DEG counts and non-subset DEG lists (= raw Seurat - “FindMarkers” - MAST outputs) for each major cell cluster, for various group comparisons (detailed in the table of contents within the file). Details on the performed calculations and nomenclature are given in the Methods and Results sections of the main text.

File Name: **Supplementary Data 4**

Description: **Results of differentially expressed gene calculations, for oligodendrocyte lineage sub clusters.** This data file contains DEGs derived from the comparisons of oligodendrocyte lineage subclusters OPC_1 vs OPC_0 and MOLIGO_1 vs MOLIGO_2, as well as all DEG counts and non-subset DEG lists (= raw Seurat - “FindMarkers” - MAST outputs) for each conserved subcluster, for various group comparisons (detailed in the table of contents within the file). Details on the performed calculations and nomenclature are given in the Methods and Results sections of the main text.

File Name: **Supplementary Data 5**

Description: **Functional annotation of infarction restricted oligodendrocyte subclusters.** EnrichR output tables were generated from inputting DEGs (Bonferrioni adjusted p values <0.05 & log2FC >0.6, or log2FC < -0.6) derived from OPC_1 vs. OPC_0 and MOLIGO_1 vs. MOLIGO_2 comparisons. EnrichR outputs generated from upregulated DEGs are termed UP_OPC_1 and UP_MOLIGO_1 and outputs generated from downregulated DEGs are termed DOWN_OPC_1 and DOWN_MOLIGO_1, respectively, followed by the queried database (e.g. GO_Biological_Process_2023). Binary gene by term matrices show which UP or DOWN regulated DEGs are associated to which enriched terms. Only terms with Adjusted.P.value < 0.05 were included in the binary matrices. Up to Top 20 enriched terms, sorted by descending EnrichR Combined.Scores were included in the binary matrices.

File Name: **Supplementary Data 6**

Description: **Monocle3 derived gene modules for oligodendrocyte subclusters.** This data file contains all Monocle3 derived gene modules derived from genes which robustly change as a function of pseudotime (q-values < 0.05 and morans I >0.05). Further details on the performed calculations are given in the Methods section of the main text.

File Name: **Supplementary Data 7**

Description: **Summary of cell count comparisons derived from immunofluorescences stainings of rat brain tissue.** This data file contains the results of all between group comparisons of immunopositive cell counts within perilesional grey matter (GM), affected white matter (WM) and ischemic core regions of interest (ROI), in rat coronal brain sections. Cell counts were derived from immunofluorescence stainings, colocalizing various targets of interest, as detailed within the file. For all comparisons Kruskal-Wallis-H-Tests, followed by Dunn's post hoc comparisons were performed.

File Name: **Supplementary Data 8**

Description: **Summary of cell count comparisons derived from immunofluorescences stainings of mouse brain tissue.** This data file contains the results of all between group comparisons of immunopositve cell counts within grey matter (GM) white matter (WM) and lesion core (core) regions of interest (ROI), in murine coronal brain sections. Cell counts were derived from immunofluorescence stainings, colocalizing various targets of interest, as detailed within the file. For all comparisons Mann-Whitney U tests were performed.

File Name: **Supplementary Data 9**

Description: **Results of differentially expressed gene calculations, for reactive astrocyte sub clusters.** This data file contains DEG lists (= raw Seurat - “FindMarkers” - MAST outputs) derived from the comparisons of each reactive astrocyte subclusters (AC_3, AC_4, AC_5) to the homeostatic astrocyte clusters (AC_1 and AC_2 pooled). Details on the performed calculations and nomenclature are given in the Methods and Results sections of the main text.

File Name: **Supplementary Data 10**

Description: **Functional annotation of reactive astrocyte subclusters.** EnrichR output tables were generated from inputting DEGs (adjusted p values <0.05 & log2FC >0.6, or log2FC < -0.6) derived from AC_3 vs. homeostatic Astrocytes (= AC_1 & AC_2, pooled), AC_4 vs. homeostatic Astrocytes and AC_5 vs. homeostatic astrocytes comparisons. EnrichR outputs generated from upregulated DEGs are termed UP_AC_3, UP_AC_4 and UP_AC_5 and outputs generated from downregulated DEGs are termed DOWN_AC_3, DOWN_AC_4 and DOWN_AC_5, respectively, followed by the queried database (e.g. GO_Biological_Process_2023). Binary gene by term matrices show which UP or DOWN regulated DEGs are associated to which enriched terms. Only terms with Adjusted.P.value < 0.05 were included in the binary matrices. Up to Top 20 enriched terms, sorted by descending EnrichR Combined.Scores were included in the binary matrices.

File Name: **Supplementary Data 11**

Description: **Results of Cell-cell communication (CCC) inference analysis.** This data file contains all significant inferred ligand receptor (LR) pairs, between glial and myeloid cell (MC) subclusters, within each group (Sham, MCAO contra and MCAO ipsi), as calculated using the LIANA pipeline. Only LR-pairs with aggregate rank scores ≤ 0.05 were retained. Due to the small number of nuclei in the myeloid cell main cluster within the Sham and MCAO contra groups, myeloid cells were not further subclustered within these groups. Further methodological details on the performed calculations, study design and nomenclature are given in the Methods and Results sections of the main text.
